# Design, Synthesis, and Antiproliferative Evaluation of Novel Coumarin/2-Cyanoacryloyl Hybrids as Apoptosis Inducing Agents by Activation of Caspase-Dependent Pathway

**DOI:** 10.3390/molecules23081972

**Published:** 2018-08-07

**Authors:** Yu-Ying Zhang, Qian-Qian Zhang, Jia-Li Song, Liang Zhang, Cheng-Shi Jiang, Hua Zhang

**Affiliations:** 1 School of Biological Science and Technology, University of Jinan, Jinan 250022, China; yuyingzhang2008@163.com (Y.-Y.Z.); zhangqian464x@163.com (Q.-Q.Z.); 13285419800@163.com (J.-L.S.); 2 The Key Laboratory of Animal Resistant Biology of Shandong College of Life Sciences, Shandong Normal University, Jinan 250014, China; zhangrijing@126.com

**Keywords:** coumarin, 2-cyanoacryloyl, antiproliferative activity, apoptosis, caspase

## Abstract

A series of novel coumarin/2-cyanoacryloyl hybrids were prepared and evaluated for their in vitro anticancer activity. Among them, two analogs **5p** and **5q** showed promising antiproliferative activity against a panel of cancer cell lines, including A549, H157, HepG2, MCF7, MG63, and U2OS. Particularly, **5q** showed the most potent activity towards MG63 cells with an IC_50_ value of 5.06 ± 0.25 μM. Morphological observation and 4,6-diamidino-2-phenylindole (DAPI) staining assay showed that **5q**-treated MG63 cells displayed significant apoptosis characteristics. Moreover, flow cytometric detection of phosphatidylserine externalization revealed that **5q** induced MG63 apoptosis in a dose-dependent manner. Real-time PCR and western blot assay further confirmed that **5q** had strong effects to induce MG63 cell apoptosis, suggesting that the action was associated with down-regulation of the anti-apoptotic protein Bcl-2, upregulation of pro-apoptotic protein Bax, and induced activation of caspase-3, 8, and 9. The present results provide a new chemotype for anticancer drug development and continuing investigation into candidates with coumarin/2-cyanoacryloyl scaffold is warranted.

## 1. Introduction

Cancer is one of the most leading health hazards and the prominent cause of death worldwide [1]. In the past several decades, a number of anticancer drugs have been developed for the clinical treatment of various cancerous diseases, but none of them is perfectly sufficient due to the multidrug resistance and the high incidence of side effects, such as cardiotoxicity, diarrhea, and neutropenia [2,3]. This status makes the development of new anticancer medicines urgently necessary.

**Figure 1:**
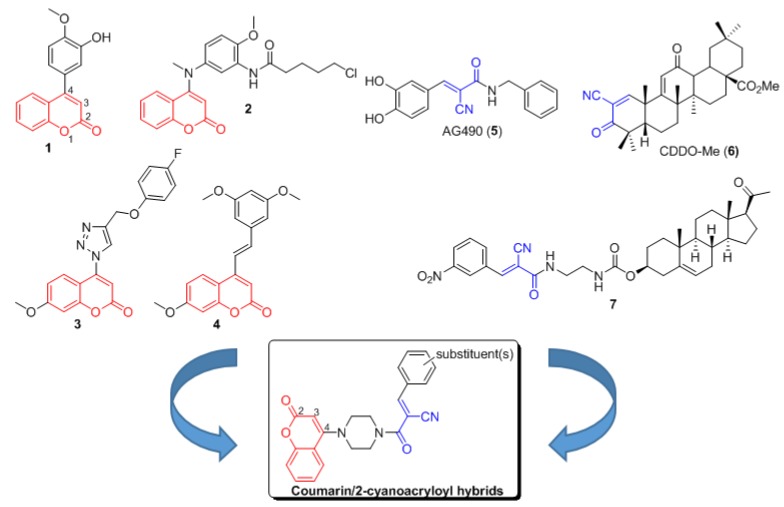
Reported anticancer agents **1**–**7** and designed coumarin/2-cyanoacryloyl hybrids.

Over the last thirty years, natural product-derived novel anticancer drugs have undoubtedly occupied the dominating position in drug development [4]. Coumarins represent a large class of naturally occurring secondary metabolites from several plant families [5] and they exhibit various pharmacological activities, including anticancer [6], antimicrobial [7], lipid-lowering [8], and antiviral activities [9]. The promising biological profile has stimulated medicinal chemists’ great interest in designing coumarin-based anticancer agents [10]. It has been reported that the substitution pattern on coumarin core is vital to their therapeutic application, and frequently the coumarin derivatives with substitution at C-4 position exhibit promising anticancer activity [11]. For instance, compound **1** showed potent antiproliferative activity against HBL100 cell line [12]; compound **2** was a promising antimitotic agent and showed a range of in vitro and in vivo anticancer activity [13]; coumarin/1,2,3-triazol hybrid **3** displayed antitumor activity via cell arrest at G2/M phase and apoptosis induction [14]; and, compound **4** [15] showed good antiproliferative activity that was endowed with an apoptosis-inducing capability (Figure 1).

The α,β-unsaturated ketone (Michael acceptor) pharmacophore, which was widely existent in numerous bioactive compounds, could form adducts with reactive thiol groups of proteins to induce modification and misfolding of protein, consequently resulting in a variety of pharmacological activities [16]. Especially, the incorporation of a Michael acceptor was believed to be capable of increasing anticancer activity [17,18]. As a Michael acceptor, 2-cyanoacryloyl pharmacophore has been extensively applied in the design of anticancer drugs. For example, tyrphostin AG490 (**5**), being clinically used as an anticancer agent, can significantly block in vitro and in vivo leukemic cell growth [19]; CDDO-Me (**6**, bardoxoline methyl), which is a semi-synthetic oleanane triterpenoid, acting as Nrf2 activator and NF-κB inhibitor, has been used for the treatment of leukemia and solid tumors [20]; compound **7** recently reported by our laboratory showed potent antiproliferative activity against a panel of cancer cell lines [21].

Molecule hybridization is a powerful strategy to afford new chemical entities (NCEs) with anticancer profiles [22,23,24,25]. In the present study, we assumed that the merging of coumarin and 2-cyanoacryloyl moieties in a single chemical entity was able to drive new potential anticancer drug candidates. As the piperazine ring is one of the most privileged heterocyclics that is found in plethora of Food and Drug Administration (FDA) approved medications [26,27,28], it was incorporated as a linkage into the C-4 position of coumarin in our design (Figure 1). Herein, we report the synthesis of a series of molecular hybridization-based coumarin/2-cyanoacryloyl derivatives, their anticancer activity evaluation, and mechanism of action study.

## 2. Results and Discussion

### 2.1. Chemistry

**Scheme 1:**
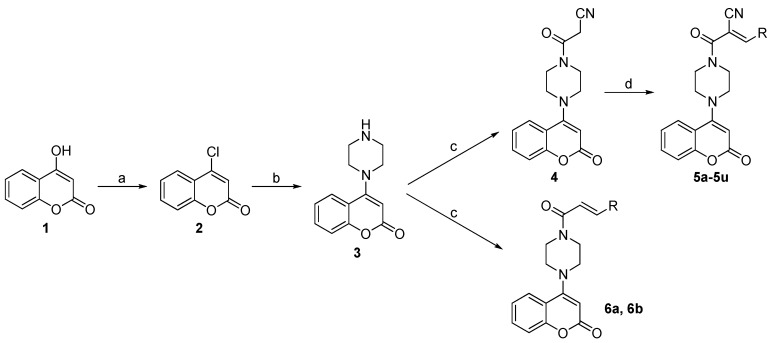
The general procedure for the synthesis of **5a**–**5u**, **6a**, and **6b**. Reagents and conditions: (a) POCl_3_, CH_3_CN, BTEAC, 80 °C, 6 h; (b) piperizine, EtOH, r.t., overnight; (c) corresponding acid, 2-(7-aza-1*H*-benzotriazole-1-yl)-1,1,3,3-tetramethyluronium hexafluorophosphate (HATU), *N*,*N*-diisopropylethylamine, CH_2_Cl_2_, r.t., 4 h; and, (d) corresponding aldehyde, piperidine, AcOH, EtOH, 80 °C, overnight.

The synthetic route to the target compounds **5a**–**5u**, **6a**, and **6b** is shown in Scheme 1. Briefly, commercially available 4-hydroxy-2*H*-chromen-2-one (**1**) as starting material was reacted with phosphorus oxychloride (POCl_3_) to give compound **2** in 78.5% yield in the presence of benzyltriethylammonium chloride (BTEAC). Then, compound **3** was prepared in 60% yield from **2** by reacting with piperazine in ethanol at room temperature. Intermediate **4** was prepared by reaction of **3** with the corresponding acid. Further, aldol condensation of compound **4** with corresponding aldehyde to produce the target compounds **5a**–**5u** in the presence of piperidine and acetic acid at 80 °C. In addition, another two target compounds **6a** and **6b** were prepared, according to the same protocol as that of **4**. Literature survey indicated that the aldol condensation of aromatic aldehydes with 2-cyanoacetamide fragment usually led to olefinic products with E geometry, as shown in **5a**–**5u** [29,30,31], and the E-configuration of the newly formed double bonds in **6a** and **6b** was supported by the large coupling constants (*J* = 15.4 Hz). In addition, no mixtures of E/Z isomers were observed by NMR and HPLC analyses. The ^1^H, ^13^C NMR, and HR-MS spectra for **5a**–**5u**, **6a** and **6b** can be found in supplementary materials.

### 2.2. Antiproliferative Activity

The synthesized coumarin/2-cyanoacryloyl hybrids **5a**–**5u** were initially evaluated for their antiproliferative activity against the human A549 cancer cell line at 20 μM, while using MTT assay. The compounds with an inhibition ratio >50% at 20 μM were further tested for their IC_50_ values. Doxorubicin was used as a positive control. The results were shown in Table 1. The intermediates **3** and **4** lacking the α,β-unsaturated ketone moiety were inactive towards A549, while nine of 21 target compounds exhibited obviously antiproliferative activity against A549 with IC_50_ values ranging from 18.92 to 6.26 μM, with compound **5p** showing the strongest activity. These results indicated that the 2-cyanoacryloyl moiety played an important role in their bioactivity. Compounds **5a** to **5i** possessing electron-donating groups (such as -Me, -NMe_2_, -OEt, etc.) showed very weak inhibitory activity, with an inhibition ratio of less than 50% at 20 μM. Interestingly, most compounds with halogen atom (F, Cl, and Br), such as **5j**–**5l** and **5n**–**5s** showed significant growth inhibition, revealing that the halogen atom in benzene ring was vital to their activity. Our previous structure-activity relationship (SAR) study on a series of pregnenolone/2-cyanoacryloyl conjugates indicated that the electron-withdrawing substituents at meta-position of benzene ring was able to increase cytotoxicity [21]. On the contrary, in the present work, the activity of coumarin/2-cyanoacryloyl hybrids from **5s** to **5u** decreased or lost with the electron-withdrawing effect increasing.

The electron-withdrawing effect of cyano could increase the reactivity of α,β-unsaturated ketone and the bioactivity of compounds, which had been well proved by the SAR study of CDDO-Me [32,33]. To verify whether the cyano group could increase cytotoxicity for this series of hybrids, two more compounds **6a** and **6b** without cyano in α,β-unsaturated ketone system were prepared as the analogs of **5p** and **5q**, respectively. As expected, both **6a** and **6b** showed relatively weak cytotoxicity towards A549 at 20 μM with an inhibition ratio of 45.92% and 25.83%, respectively, indicating that the cyano group did play a key role in increasing their activity.

While considering the above in vitro IC_50_ values and preliminary SAR study in A549 model, we further elected analogs with halogen atom, such as **5j**–**5s**, **6a**, and **6b**, for in vitro antiproliferative evaluation against H157, HepG2, MCF7, MG63, and U2OS cancer cells. From Table 2, it is very interesting that compounds **5p** and **5q** still presented IC_50_ values less than 20 μM towards all of the tested cancer cells. Particularly, compound **5q** served as the most potent compound for the MG63 cell proliferation inhibitory effect with an IC_50_ value of 5.06 ± 0.25 μM. Other analogues did not show broad-spectrum anticancer activity at 20 μM. In addition, analogs **6a** and **6b** without CN group were inactive towards all of the tested cancer cells, which was consistent with the SAR results that were obtained in the anticancer evaluation on A549 model.

### 2.3. Compound **5q** Induced Morphological Changes and Promoted Apoptosis of MG63 Cells

**Figure 2:**
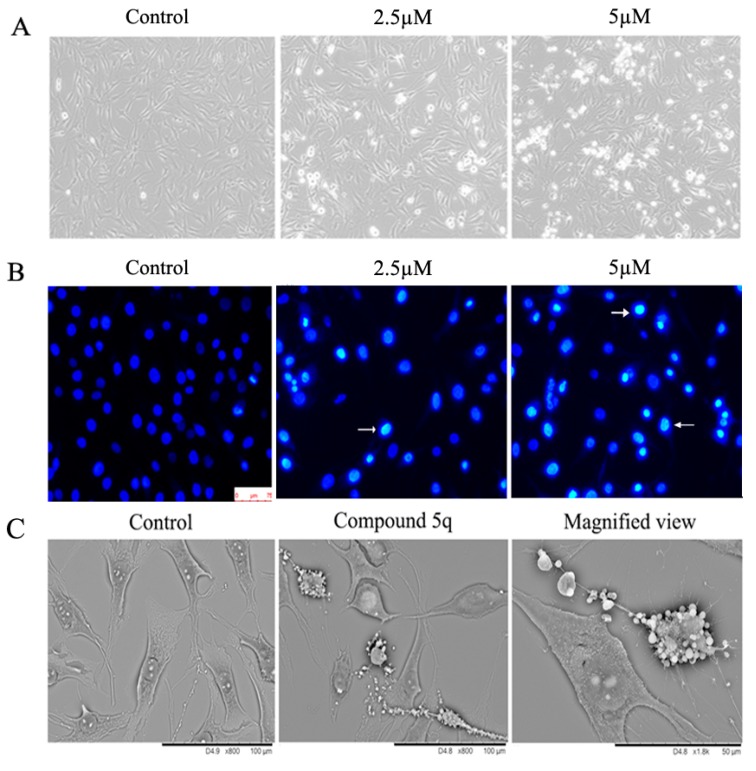
Morphological observation of **5q**-induced apoptosis in MG63 cell. (**A**) MG63 cells were exposed to **5q** for 24 h and optical microscope was used for morphological analysis; (**B**) Fluorescent micrographs of **5q**-treated and untreated MG63 cells after 4,6-diamidino-2-phenylindole (DAPI) staining. MG63 cells treated with 0, 2.5, and 5 µM **5q** for 24 h, were subjected to DAPI staining and then viewed under a fluorescent microscope at magnification of 200. Arrows indicate apoptotic characteristics of treated cells; and, (**C**) screening electron microscope (SEM) micrographs of 5 µM **5q**-treated MG63 cells for 24 h.

Herein, compound **5q** was chosen as a prototype for anticancer mechanism of action study on MG63 cell model. Osteosarcoma MG63 cells were treated with 2.5 and 5 μM of **5q**, and then cellular morphology was observed under phase contrast microscope. Figure 2A showed that **5q** induced remarkable changes of cellular morphology, such as cell shrinkage and loss of cellular architecture. To examine the effect of **5q** on cell morphology during cell death, morphological changes of control cells and treated cells were observed by fluorescent microscopy while using the DNA-binding fluorescent dye DAPI. The results from Figure 2B showed that **5q**-treated MG63 cells exhibited obvious apoptosis characteristics, including nuclear fragmentation, chromatin compaction, cell shrinkage, and membrane integrity loss or deformation of late apoptotic appearance. This observation demonstrated that these cells were undergoing apoptosis. Additionally, the results of screening electron microscope (SEM) also showed that 5 μM **5q**-treated cells displayed typical apoptotic morphology. As shown in Figure 2C, the nuclear convolution and fragmentation in membrane-enclosed bodies were accumulated in MG63 cells, while little apoptotic bodies were detected in untreated control cells.

**Figure 3:**
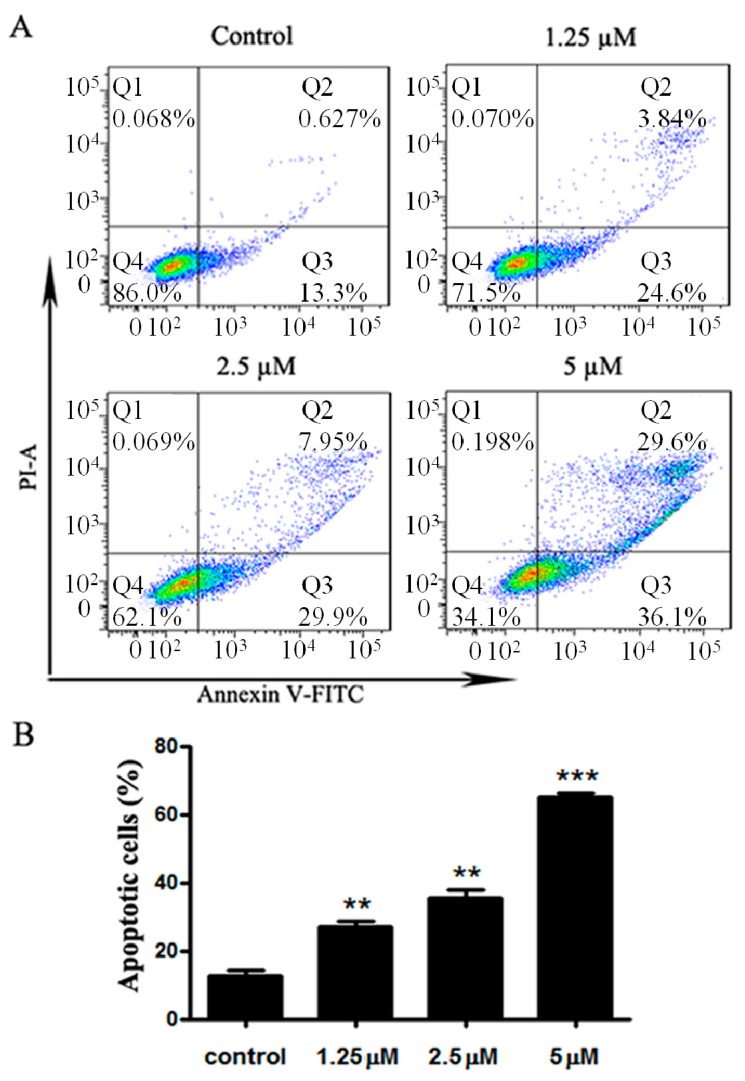
Flow cytometry analysis of MG63 cell apoptosis by double staining with annexin V-FITC and PI. Values are presented as mean ± SD of three independent experiments. ** *p* < 0.01 vs. normal control, *** *p* < 0.001 vs. normal control.

To further quantify apoptosis induced by **5q**, MG63 cells were stained with Annexin V-FITC and propidium iodide (PI), and the results were subsequently analyzed using flow cytometry. As shown in Figure 3A, the proportion of apoptotic cells was increased in the **5q**-treated group after exposure for 24 h, the rate of apoptotic cells were 12.6% for control (0.2% DMSO), 27.7% for 1.25 µM, 35.5% for 2.5 µM, and 65.8% for 5 µM **5q**, respectively (Figure 3B). MG63 cells that were treated with **5q** exhibited a significant increment in the number of apoptotic cells in a dose-dependent way.

### 2.4. Apoptosis Induced by **5q** Was Caspase-Dependent in MG63 Cells

**Figure 4:**
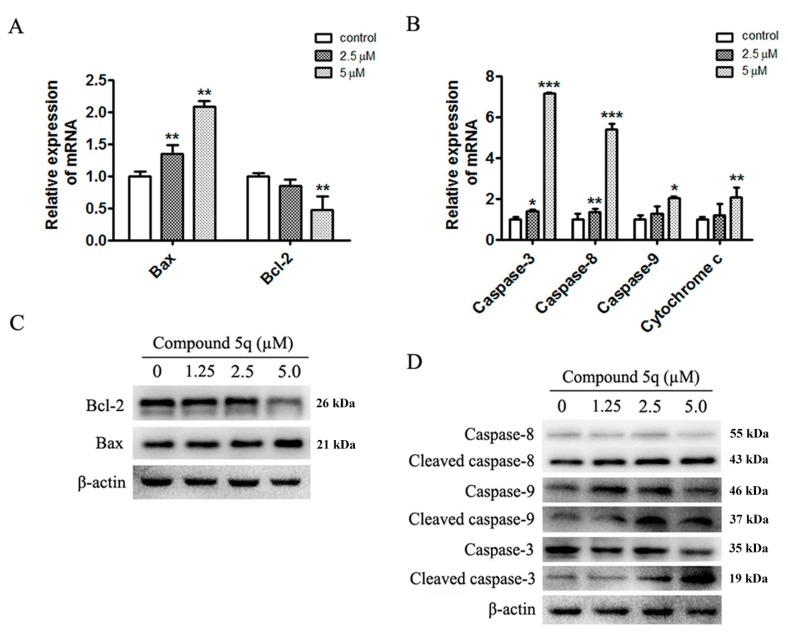
Effect of **5q** on apoptosis regulators. (**A**,**B**) Real-time PCR analysis for Bax, Bcl-2, caspase-3, caspase-8, caspase-9 and cytochrome c in **5q**-treated cells. Data were represented as mean ± SD (n = 3), * *p* < 0.05, ** *p* < 0.01, *** *p* < 0.001 as compared to control cells. (**C**,**D**) MG63 cells were treated with indicated concentrations of **5q** for 24 h and protein levels of Bax, Bcl-2, caspases-3, 8, and 9 were analyzed by western blotting.

To clarify the molecular mechanism that is involved with apoptosis inducement, we measured the expression of Bcl-2 and Bax in **5q**-treated MG63 cells by quantitative PCR and western blot. With the administration of different concentrations of **5q** for 24 h (Figure 4A), the expression of Bcl-2, a gene involved in the suppression of apoptosis [34], was decreased, while the expression of Bax that was involved in the promotion of apoptosis was increased. The decreased expression of Bcl-2 and increased expression of Bax were also confirmed at protein level (Figure 4C).

In addition, the activation of caspases, which are a family of cysteine aspartyl-specific proteases, is a characteristic feature of both extrinsic and intrinsic apoptosis pathways [35,36]. Therefore, we determined the expression of caspase-3, 8, and 9. The mRNA levels of caspase-3, 8, and 9 upon the application of 5 µM **5q** were much higher than the control (Figure 4B). Furthermore, the release of cytochrome c into cytosol is the key step to induce apoptosis [37,38], and, as shown in Figure 4C, the mRNA of cytochrome c was obviously increased in the treated group. Subsequently, the effects of **5q** treatment on caspase-3, 8, and 9 activity in MG63 cells were investigated (Figure 4D). The results showed that treatment with **5q** strongly triggered the cleavage of caspase-3, 8, and 9 at 24 h, illustrating the activation of the caspase signaling pathway.

## 3. Materials and Methods 

### 3.1. Chemistry

Commercially available reagents were used without further purification. Organic solvents were evaporated with reduced pressure using a Büchi R-100 evaporator (Büchi, Flawil, Switzerland). Reactions were monitored by TLC using Yantai JingYou (Yantai, China) GF254 silica gel plates. Silica gel column chromatography was performed on Biotage Isolera One (Biotage, Uppsala, Sweden). Melting points were measured by a Melting Point YRY-3 apparatus (Tianjin Precision Apparatus Factory, Tianjin, China). The purity of the samples was determined by an analytical Agilent 1260 HPLC (Agilent, Waldbronn, Germany) with ZDRBAX SB-C18 column (4.6 × 150 mm) using parameters as follows: H_2_O/MeOH, 40/60 to 0/100 in 15 min, plus 5 min isocratic MeOH, flow rate at 1.0 mL/min, λ = 254 and 280 nm. NMR spectra were measured on Bruker Avance III 600 MHz spectrometer (Bruker, Fällanden, Switzerland). Chemical shifts were expressed in *δ* (ppm) and coupling constants (*J*) in Hz with residual solvent signals as references (CDCl_3_, *δ*_H_ 7.26 ppm and *δ*_C_ 77.0 ppm; DMSO-*d*_6_, *δ*_H_ 2.50 ppm and *δ*_C_ 39.5 ppm). ESIMS (electrospray ionization mass spectrometry) analyses were performed on an Agilent 1260-6460 Triple Quard LC-MS instrument (Agilent, Waldbronn, Germany). HR-ESIMS data were acquired on an Agilent Q-TOF 6520 spectrometer (Agilent, Waldbronn, Germany).

#### 3.1.1. 4-Chloro-2*H*-Chromen-2-One (**2**)

A solution of 4-hydroxycoumarin (**1**, 6.15 g, 38 mmol) and BTEAC (34.50 g, 152 mmol) in CH_3_CN (120 mL) was stirred at 40 °C for 30 min. POCl_3_ (23.25 g, 152 mmol) was added dropwise and then the mixture was heated at 80 °C for 6 h under N_2_. The solution was added to water and stirred at room temperature for 1 h. The resulting black precipitate was filtered and then dissolved in EtOAc. The solution was dried over anhydrous MgSO_4_. After being filtered, the solution was concentrated in vacuum to give **2** (5.40 g, 79% yield) as a pale yellow solid. Mp. 89–91 °C. HPLC purity: 95.9%, t_R_ = 11.77 min. ^1^H NMR (600 MHz, CDCl_3_) *δ* 7.88 (dd, *J* = 8.2, 1.5 Hz, 1H), 7.62 (ddd, *J* = 8.7, 7.5, 1.5 Hz, 1H), 7.38 (ddd, *J* = 7.6, 6.0, 1.2 Hz, 2H), 6.62 (s, 1H). ^13^C NMR (150 MHz, CDCl_3_) *δ* 159.2, 153.1, 149.8, 133.4, 125.6, 125.0, 118.1, 117.2, 115.6. MS (ESI): *m*/*z* calcd for C_9_H_6_ClO_2_ [M + H]^+^ 181.0, found 181.1.

#### 3.1.2. 3-Oxo-3-(4-(2-Oxo-2*H*-Chromen-4-yl)Piperazin-1-yl)Propanenitrile (**4**)

To a solution of 2-cyanoacetic acid (1.53 g, 18 mmol) in dry CH_2_Cl_2_ (30 mL) was added HATU (6.86 g, 18 mmol) and *N*,*N*-diisopropylethylamine (2.78 g, 21.6 mmol) at room temperature. After 0.5 h, compound **3** (4.14 g, 18 mmol) was added, and the reaction mixture was stirred at room temperature for 16 h. The volatiles were removed under reduced pressure, and the residue was partitioned between EtOAc and H_2_O. The organic layers were combined, dried over MgSO_4_ and evaporated to afford a crude residue. The crude product was further purified by flash column chromatography while using a mixture of CH_2_Cl_2_-MeOH (50:1) to yield product **4** as a white solid (4.10 g, 70% yield). Mp. 224–226 °C. HPLC purity: 96.3%, t_R_ = 6.76 min. ^1^H NMR (600 MHz, DMSO-*d*_6_) *δ* 7.75 (dd, *J* = 8.0, 1.4 Hz, 1H), 7.61 (ddd, *J* = 8.3, 7.7, 1.4 Hz, 1H), 7.39 (dd, *J* = 8.3, 0.9 Hz, 1H), 7.35 (ddd, *J* = 8.0, 7.7, 0.9 Hz, 1H), 5.73 (s, 1H), 4.12 (s, 2H), 3.70 (t, *J* = 5.0 Hz, 2H), 3.60 (t, *J* = 4.9 Hz, 2H), 3.29 (t, *J* = 4.9 Hz, 2H), 3.26 (t, *J* = 4.9 Hz, 2H). ^13^C NMR (150 MHz, DMSO-*d*_6_) *δ* 161.8, 161.0, 160.2, 153.6, 131.9, 125.4, 123.8, 117.3, 116.1, 115.6, 97.0, 50.1, 50.1, 44.8, 41.2, 24.9. HRMS (ESI): *m*/*z* calcd for C_16_H_16_N_3_O_3_ [M + H]^+^ 298.1186, found 298.1188.

#### 3.1.3. General Procedures for Synthesis of **5a**–**5u**

To a mixture of **4** (100 mg, 0.31 mmol) in 3 mL EtOH was added 10 drops of piperidine and corresponding aldehyde (0.34 mmol). The mixture was stirred at 80 °C for 6 h and then concentrated under reduced pressure. The residue was purified by flash column chromatography to afford the desired products **5a**–**5u**, respectively.

*(E)-2-(4-(2-Oxo-2H-Chromen-4-yl)Piperazine-1-Carbonyl)-3-Phenylacrylonitrile* (**5a**). White solid (96 mg, 80.4% yield). Mp. 160–162 °C. HPLC purity: 95.1%, t_R_ = 12.70 min. ^1^H NMR (600 MHz, CDCl_3_) *δ* 7.92 (d, *J* = 8.0 Hz, 2H), 7.85 (s, 1H), 7.61 (dd, *J* = 8.0, 1.1 Hz, 1H), 7.53 (m, 4H), 7.37 (d, *J* = 8.2 Hz, 1H), 7.29 (dd, *J* = 8.0, 7.6 Hz, 1H), 5.78 (s, 1H), 3.96 (t, *J* = 4.9 Hz, 4H), 3.34 (t, *J* = 4.9 Hz, 4H). ^13^C NMR (150 MHz, CDCl_3_) *δ* 163.4, 162.2, 160.7, 154.4, 153.7, 132.9, 132.1, 132.1, 130.4, 129.4, 124.5, 123.9, 118.1, 116.2, 116.1, 105.4, 99.4, 50.9. HRMS (ESI): *m*/*z* calcd for C_23_H_20_N_3_O_3_ [M + H]^+^ 386.1499, found 386.1491.

*(E)-2-(4-(2-Oxo-2H-Chromen-4-yl)Piperazine-1-Carbonyl)-3-p-Tolylacrylonitrile* (**5b**). White solid (71 mg, 57.4% yield). Mp. 154–156 °C. HPLC purity: 97.6%, t_R_ = 13.51 min. ^1^H NMR (600 MHz, CDCl_3_) *δ* 7.82 (m, 3H), 7.61 (dd, *J* = 8.0, 1.2 Hz, 1H), 7.53 (ddd, *J* = 8.4, 7.8, 1.2 Hz, 1H), 7.35 (d, *J* = 8.4 Hz, 1H), 7.29 (m, 3H), 5.77 (s, 1H), 3.95 (t, *J* = 4.9 Hz, 4H), 3.33 (t, *J* = 4.9 Hz, 4H), 2.42 (s, 3H). ^13^C NMR (150 MHz, CDCl_3_) *δ* 163.7, 162.1, 160.7, 154.3, 153.8, 144.0, 132.0, 130.5, 130.1, 129.4, 124.5, 123.9, 118.1, 116.5, 116.1, 103.9, 99.3, 50.9, 21.9. HRMS (ESI): *m*/*z* calcd for C_24_H_22_N_3_O_3_ [M + H]^+^ 400.1656, found 400.1663.

*(E)-3-(3,4-Dimethylphenyl)-2-(4-(2-oxo-2H-Chromen-4-yl)Piperazine-1-Carbonyl)Acrylonitrile* (**5c**). White solid (59 mg, 46.1% yield). Mp. 180–182 °C. HPLC purity: 98.6%, t_R_ = 14.49 min. ^1^H NMR (600 MHz, CDCl_3_) *δ* 7.78 (s, 1H), 7.69 (d, *J* = 8.0 Hz, 1H), 7.65 (s, 1H), 7.61 (d, *J* = 8.0 Hz, 1H), 7.52 (dd, *J* = 8.3, 7.6 Hz, 1H), 7.34 (d, *J* = 8.3 Hz, 1H), 7.28 (dd, *J* = 8.0, 7.6 Hz, 1H), 7.24 (d, *J* = 8.0 Hz, 1H), 5.76 (s, 1H), 3.95 (t, *J* = 4.9 Hz, 4H), 3.33 (t, *J* = 4.9 Hz, 4H), 2.32 (s, 3H), 2.31 (s, 3H). ^13^C NMR (150 MHz, CDCl_3_) *δ* 163.8, 162.1, 160.7, 154.3, 153.9, 142.8, 137.8, 132.0, 131.6, 130.6, 129.8, 128.0, 124.5, 123.8, 118.0, 116.5, 116.1, 103.6, 99.2, 50.8, 20.2, 19.9. HRMS (ESI): *m*/*z* calcd for C_25_H_24_N_3_O_3_ [M + H]^+^ 414.1812, found 414.1820.

*(E)-3-(4-(Dimethylamino)Phenyl)-2-(4-(2-oxo-2H-Chromen-4-yl)Piperazine-1-Carbonyl)Acrylonitrile* (**5d**). Yellow solid (78 mg, 58.8% yield). Mp. 234–236 °C. HPLC purity: 95.4%, t_R_ = 12.50 min. ^1^H NMR (600 MHz, CDCl_3_) *δ* 7.89 (d, *J* = 9.0 Hz, 2H), 7.79 (s, 1H), 7.62 (dd, *J* = 8.0, 1.2 Hz, 1H), 7.52 (ddd, *J* = 8.3, 7.6, 1.2 Hz, 1H), 7.35 (d, *J* = 8.3 Hz, 1H), 7.28 (dd, *J* = 8.0, 7.6 Hz, 1H), 6.69 (d, *J* = 9.0 Hz, 2H), 5.77 (s, 1H), 3.95 (t, *J* = 4.9 Hz, 4H), 3.34 (t, *J* = 4.9 Hz, 4H), 3.10 (s, 9H). ^13^C NMR (150 MHz, CDCl_3_) *δ* 165.4, 162.2, 160.8, 154.4, 154.4, 153.3, 133.3, 131.9, 124.6, 123.8, 120.0, 118.4, 118.1, 116.2, 111.6, 99.1, 96.2, 51.0, 40.1. HRMS (ESI): *m*/*z* calcd for C_25_H_25_N_4_O_3_ [M + H]^+^ 429.1921, found 429.1930.

*(E)-3-(4-(Diethylamino)Phenyl)-2-(4-(2-oxo-2H-Chromen-4-yl)Piperazine-1-Carbonyl)Acrylonitrile* (**5e**). Yellow solid (76 mg, 53.7% yield). Mp. 188–190 °C. HPLC purity: 95.4%, t_R_ = 14.30 min. ^1^H NMR (600 MHz, CDCl_3_) *δ* 7.87 (d, *J* = 9.0 Hz, 2H), 7.78 (s, 1H), 7.62 (dd, *J* = 8.0, 1.0 Hz, 1H), 7.52 (ddd, *J* = 8.3, 7.6, 1.0 Hz, 1H), 7.36 (d, *J* = 8.3 Hz, 1H), 7.28 (dd, *J* = 8.0, 7.6 Hz, 1H), 6.67 (d, *J* = 9.0 Hz, 2H), 5.77 (s, 1H), 3.95 (t, *J* = 4.9 Hz, 4H), 3.45 (q, *J* = 7.1 Hz, 4H), 3.33 (t, *J* = 4.9 Hz, 4H), 1.23 (t, *J* = 7.1 Hz, 6H). ^13^C NMR (150 MHz, CDCl_3_) *δ* 165.6, 162.3, 160.9, 154.4, 154.4, 150.3, 133.7, 131.9, 124.6, 123.8, 119.4, 118.6, 118.1, 116.2, 111.2, 99.1, 95.3, 51.0, 44.9, 12.7. HRMS (ESI): *m*/*z* calcd for C_27_H_29_N_4_O_3_ [M + H]^+^ 457.2234, found 457.2240.

*(E)-3-(2-METHOXYPHENYL)-2-(4-(2-oxo-2H-Chromen-4-yl)Piperazine-1-Carbonyl)Acrylonitrile* (**5f**). White solid (75 mg, 58.3% yield). Mp. 216–218 °C. HPLC purity: 97.7%, t_R_ = 12.79 min. ^1^H NMR (600 MHz, CDCl_3_) *δ* 8.28 (s, 1H), 8.19 (dd, *J* = 7.8, 1.4 Hz, 1H), 7.61 (dd, *J* = 8.0, 1.3 Hz, 1H), 7.53 (ddd, *J* = 8.3, 7.6, 1.3 Hz, 1H), 7.49 (ddd, *J* = 8.4, 7.6, 1.4Hz, 1H), 7.36 (dd, *J* = 8.3, 0.8 Hz, 1H), 7.29 (ddd, *J* = 8.0, 7.6, 1.0 Hz, 1H), 7.06 (dd, *J* = 7.8, 7.6 Hz, 1H), 6.96 (d, *J* = 8.4 Hz, 1H), 5.78 (s, 1H), 3.94 (t, *J* = 5.0 Hz, 4H), 3.89 (s, 3H), 3.34 (t, *J* = 5.0 Hz, 4H). ^13^C NMR (150 MHz, CDCl_3_) *δ* 163.9, 162.1, 160.8, 158.7, 154.3, 148.0, 134.4, 132.0, 128.8, 124.5, 123.9, 121.2, 121.0, 118.1, 116.5, 116.1, 111.3, 104.8, 99.3, 55.9, 50.9. HRMS (ESI): *m*/*z* calcd for C_24_H_22_N_3_O_4_ [M + H]^+^ 416.1605, found 416.1612.

*(E)-3-(3-Methoxyphenyl)-2-(4-(2-oxo-2H-Chromen-4-yl)Piperazine-1-Carbonyl)Acrylonitrile* (**5g**). Yellow solid (22 mg, 17.1% yield). Mp. 100–102 °C. HPLC purity: 95.6%, t_R_ = 12.84 min. ^1^H NMR (600 MHz, CDCl_3_) *δ* 7.80 (s, 1H), 7.61 (d, *J* = 7.9 Hz, 1H), 7.54 (dd, *J* = 8.3, 7.6 Hz, 1H), 7.51 (s, 1H), 7.45 (d, *J* = 7.7 Hz, 1H), 7.41 (dd, *J* = 8.1, 7.7 Hz, 1H), 7.36 (d, *J* = 8.3 Hz, 1H), 7.29 (dd, *J* = 7.9, 7.6 Hz, 1H), 7.08 (d, *J* = 8.1 Hz, 1H), 5.78 (s, 1H), 3.95 (t, *J* = 4.7 Hz, 4H), 3.86 (s, 1H), 3.34 (t, *J* = 4.7 Hz, 4H). ^13^C NMR (150 MHz, CDCl_3_) *δ* 163.4, 162.1, 160.7, 160.1, 154.4, 153.6, 133.3, 132.1, 130.4, 124.5, 123.9, 123.4, 119.4, 118.2, 116.2, 116.1, 114.2, 105.5, 99.4, 55.6, 50.9. HRMS (ESI): *m*/*z* calcd for C_24_H_22_N_3_O_4_ [M + H]^+^ 416.1605, found 416.1604.

*(E)-3-(3,5-Dimethoxyphenyl)-2-(4-(2-oxo-2H-Chromen-4-yl)Piperazine-1-Carbonyl)Acrylonitrile* (**5h**). White solid (25 mg, 18.1% yield). Mp. 182–184 °C. HPLC purity: 95.7%, t_R_ = 13.71 min. ^1^H NMR (600 MHz, CDCl_3_) *δ* 7.74 (s, 1H), 7.61 (dd, *J* = 8.0, 1.4 Hz, 1H), 7.54 (ddd, *J* = 8.3, 7.8, 1.4 Hz, 1H), 7.37 (dd, *J* = 8.3, 0.9 Hz, 1H), 7.29 (ddd, *J* = 8.0, 7.8, 1.0 Hz, 1H), 7.07 (d, *J* = 2.2 Hz, 2H), 6.63 (t, *J* = 2.2 Hz, 1H), 5.78 (s, 1H), 3.95 (t, *J* = 4.9 Hz, 4H), 3.84 (s, 6H), 3.34 (t, *J* = 5.0 Hz, 4H). ^13^C NMR (150 MHz, CDCl_3_) *δ* 163.3, 162.1, 161.2, 160.7, 154.4, 153.7, 133.6, 132.1, 124.5, 123.9, 118.2, 116.2, 116.1, 108.0, 105.8, 105.5, 99.4, 55.7, 50.9. HRMS (ESI): *m*/*z* calcd for C_25_H_24_N_3_O_5_ [M + H]^+^ 446.1710, found 446.1714.

*(E)-3-(2,3-Dihydrobenzo[b][1,4]Dioxin-6-yl)-2-(4-(2-oxo-2H-Chromen-4-yl)Piperazine-1-Carbonyl)Acrylonitrile* (**5i**). Yellow solid (44 mg, 32.0% yield). Mp. 216–218 °C. HPLC purity: 95.3%, t_R_ = 13.79 min. ^1^H NMR (600 MHz, CDCl_3_) *δ* 7.73 (s, 1H), 7.61 (dd, *J* = 8.0, 1.3 Hz, 1H), 7.53 (m, 2H), 7.45 (d, *J* = 8.5 Hz, 1H), 7.36 (dd, *J* = 8.3, 0.8 Hz, 1H), 7.28 (ddd, *J* = 8.0, 7.6, 0.8 Hz, 1H), 6.95 (d, *J* = 8.5 Hz, 1H), 5.77 (s, 1H), 4.33 (m, 2H), 4.29 (m, 2H), 3.94 (t, *J* = 4.9 Hz, 4H), 3.33 (t, *J* = 4.9 Hz, 4H). ^13^C NMR (150 MHz, CDCl_3_) *δ* 164.0, 162.1, 160.7, 154.3, 153.4, 148.0, 143.9, 132.0, 125.7, 125.2, 124.5, 123.9, 119.4, 118.2, 118.1, 116.7, 116.1, 102.4, 99.3, 64.9, 64.2, 50.9. HRMS (ESI): *m*/*z* calcd for C_23_H_22_N_3_O_5_ [M + H]^+^ 444.1554, found 444.1556.

*(E)-3-(2-Fluorophenyl)-2-(4-(2-oxo-2H-Chromen-4-yl)Piperazine-1-Carbonyl)Acrylonitrile* (**5j**). Yellow solid (47 mg, 37.6% yield). Mp. 162–164 °C. HPLC purity: 95.2%, t_R_ = 12.67 min. ^1^H NMR (600 MHz, CDCl_3_) *δ* 8.27 (m, 1H), 8.07 (s, 1H), 7.61 (dd, *J* = 8.0, 1.4 Hz, 1H), 7.56–7.51 (m, 2H), 7.36 (dd, *J* = 8.3, 1.0 Hz, 1H), 7.31–7.27 (m, 2H), 7.20–7.16 (m, 1H), 5.78 (s, 1H), 3.95 (t, *J* = 5.0 Hz, 4H), 3.35 (t, *J* = 5.0 Hz, 4H). ^13^C NMR (150 MHz, CDCl_3_) *δ* 162.9, 162.1, 160.7, 154.4, 144.6 (d, *J* = 7.2 Hz), 134.6 (d, *J* = 9.1 Hz), 132.0, 128.7, 125.0 (d, *J* = 3.7 Hz), 124.5, 123.9, 120.5 (d, *J* = 11.0 Hz), 118.1, 116.4, 116.3, 116.1, 115.7, 107.5 (d, *J* = 2.0 Hz), 99.5, 50.8. HRMS (ESI): *m*/*z* calcd for C_23_H_19_FN_3_O_3_ [M + H]^+^ 404.1405, found 404.1402.

*(E)-3-(3-Fluorophenyl)-2-(4-(2-oxo-2H-Chromen-4-yl)Piperazine-1-Carbonyl)Acrylonitrile* (**5k**). Yellow solid (80 mg, 64.0% yield). Mp. 188-190 °C. HPLC purity: 96.8%, t_R_ = 12.71 min. ^1^H NMR (600 MHz, CDCl_3_) *δ* 7.80 (s, 1H), 7.67 (d, *J* = 7.9 Hz, 1H), 7.64 (dt, *J* = 9.5, 2.0 Hz, 1H), 7.60 (dd, *J* = 8.0, 1.3 Hz, 1H), 7.54 (ddd, *J* = 8.3, 7.2, 1.3 Hz, 1H), 7.48 (m, 1H), 7.36 (dd, *J* = 8.3, 0.9 Hz, 1H), 7.29 (ddd, *J* = 8.0, 7.2, 0.9 Hz, 1H), 7.26–7.22 (m, 1H), 5.78 (s, 1H), 3.95 (t, *J* = 4.9 Hz, 4H), 3.34 (t, *J* = 4.9 Hz, 4H). ^13^C NMR (150 MHz, CDCl_3_) *δ* 163.7, 162.9, 162.1, 160.7, 154.4, 152.0, 152.0, 133.9 (d, *J* = 7.8 Hz), 132.1, 131.1 (d, *J* = 8.1 Hz), 126.3 (d, *J* = 3.0 Hz), 124.4, 123.9, 118.1, 116.7 (d, *J* = 22.8 Hz), 116.1, 115.7, 107.0, 99.5, 50.8. HRMS (ESI): *m*/*z* calcd for C_23_H_19_FN_3_O_3_ [M + H]^+^ 404.1405, found 404.1413.

*(E)-3-(4-Fluoro-3-Methylphenyl)-2-(4-(2-oxo-2H-Chromen-4-yl)Piperazine-1-Carbonyl)Acrylonitrile* (**5l**). White solid (37 mg, 28.6% yield). Mp. 174–176 °C. HPLC purity: 96.6%, t_R_ = 14.61 min. ^1^H NMR (600 MHz, CDCl_3_) *δ* 7.80 (m, 3H), 7.61 (d, *J* = 8.0 Hz, 1H), 7.54 (dd, *J* = 8.3, 7.8 Hz, 1H), 7.37 (d, *J* = 8.3 Hz, 1H), 7.29 (t, *J* = 8.0, 7.8 Hz, 1H), 7.12 (dd, *J* = 9.1, 8.8 Hz, 1H), 5.78 (s, 1H), 3.95 (t, *J* = 4.5 Hz, 4H), 3.34 (t, *J* = 4.9 Hz, 4H), 2.34 (s, 3H). ^13^C NMR (150 MHz, CDCl_3_) *δ* 164.7, 163.5, 163.0, 162.2, 160.7, 154.4, 152.8, 134.1 (d, *J* = 6.4 Hz), 132.1, 130.3 (d, *J* = 9.1 Hz), 128.2 (d, *J* = 3.7 Hz), 126.6 (d, *J* = 18.0 Hz), 124.5, 123.9, 118.2, 116.4, 116.3 (d, *J* = 4.5 Hz), 116.1, 104.4, 99.4, 50.9, 14.7 (d, *J* = 3.3 Hz). ^13^C NMR (150 MHz, CDCl_3_) *δ* 163.9 (d, *J* = 255.6 Hz), 163.5, 162.2, 160.7, 154.4, 152.8, 134.1 (d, *J* = 6.4 Hz), 132.1, 130.3 (d, *J* = 9.1 Hz), 128.2 (d, *J* = 3.7 Hz), 126.6 (d, *J* = 18.0 Hz), 124.5, 123.9, 118.2, 116.4, 116.3 (d, *J* = 4.5 Hz), 116.1, 104.4, 99.4, 50.9, 14.7 (d, *J* = 3.3 Hz). HRMS (ESI): *m*/*z* calcd for C_24_H_21_FN_3_O_3_ [M + H]^+^ 418.1561, found 418.1568.

*(E)-3-(2,4-Difluorophenyl)-2-(4-(2-oxo-2H-Chromen-4-yl)Piperazine-1-Carbonyl)Acrylonitrile* (**5m**). Yellow solid (97 mg, 47.3% yield). Mp. 208–210 °C. HPLC purity: 97.5%, t_R_ = 12.36 min. ^1^H NMR (600 MHz, CDCl_3_) *δ* 8.33 (m, 1H), 8.01 (s, 1H), 7.60 (dd, *J* = 8.0, 1.3 Hz, 1H), 7.54 (ddd, *J* = 8.2, 7.6, 1.3 Hz, 1H), 7.37 (dd, *J* = 8.2, 1.0 Hz, 1H), 7.29 (ddd, *J* = 8.0, 7.6, 1.0 Hz, 1H), 7.04 (m, 1H), 6.94 (ddd, *J* = 10.7, 8.5, 2.5 Hz, 1H), 5.79 (s, 1H), 3.95 (t, *J* = 4.9 Hz, 4H), 3.35 (t, *J* = 4.9 Hz, 4H). ^13^C NMR (150 MHz, CDCl_3_) *δ* 162.8, 162.1, 160.7, 154.4, 143.6 (dd, *J* = 1.19, 1.21 Hz), 132.1, 130.2 (d, *J* = 2.3 Hz), 130.2 (d, *J* = 2.1 Hz), 124.4, 123.9, 118.2, 116.1, 115.8, 112.9 (dd, *J* = 3.0, 3.3 Hz), 107.0, 106.1 (d, *J* = 25.4 Hz), 106.1, 104.9 (d, *J* = 25.4 Hz), 99.52, 50.8. HRMS (ESI): *m*/*z* calcd for C_23_H_18_F_2_N_3_O_3_ [M + H]^+^ 422.1311, found 422.1308.

*(E)-3-(3-Chlorophenyl)-2-(4-(2-oxo-2H-Chromen-4-yl)Piperazine-1-Carbonyl)Acrylonitrile* (**5n**). Yellow solid (47 mg, 36.2% yield). Mp. 180–182 °C. HPLC purity: 95.9%, t_R_ = 11.84 min. ^1^H NMR (600 MHz, CDCl_3_) *δ* 7.86–7.81 (m, 2H), 7.77 (s, 1H), 7.60 (dd, *J* = 8.0, 1.3 Hz, 1H), 7.53 (ddd, *J* = 8.3, 7.4, 1.3 Hz, 1H), 7.50 (ddd, *J* = 8.0, 1.8, 1.0 Hz, 1H), 7.44 (dd, *J* = 8.0, 8.0 Hz, 1H), 7.36 (dd, *J* = 8.3, 0.9 Hz, 1H), 7.29 (ddd, *J* = 8.0, 7.4, 1.0 Hz, 1H), 5.78 (s, 1H), 3.95 (t, *J* = 5.0 Hz, 4H), 3.34 (t, *J* = 4.9 Hz, 4H). ^13^C NMR (150 MHz, CDCl_3_) *δ* 162.8, 162.1, 160.7, 154.3, 150.8, 135.4, 133.7, 132.6, 132.1, 130.7, 130.1, 128.1, 124.4, 123.9, 118.1, 116.0, 115.7, 107.2, 99.4, 50.8. HRMS (ESI): *m*/*z* calcd for C_23_H_19_ClN_3_O_3_ [M + H]^+^ 420.1109, found 420.1104.

*(E)-3-(4-Chlorophenyl)-2-(4-(2-oxo-2H-Chromen-4-yl)Piperazine-1-Carbonyl)Acrylonitrile* (**5o**). White solid (30 mg, 23.1% yield). Mp. 200–202 °C. HPLC purity: 95.7%, t_R_ = 14.05 min. ^1^H NMR (600 MHz, CDCl_3_) *δ* 7.86 (d, *J* = 8.6 Hz, 2H), 7.81 (s, 1H), 7.60 (dd, *J* = 8.0, 1.3 Hz, 1H), 7.54 (ddd, *J* = 8.4, 7.8, 1.3 Hz, 1H), 7.48 (d, *J* = 8.6 Hz, 2H), 7.36 (dd, *J* = 8.4, 1.0 Hz, 1H), 7.29 (ddd, *J* = 8.0, 7.8, 1.0 Hz, 1H), 5.78 (s, 1H), 3.95 (t, *J* = 4.9 Hz, 4H), 3.34 (t, *J* = 4.9 Hz, 4H). ^13^C NMR (150 MHz, CDCl_3_) *δ* 163.1, 162.1, 160.7, 154.4, 152.3, 139.1, 132.1, 131.6, 130.5, 129.8, 124.4, 123.9, 118.2, 116.1, 116.0, 105.9, 99.4, 50.9. HRMS (ESI): *m*/*z* calcd for C_23_H_19_ClN_3_O_3_ [M + H]^+^ 420.1109, found 420.1096.

*(E)-3-(3,4-Dichlorophenyl)-2-(4-(2-oxo-2H-Chromen-4-yl)Piperazine-1-Carbonyl)Acrylonitrile* (**5p**). White solid (43 mg, 30.6% yield). Mp. 198–200 °C. HPLC purity: 97.7%, t_R_ = 13.96 min. ^1^H NMR (600 MHz, CDCl_3_) *δ* 7.95 (d, *J* = 2.0 Hz, 1H), 7.80 (dd, *J* = 8.4, 2.0 Hz, 1H), 7.75 (s, 1H), 7.60 (dd, *J* = 8.0, 1.3 Hz, 1H), 7.58 (d, *J* = 8.4 Hz, 1H), 7.54 (ddd, *J* = 8.3, 7.6, 1.3 Hz, 1H), 7.37 (dd, *J* = 8.3, 0.9 Hz, 1H), 7.29 (ddd, *J* = 8.0, 7.6, 0.9 Hz, 1H), 5.78 (s, 1H), 3.95 (t, *J* = 4.9 Hz, 4H), 3.33 (t, *J* = 4.9 Hz, 4H). ^13^C NMR (150 MHz, CDCl_3_) *δ* 162.6, 162.1, 160.6, 154.4, 150.7, 137.1, 133.9, 132.1, 132.0, 131.8, 131.5, 128.8, 124.4, 123.9, 118.2, 116.1, 115.5, 107.4, 99.5, 50.8. HRMS (ESI): *m*/*z* calcd for C_23_H_18_Cl_2_N_3_O_3_ [M + H]^+^ 454.0720, found 454.0724.

*(E)-3-(3-Bromophenyl)-2-(4-(2-oxo-2H-Chromen-4-yl)Piperazine-1-Carbonyl)Acrylonitrile* (**5q**). White solid (59 mg, 41.1% yield). Mp. 192–194 °C. HPLC purity: 96.3%, t_R_ = 13.76 min. ^1^H NMR (600 MHz, CDCl_3_) *δ* 7.98 (s, 1H), 7.89 (d, *J* = 8.0 Hz, 1H), 7.75 (s, 1H), 7.66 (d, *J* = 8.0 Hz, 1H), 7.60 (dd, *J* = 8.0, 1.1 Hz, 1H), 7.53 (ddd, *J* = 8.6, 7.4, 1.1 Hz, 1H),7.38 (m, 2H), 7.29 (dd, *J* = 8.0, 7.4 Hz, 1H), 5.78 (s, 1H), 3.95 (t, *J* = 4.9 Hz, 4H), 3.34 (t, *J* = 4.9 Hz, 4H). ^13^C NMR (150 MHz, CDCl_3_) *δ* 162.8, 162.1, 160.7, 154.4, 150.7, 135.6, 133.9, 133.1, 132.1, 130.9, 128.4, 124.4, 123.9, 123.4, 118.2, 116.1, 115.6, 107.2, 99.5, 50.9. HRMS (ESI): *m*/*z* calcd for C_23_H_19_BrN_3_O_3_ [M + H]^+^ 464.0604, 466.0584, found 464.0612, 466.0596.

*(E)-3-(4-Bromophenyl)-2-(4-(2-oxo-2H-Chromen-4-yl)Piperazine-1-Carbonyl)Acrylonitrile* (**5r**). Yellow solid (55 mg, 38.3% yield). Mp. 218-220 °C. HPLC purity: 96.0%, t_R_ = 14.41 min. ^1^H NMR (600 MHz, CDCl_3_) *δ* 7.79 (d, *J* = 8.6 Hz, 2H), 7.77 (s, 1H), 7.64 (d, *J* = 8.6 Hz, 2H), 7.60 (dd, *J* = 8.0, 1.2 Hz, 1H), 7.54 (ddd, *J* = 8.3, 7.8, 1.2 Hz, 1H), 7.37 (d, *J* = 8.3 Hz, 1H), 7.29 (dd, *J* = 8.0, 7.8 Hz, 1H), 5.78 (s, 1H), 3.95 (t, *J* = 4.9 Hz, 4H), 3.34 (t, *J* = 4.9 Hz, 4H). ^13^C NMR (150 MHz, CDCl_3_) *δ* 163.1, 162.1, 160.7, 154.4, 152.3, 132.8, 132.1, 131.6, 130.9, 127.7, 124.4, 123.9, 118.2, 116.1, 116.0, 106.0, 99.5, 50.9. HRMS (ESI): *m*/*z* calcd for C_23_H_19_BrN_3_O_3_ [M + H]^+^ 464.0604, 466.0584, found 464.0610, 466.0596.

*(E)-2-(4-(2-Oxo-2H-Chromen-4-yl)Piperazine-1-Carbonyl)-3-(3-(Trifluoromethyl)Phenyl)Acrylonitrile* (**5s**). Yellow solid (18 mg, 12.8% yield). Mp. 88–90 °C. HPLC purity: 96.1%, t_R_ = 13.56 min. ^1^H NMR (600 MHz, CDCl_3_) *δ* 8.17 (d, *J* = 7.9 Hz, 1H), 8.07 (s, 1H), 7.87 (s, 1H), 7.79 (d, *J* = 7.8 Hz, 1H), 7.66 (dd, *J* = 7.8, 7.8 Hz, 1H), 7.61 (dd, *J* = 8.0, 1.4 Hz, 1H), 7.54 (ddd, *J* = 8.3, 7.6, 1.4 Hz, 1H), 7.37 (dd, *J* = 8.3, 0.9 Hz, 1H), 7.29 (ddd, *J* = 8.0, 7.6, 0.9 Hz, 1H), 5.79 (s, 1H), 3.96 (t, *J* = 4.9 Hz, 4H), 3.35 (t, *J* = 4.9 Hz, 4H). ^13^C NMR (150 MHz, CDCl_3_) *δ* 162.6, 162.1, 160.7, 154.4, 150.6, 132.7, 132.6, 132.1 (d, *J* = 5.9 Hz), 131.9, 130.1 (d, *J* = 9.7 Hz), 129.9, 129.1 (d, *J* = 3.6 Hz), 127.3 (d, *J* = 3.9 Hz), 124.4, 123.9, 118.2, 116.1, 115.6, 107.9, 99.5, 50.9. ^13^C NMR (150 MHz, CDCl_3_) *δ* 162.6, 162.1, 160.7, 154.4, 151.6, 132.7, 132.6, 132.1, 132.1, 131.9, 130.2, 130.1, 128.1 (q, *J* = 270.6 Hz), 124.4, 123.9, 118.2, 116.1, 115.6, 107.9, 99.5, 50.9. HRMS (ESI): *m*/*z* calcd for C_23_H_19_F_3_N_3_O_3_ [M + H]^+^ 454.1373, found 454.1372.

*(E)-3-(2-Cyano-3-oxo-3-(4-(2-oxo-2H-Chromen-4-yl)Piperazin-1-yl)Prop-1-Enyl)Benzonitrile* (**5t**). Yellow solid (13 mg, 10.2% yield). Mp. 236–238 °C. HPLC purity: 96.1%, t_R_ = 11.95 min. ^1^H NMR (600 MHz, CDCl_3_) *δ* 8.19 (d, *J* = 8.0 Hz, 1H), 8.09 (s, 1H), 7.83 (s, 1H), 7.81 (d, *J* = 7.8 Hz, 1H), 7.66 (dd, *J* = 8.0, 7.8 Hz, 1H), 7.60 (dd, *J* = 8.0, 1.3 Hz, 1H), 7.55 (ddd, *J* = 8.3, 7.6, 1.3 Hz, 1H), 7.37 (dd, *J* = 8.3, 1.0 Hz, 1H), 7.30 (ddd, *J* = 8.0, 7.6, 1.0 Hz, 1H), 5.79 (s, 1H), 3.96 (t, *J* = 5.0 Hz, 4H), 3.35 (t, *J* = 5.0 Hz, 4H). ^13^C NMR (150 MHz, CDCl_3_) *δ* 162.3, 162.1, 160.7, 154.4, 150.7, 135.4, 133.6, 133.5, 133.2, 132.1, 130.4, 124.4, 124.0, 118.2, 117.6, 116.0, 115.3, 114.1, 108.7, 99.6, 50.8. HRMS (ESI): *m*/*z* calcd for C_24_H_19_N_4_O_3_ [M + H]^+^ 411.1452, found 411.1454.

*(E)-3-(3-Nitrophenyl)-2-(4-(2-oxo-2H-Chromen-4-yl)Piperazine-1-Carbonyl)Acrylonitrile* (**5u**). White solid (25 mg, 18.7% yield). Mp. 238–240 °C. HPLC purity: 95.7%, t_R_ = 13.23 min. ^1^H NMR (600 MHz, CDCl_3_) *δ* 8.67 (s, 1H), 8.39 (d, *J* = 8.2 Hz, 1H), 8.30 (d, *J* = 7.8 Hz, 1H), 7.91 (s, 1H), 7.73 (dd, *J* = 8.2, 7.8 Hz, 1H), 7.61 (dd, *J* = 8.0, 1.0 Hz, 1H), 7.55 (ddd, *J* = 8.3, 7.4, 1.2 Hz, 1H), 7.38 (d, *J* = 8.1 Hz, 1H), 7.30 (dd, *J* = 7.8, 7.4 Hz, 1H), 5.80 (s, 1H), 3.97 (t, *J* = 4.7 Hz, 4H), 3.34 (t, *J* = 4.7 Hz, 4H). ^13^C NMR (150 MHz, CDCl_3_) *δ* 162.2, 162.1, 160.6, 154.4, 150.6, 148.7, 134.8, 133.5, 132.1, 130.7, 126.8, 125.2, 124.4, 123.9, 118.2, 116.0, 115.3, 109.2, 99.6, 50.8. HRMS (ESI): *m*/*z* calcd for C_23_H_18_N_4_NaO_5_ [M + Na]^+^ 453.1196, found 453.1197.

#### 3.1.4. General Procedures for Synthesis of **6a** and **6b**

To a solution of corresponding acid (0.5 mmol) in dry CH_2_Cl_2_ (3 mL) was added HATU (190 mg, 0.5 mmol) and *N*,*N*-diisopropylethylamine (97 mg, 0.75 mmol) at room temperature. After 0.5 h, compound **3** (115 mg, 0.5 mmol) was added, and the reaction mixture was stirred at room temperature for 4 h. The volatiles were removed under reduced pressure, and the residue was partitioned between EtOAc and H_2_O. The organic layers were combined and dried over MgSO_4_. The residue was purified by flash column chromatography to afford the desired products **6a** and **6b**, respectively.

*(E)-4-(4-(3-(3,4-Dichlorophenyl)Acryloyl)Piperazin-1-yl)-2H-Chromen-2-One* (**6a**). White solid (45 mg, 21.0% yield). Mp. 238–240 °C. HPLC purity: 98.8%, t_R_ = 15.05 min. ^1^H NMR (600 MHz, CDCl_3_) *δ* 7.65–7.61 (m, 3H), 7.54 (ddd, *J* = 8.4, 7.4, 1.5 Hz, 1H), 7.47 (d, *J* = 8.3 Hz, 1H), 7.37 (dd, *J* = 8.4, 1.0 Hz, 1H), 7.35 (dd, *J* = 8.3, 2.0 Hz, 1H), 7.31–7.28 (m, 1H), 6.90 (d, *J* = 15.4 Hz, 1H), 5.77 (s, 1H), 3.99 (brs, 2H), 3.92 (brs, 2H), 3.31 (t, *J* = 4.9 Hz, 4H). ^13^C NMR (150 MHz, CDCl_3_) *δ* 165.2, 162.2, 160.9, 154.4, 141.3, 135.1, 134.0, 133.4, 132.0, 131.0, 129.3, 127.3, 124.5, 123.86, 118.24, 118.16, 116.16, 99.16, 51.16. HRMS (ESI): *m*/*z* calcd for C_22_H_19_Cl_2_N_2_O_3_ [M + H]^+^ 429.0773, found 429.0773.

*(E)-4-(4-(3-(3-Bromophenyl)Acryloyl)Piperazin-1-yl)-2H-Chromen-2-One* (**6b**). White solid (48 mg, 21.9% yield). Mp. 172–174 °C. HPLC purity: 98.6%, t_R_ = 14.28 min. ^1^H NMR (600 MHz, CDCl_3_) *δ* 7.70 (t, *J* = 1.5 Hz, 1H), 7.66 (d, *J* = 15.4 Hz, 1H), 7.63 (dd, *J* = 8.0, 1.4 Hz, 1H), 7.54 (ddd, *J* = 8.3, 7.6, 1.4 Hz, 1H), 7.51–7.48 (m, 1H), 7.44 (d, *J* = 7.7 Hz, 1H), 7.37 (dd, *J* = 8.3, 1.0 Hz, 1H), 7.29 (m, 2H), 6.91 (d, *J* = 15.4 Hz, 1H), 5.77 (s, 1H), 3.99 (brs, 2H), 3.93 (brs, 2H), 3.31 (t, *J* = 4.9 Hz, 4H). ^13^C NMR (150 MHz, CDCl_3_) *δ* 165.4, 162.2, 160.9, 154.4, 142.3, 137.2, 132.8, 132.0, 130.6, 130.3, 127.0, 124.6, 123.8, 123.2, 118.1, 117.9, 116.2, 99.1, 51.2. HRMS (ESI): *m*/*z* calcd for C_22_H_20_BrN_2_O_3_ [M + H]^+^ 439.0657, found 439.0634.

### 3.2. Biological Evaluation Methods

#### 3.2.1. Cell Line and Culture Condition

All of the cell lines were purchased from Cell Bank of Chinese Academy of Sciences, Shanghai Branch (Shanghai, China). A549, H157, HepG2, MCF7, MG63, and U2OS cells were cultured in specified medium supplemented with 10% fetal bovine serum (Gibco), 100 IU/mL penicillin and 100 μg/mL streptomycin (both from Thermo Fisher Scientific, Inc., Waltham, MA, USA) in a humidified atmosphere containing 5% CO_2_ at 37 °C.

#### 3.2.2. Cell Viability Assay

The cells were cultured in 96-well plates, and each well was seeded with 1 × 10^4^ cells. After incubation for 16 h, the medium was removed and replaced with 100 μL medium containing the indicated concentrations of compounds and further incubated for 48 h. Then, the viability of cells was measured by the MTT method [39]. Briefly, 0.02 mL of MTT solution (5 mg/mL in PBS) was added to each well and then incubated for 4 h at 37 °C. In this step, 100 μL of dimethyl sulfoxide (DMSO) was added to dissolve the formazan crystals. The optical density (OD) was measured at an absorbance wavelength of 490 nm while using a Microplate Reader (Tecan, Switzerland).

#### 3.2.3. Microscopy and Photography

MG63 cells were seeded into 24-well plates and treated with different dilutions of **5q**. Cells were observed using an inverted microscope (Nikon, Japan) after incubation for 24 h.

#### 3.2.4. Electron Microscope Analysis

MG63 cells were incubated with 5 μM or 0 μM of **5q** for 24 h and were then fixed with 4% glutaraldehyde for 2 h at room temperature. Cells were subsequently carried out in 1% OsO_4_ in 0.15 M phosphate buffer for 1 h, followed by a rapid wash in the same buffer. The cells were dehydrated in increasing grades of ethanol (50%, 70%, 95%, 100%, 15–20 min each) and then critical point dyring. Observation under conventional scanning electron microscope (JSM-4800, JEOL, Akishima, Japan) was generally performed.

#### 3.2.5. DNA Fragmentation Analysis with DAPI Staining

Cells (1 × 10^5^ cells/well) were seeded in 6-well plates. After attachment, cells were treated with various concentrations of **5q** (0–5 μM) for 24 h. Then cells were fixed with 4% polyoxymethylene (PFA, Sigma-Aldrich, St. Louis, MO, USA) for 30 min, washed twice with PBS, and then incubated with 10 μg/mL 4,6-diamidino-2-phenylindole (DAPI, Sigma-Aldrich) for 10 min at room temperature. After washing with PBS two times, the images were photographed by an inverted fluorescence microscope (Leica, Buffalo Grove, IL, USA).

#### 3.2.6. Apoptosis Analysis

Cell apoptosis assays were performed with an Annexin V-FITC apoptosis detection kit (BD Biosciences, Franklin Lakes, NJ, USA), according to the manufacturer’s protocol. MG63 cells were treated with different doses of **5q** for 24 h. Cells were washed twice with PBS and harvested by re-suspension in 100 μL of 1 × binding buffer, followed by incubation with 5 μL each of Annexin V fluorescein isothiocyanate and propidium iodide for 15 min at room temperature in the dark. Finally, adding 400 μL of 1 × binding buffer to the tube and cell apoptosis was measured by flow cytometer (FACS Calibur; Becton-Dickinson, Rutherford, NJ, USA). The data were analyzed using flowjo 7.0.

#### 3.2.7. Reverse Transcription Quantitative Real-Time PCR

Total RNA of MG63 cells treated with or without **5q** for 24 h was extracted using TRIzol reagent (Invitrogen, Waltham, MA, USA), following the manufacturer’s protocol. The quantity of RNA was determined using the ultramicro spectrophotometer (NanoDrop 2000, Thermo Fisher Scientific, Waltham, MA, USA). Complementary DNA was generated in a 20 μL reaction with one microgram of total RNA using a RT reaction kit (Promega, Madison, WI, USA), according to the manufacturer’s introduction. Real-time PCR was performed while using a CRX Connect Real-Time system (Bio-Rad, Hercules, CA, USA) and using SYBR Premix Ex Taq™ (TaKaRa, Ohtsu, Shiga, Japan) as a DNA-specific fluorescent dye. PCR was carried out for 40 cycles at 95 °C for 10 s and 60 °C for 30 s. Gene expression levels were calculated relative to the housekeeping β-actin and all of the reactions were repeated at least three times. The primer sequences used were listed in Table 3.

#### 3.2.8. Western Blotting Analysis

MG63 cells were treated with different doses of **5q** for 24 h. The cells were lysed in 1 × RIPA buffer containing protease inhibitor cocktails (Roche, South San Francisco, CA, USA). Protein concentrations were determined while using BCA protein assay kit (Beyotime Institute of Biotechnology, Haimen, Jiansu, China. Equivalent quantities of total protein were electrophoretically separated on a 10% sodium dodecyl sulfate-polyacrylamide gel (SDS-PAGE). The total protein were then transferred onto polyvinylidene fluoride (PVDF) membranes and blocked for 60 min with 3% bovine serum albumin (BSA) solution prepared in PBS at room temperature. A 1:1000 dilution of the primary antibodies (Bax, Bcl-2, caspase-3, caspase-8, caspase-9, and β-actin) were incubated overnight at 4 °C. The membranes were washed three times with TBST buffer (0.05% Tris-buffered saline and Tween 20). The appropriate peroxidase-conjugated secondary antibody was incubated for 2 h at room temperature. Subsequently, the results were visualized with enhanced chemiluminescence reagent (Merck Millipore, Darmstadt, Germany) while using imaging system (Chemi-Doc XRS imager, Bio-Rad, Hercules, CA, USA 

### 3.3. Statistical Analyses

All of the experimental values were expressed as mean ± standard deviation (SD) of at least three independent experiments. SPSS 18.0 statistical software package (SPSS Inc, Chicago, IL, USA) was used to perform all of the statistical analysis. The statistical significance of the differences between groups was evaluated by Student’s *t*-test. *p* < 0.05 was considered to indicate a statistically significant difference. * *p* < 0.05, ** *p* < 0.01, and *** *p* < 0.001, respectively.

## 4. Conclusions

In summary, a series of coumarin/2-cyanoacryloyl hybrids were prepared and evaluated for their in vitro anticancer potential against a panel of human cancer cell lines, including A549, H157, HepG2, MCF7, MG63, and U2OS. From the in vitro screening results, compounds **5p** and **5q** were efficacious in suppressing the growth of all the six tested human cancer cell lines. The preliminary SAR study suggested that the halogen atom in benzene ring and cyano group in α,β-unsaturated ketone were vital to increase their activity. Data obtained showed that the inhibition of growth on osteosarcoma MG63 cells by **5q** resulted from the inducement of apoptosis in vitro. The effects of **5q**-induced apoptosis in MG63 cells were associated with down-regulation of the anti-apoptotic protein Bcl-2, upregulation of pro-apoptotic protein Bax, and induced activation of caspase-3, 8, and 9. Overall, compound **5q** could be established as a new potential anticancer candidate worth of detailed and extensive investigation.

## Figures and Tables

**Table 1:** Antiproliferative activity of **5a**–**5u**, **6a**, and **6b** against A549 cells.

Compound	R	Inhibition Ratio at 20 μM ^a^	IC_50_^b^ (μM)
**3**	-	1.28%	NT ^c^
**4**	-	2.31%	NT
**5a**	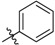	39.18%	NT
**5b**	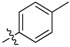	34.29%	NT
**5c**	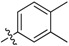	26.67%	NT
**5d**	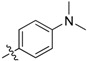	27.60%	NT
**5e**	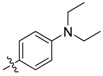	13.03%	NT
**5f**	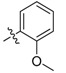	24.62%	NT
**5g**	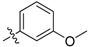	15.83%	NT
**5h**	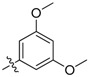	29.77%	NT
**5i**	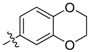	29.19%	NT
**5j**	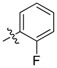	94.06%	13.06 ± 0.76
**5k**	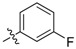	74.79%	18.92 ± 0.51
**5l**	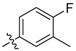	93.28%	10.04 ± 0.25
**5m**	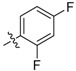	32.09%	NT
**5n**	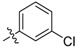	95.65%	8.37 ± 0.75
**5o**	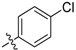	95.03%	12.10 ± 0.33
**5p**	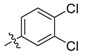	97.29%	6.26 ± 0.07
**5q**	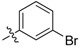	97.96%	7.28 ± 0.57
**5r**	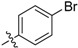	67.57%	18.91 ± 1.21
**5s**	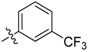	94.02%	16.44 ± 0.70
**5t**	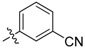	40.68%	NT
**5u**	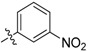	9.01%	NT
**6a**	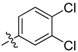	45.92%	NT
**6b**	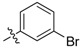	25.83%	NT
Doxorubicin		98.7%	1.12 ± 0.02

^a^ Inhibition ratio less than 50% at 20 μM was considered to be inactive. ^b^ IC_50_ value was expressed as mean ± SD from three independent experiments. ^c^ NT, Not tested.

**Table 2:** Antiproliferative activity of selected compounds presented as IC_50_^a^ (μM).

Compound	H157	HepG2	MCF7	MG63	U2OS
**5j**	>20 ^b^	12.84 ± 0.33	>20	>20	>20
**5k**	>20	>20	>20	>20	>20
**5l**	18.64 ± 2.13	>20	10.98 ± 0.21	17.32 ± 0.33	>20
**5m**	>20	>20	>20	>20	>20
**5n**	>20	>20	18.75 ± 1.62	16.67 ± 0.34	9.84 ± 0.31
**5o**	8.18 ± 0.50	>20	>20	>20	>20
**5p**	6.09 ± 0.24	5.80 ± 0.26	13.02 ± 0.25	16.51 ± 0.33	19.60 ± 0.39
**5q**	6.73 ± 0.04	13.32 ± 0.83	12.96 ± 0.11	5.06 ± 0.25	18.50 ± 0.46
**5r**	>20	>20	>20	>20	>20
**5s**	15.88 ± 0.79	18.72 ± 1.43	>20	>20	>20
**6a**	>20	>20	>20	>20	>20
**6b**	>20	>20	>20	>20	>20
Doxorubicin	2.88 ± 0.06	1.34 ± 0.02	1.22 ± 0.25	0.010 ± 0.001	0.012 ± 0.001

^a^ IC_50_ value was expressed as mean ± SD from three independent experiments. ^b^ Inhibition ratio less than 50% at 20 μM was considered to be inactive.

**Table 3:** The primers of real-time PCR.

Name	Forward Primer (5′-3′)	Reverse Primer (5′-3′)
Bax	AGCTGAGCGAGTGTCTCAAG	GTCCAATGTCCAGCCCATGA
Bcl-2	GGTGAACTGGGGGAGGATTG	GGCAGGCATGTTGACTTCAC
Caspase-3	TGTGAGGCGGTTGTAGAAGTT	GCTGCATCGACATCTGTACC
Caspase-9	TTCCCAGGTTTTGTTTCCTG	CCTTTCACCGAAACAGCATT
Caspase-8	CATCCAGTCACTTTGCCAGA	GCATCTGTTTCCCCATGTTT
cytochrome C	GAGATGAACAGGGGCTCGAAC	TGCTTCTGCCACATGATAACGAG
β-actin	GCCGCCAGCTCACCAT	TCGATGGGGTACTTCAGGGT

## Supplementary Materials

The following are available online. ^1^H, ^13^C NMR, and HR-MS spectra for **5a**–**5u**, **6a** and **6b**.
