# Supplementary material for: Design, Synthesis, and Antiproliferative Evaluation of Novel Coumarin/2-Cyanoacryloyl Hybrids as Apoptosis Inducing Agents by Activation of Caspase-Dependent Pathway
Source: Molecules. 2018 Aug 7;23(8):1972. doi: 10.3390/molecules23081972 (PMC6222758; doi:10.3390/molecules23081972)

# Supporting Information

$^{1}$H NMR spectrum of **5a**

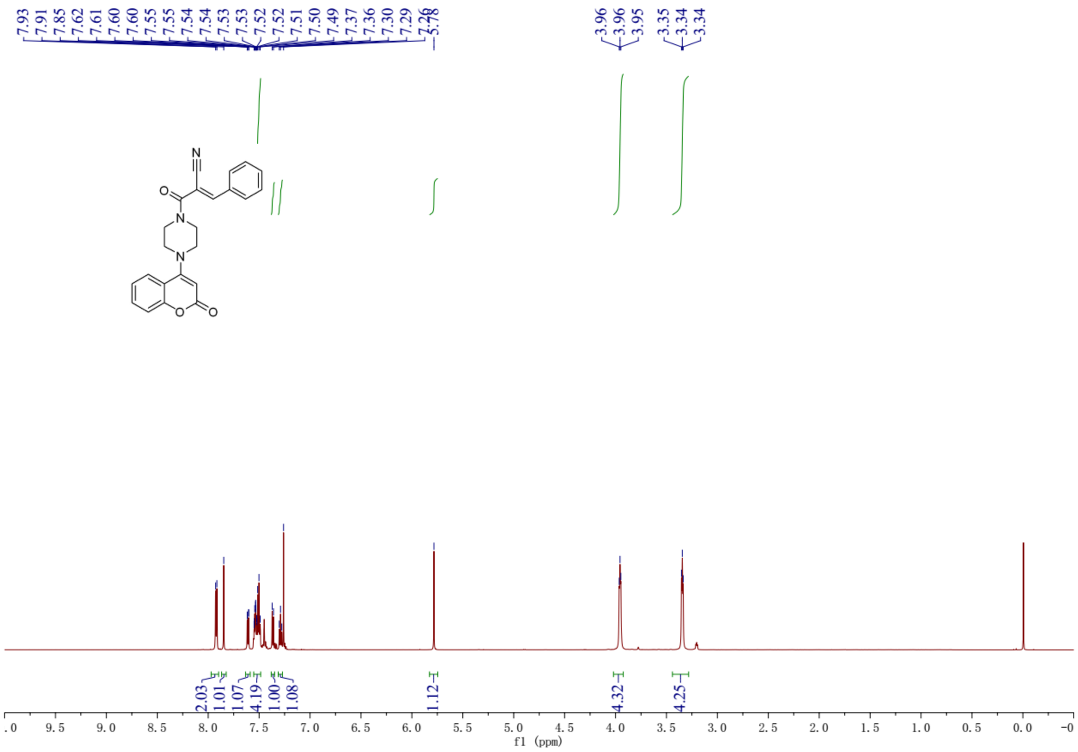

$^{13}$C NMR spectrum of **5a**

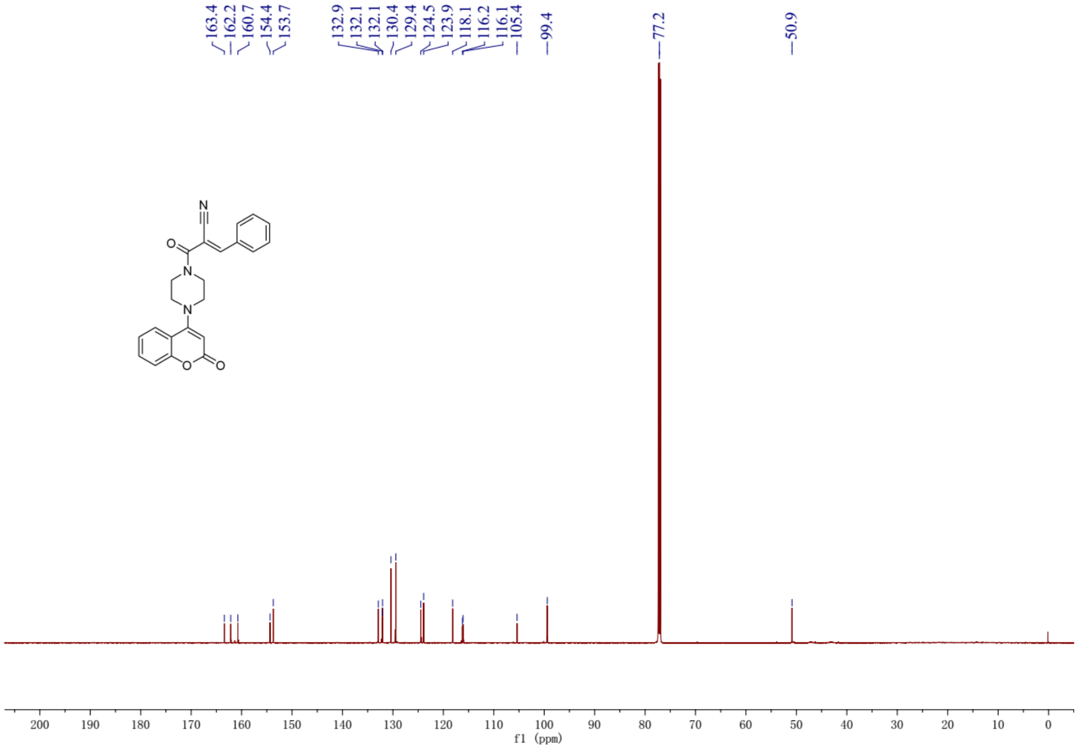

HR-MS (ESI) spectrum of **5a**

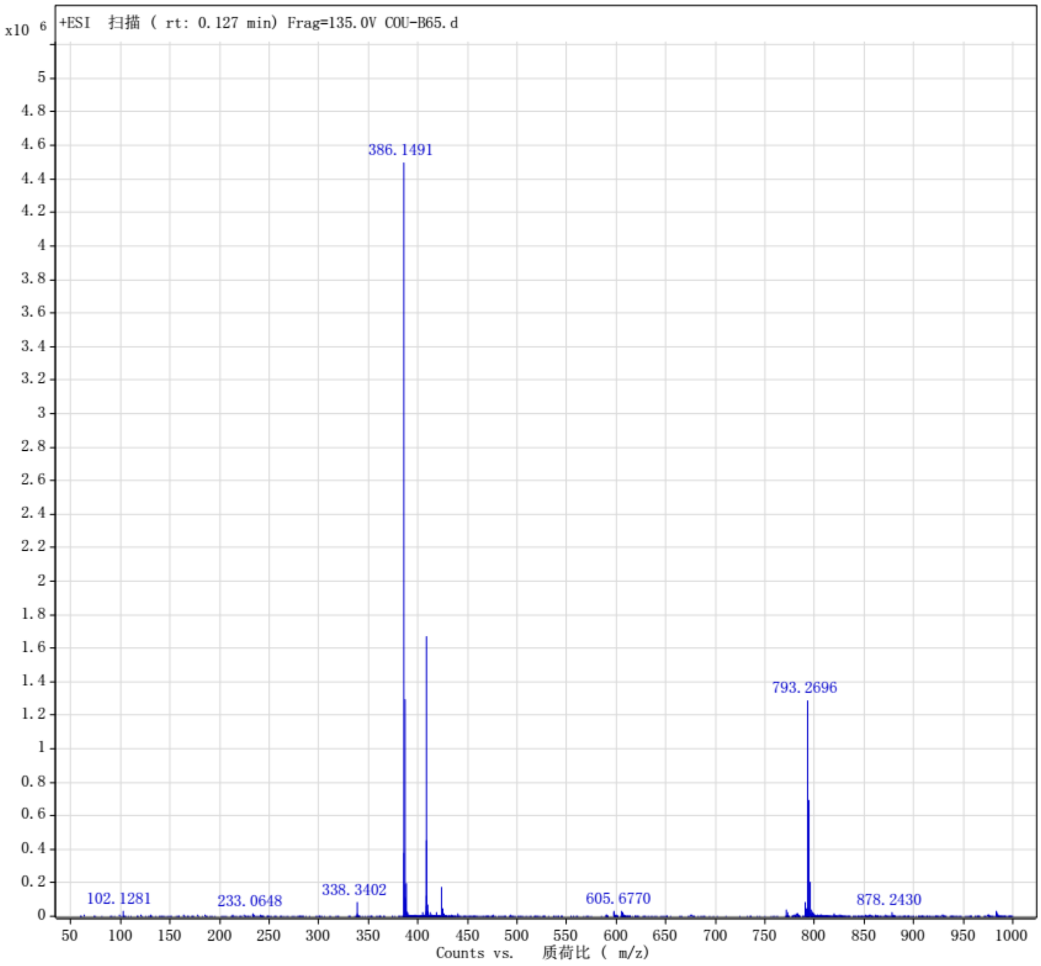

$^{1}$H NMR spectrum of **5b**

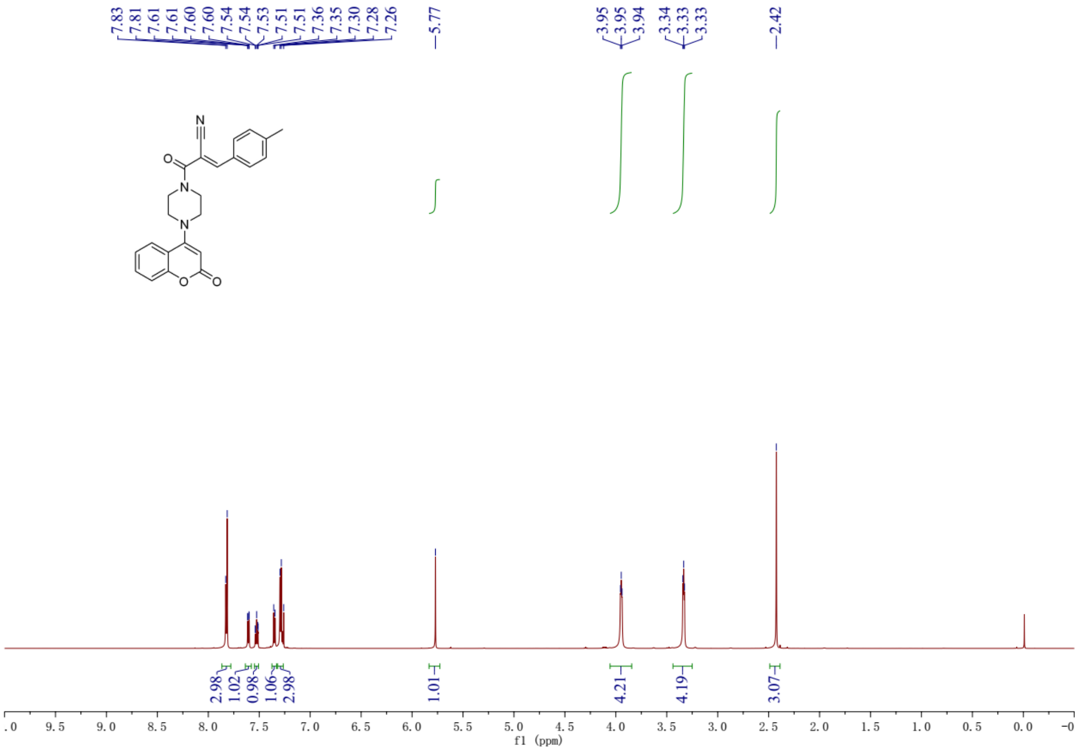


$^{13}$C NMR spectrum of **5b**

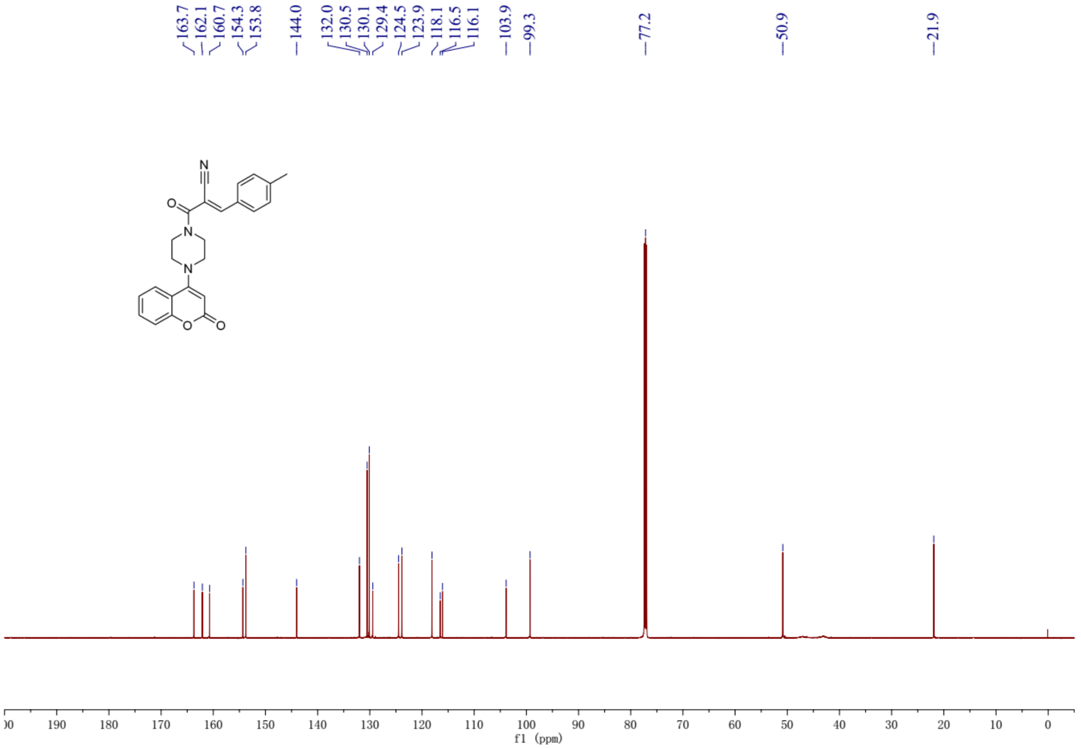

HR-MS (ESI) spectrum of **5b**

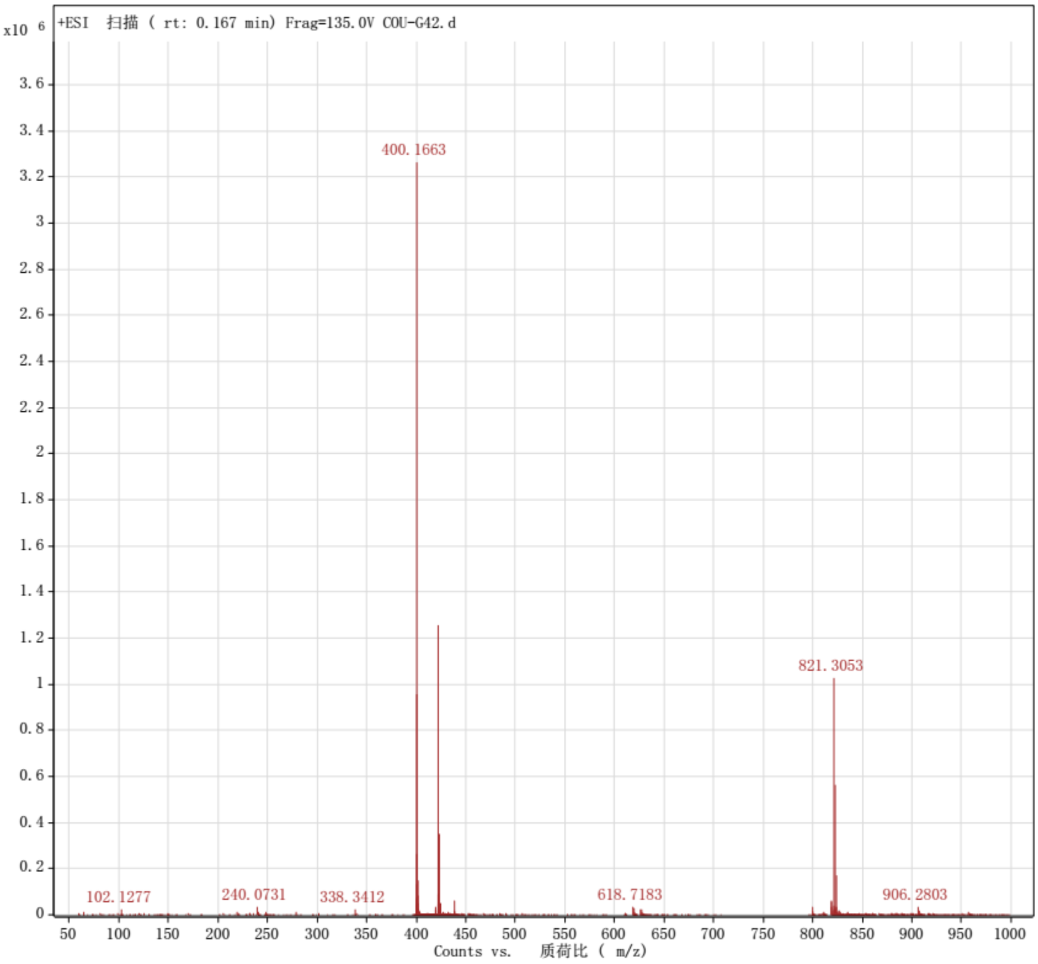

$^{1}$H NMR spectrum of **5c**

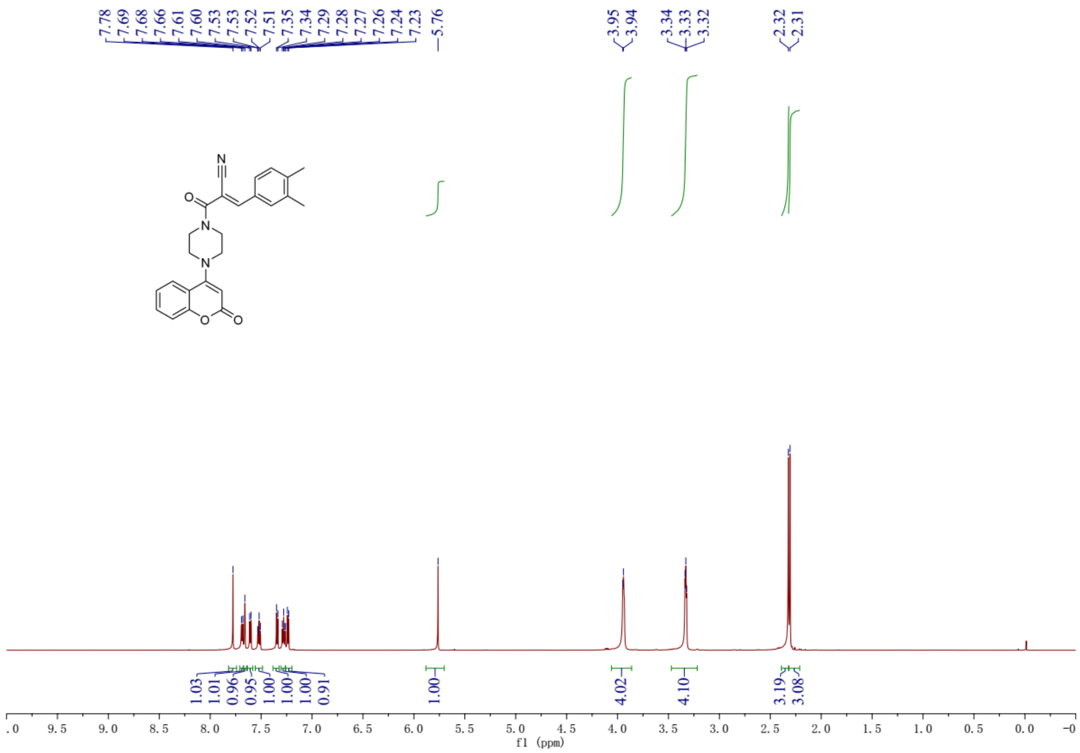


$^{13}$C NMR spectrum of **5c**

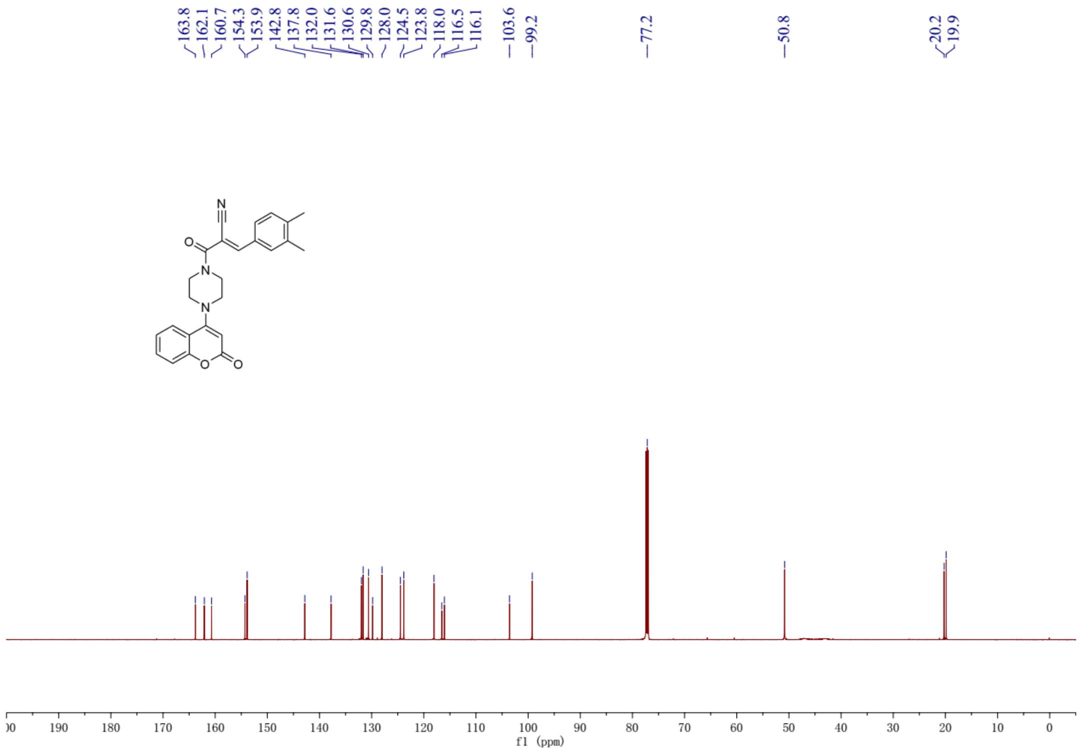

HR-MS (ESI) spectrum of **5c**

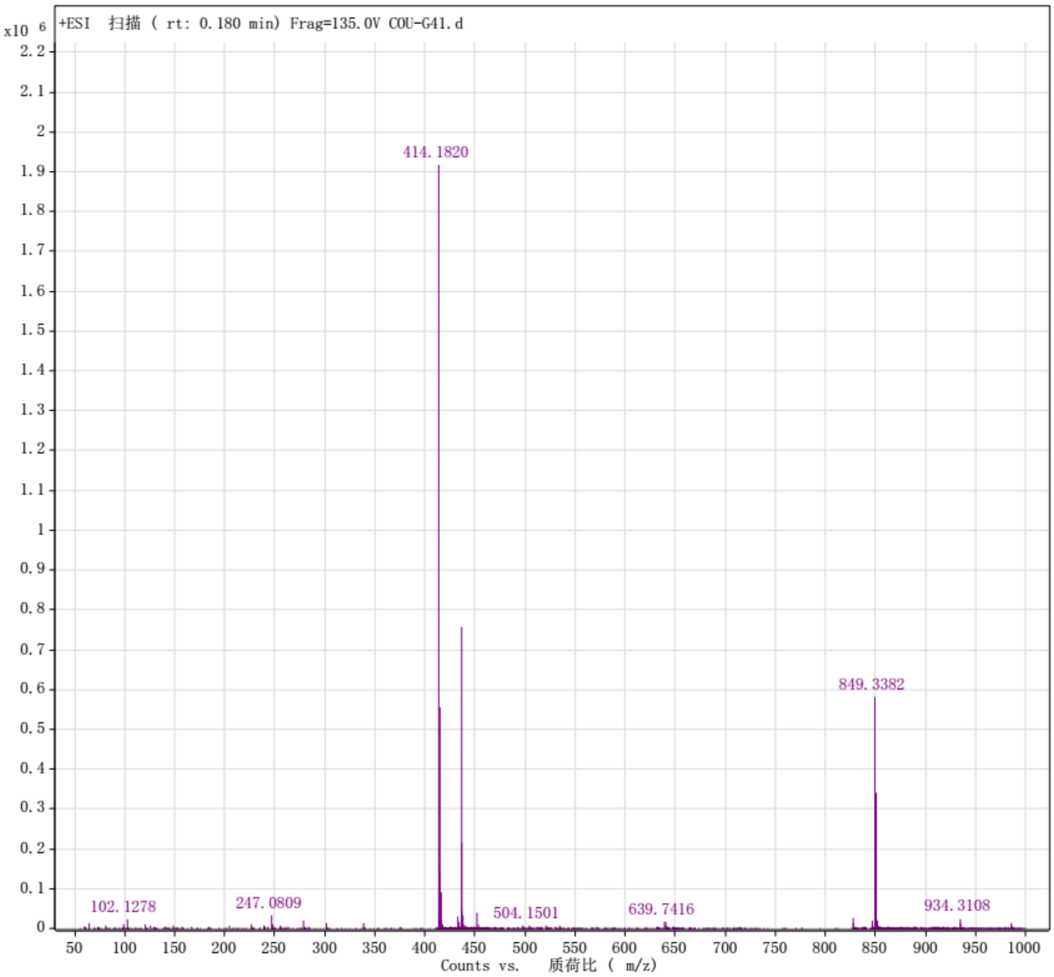

<sup></sup>

$^{1}$H NMR spectrum of **5d**

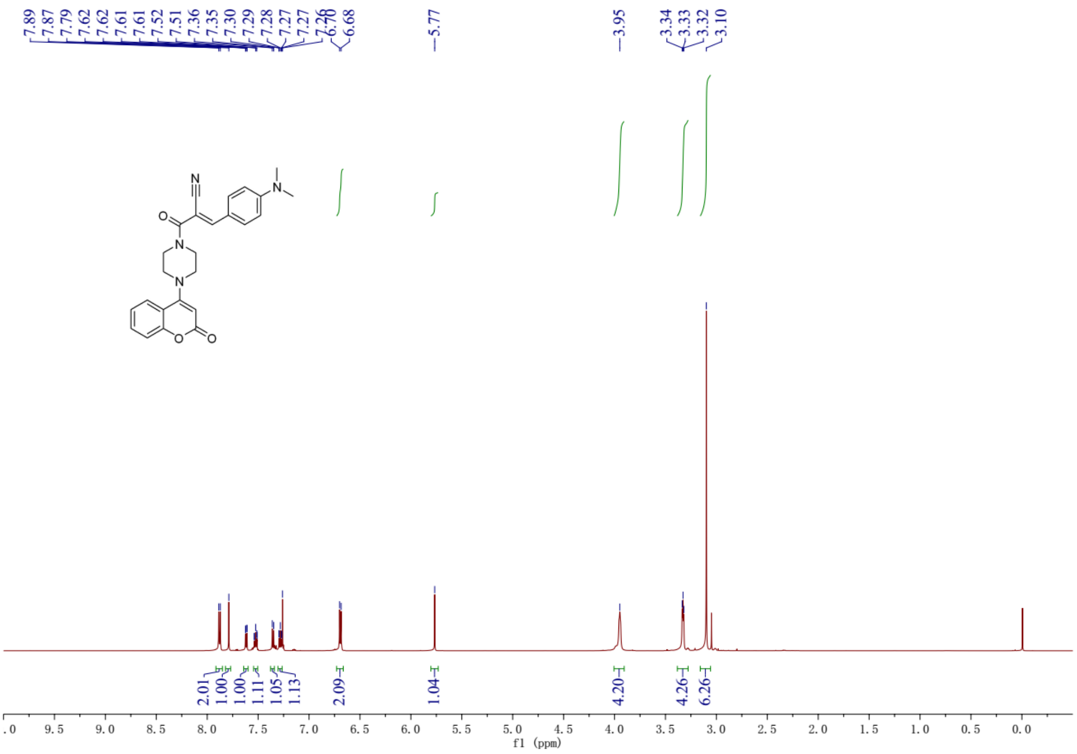

$^{13}$C NMR spectrum of **5d**

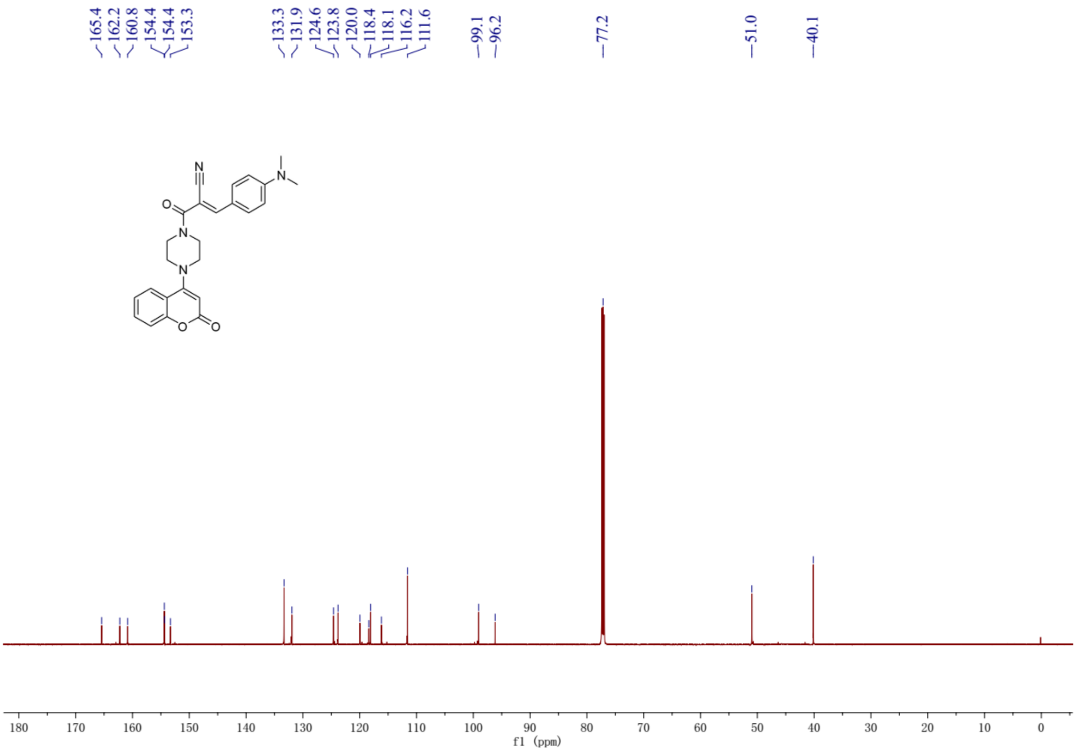

HR-MS (ESI) spectrum of **5d**

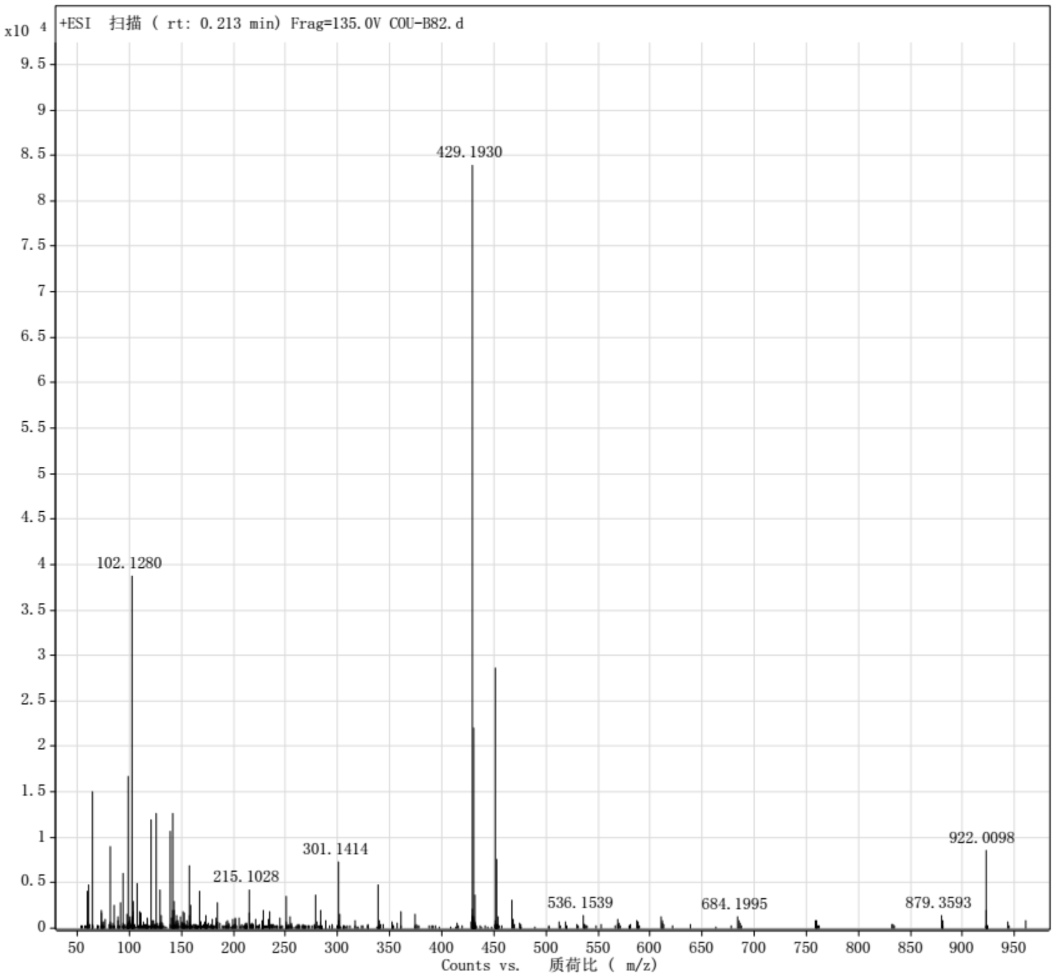

$^{1}H$ NMR spectrum of **5e**

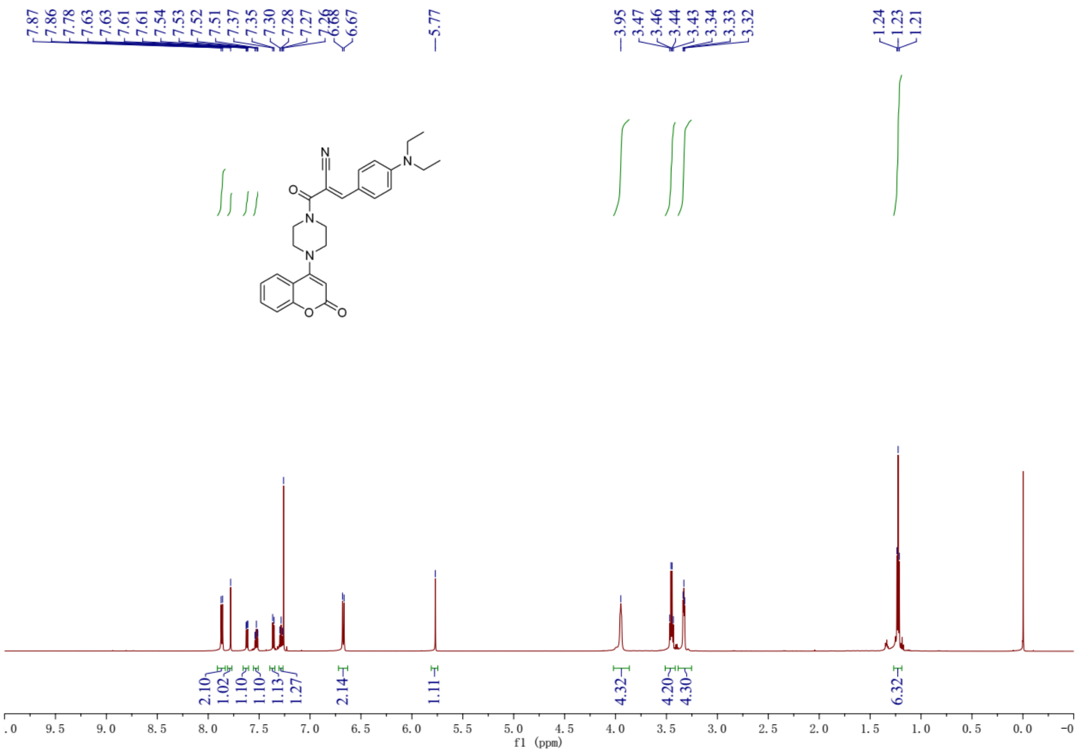

$^{13}C$ NMR spectrum of **5e**

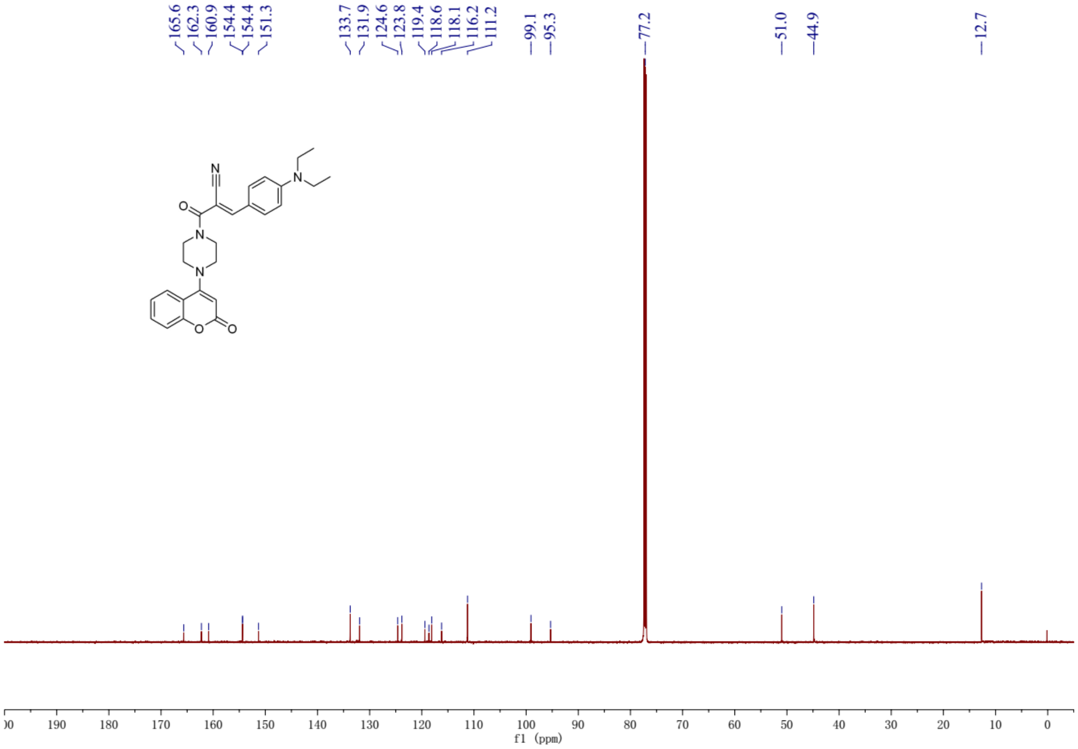

HR-MS (ESI) spectrum of **5e**

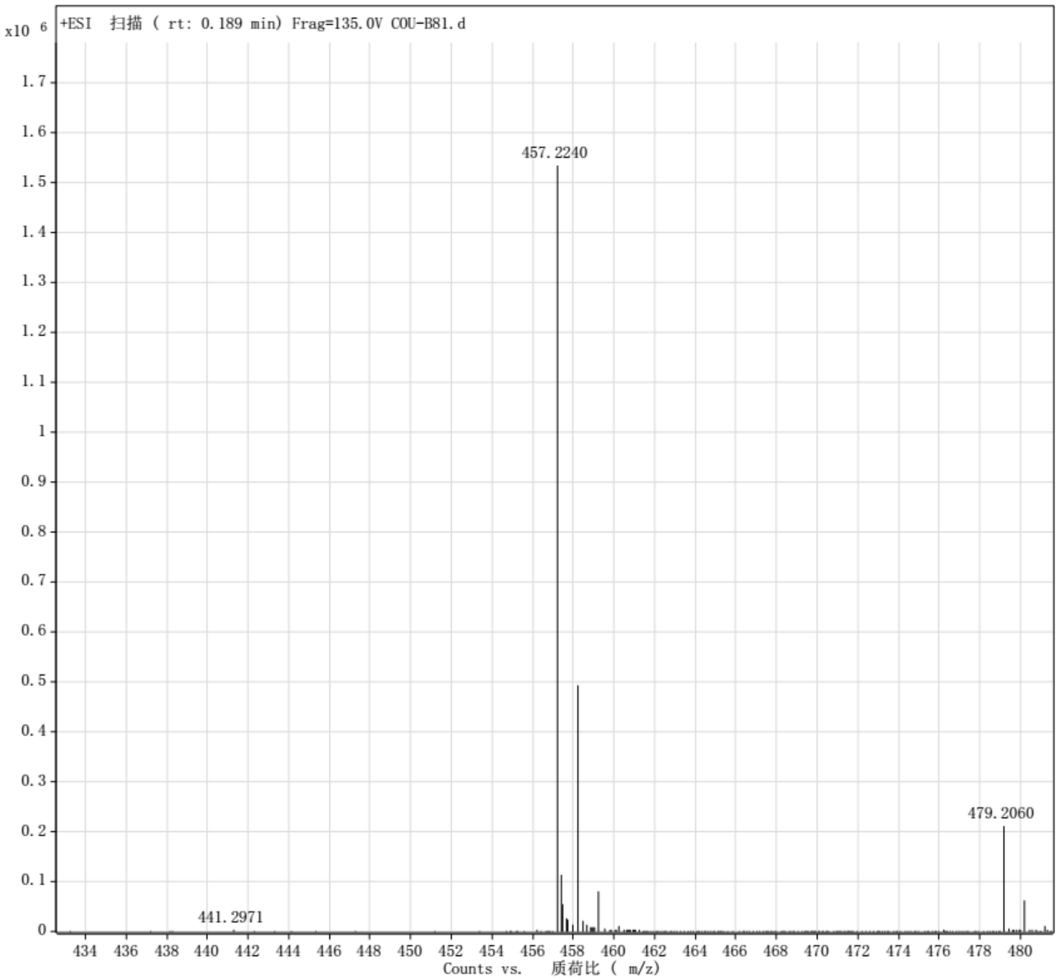

$^{1}$H NMR spectrum of **5f**

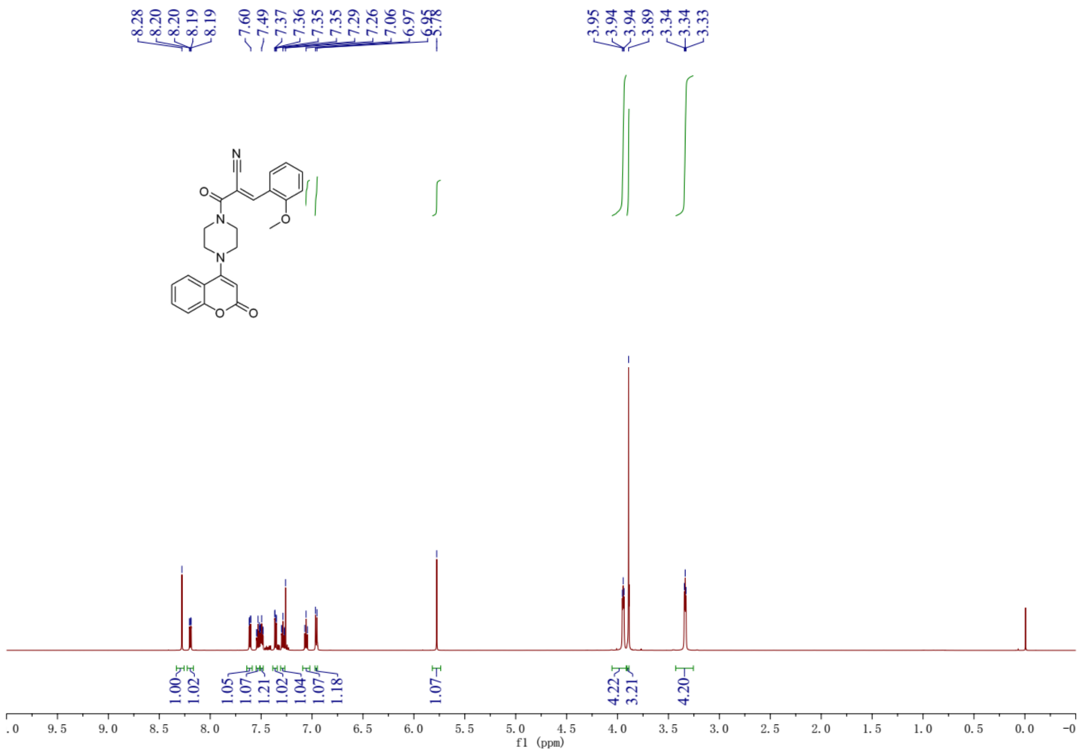

$^{13}$C NMR spectrum of **5f**

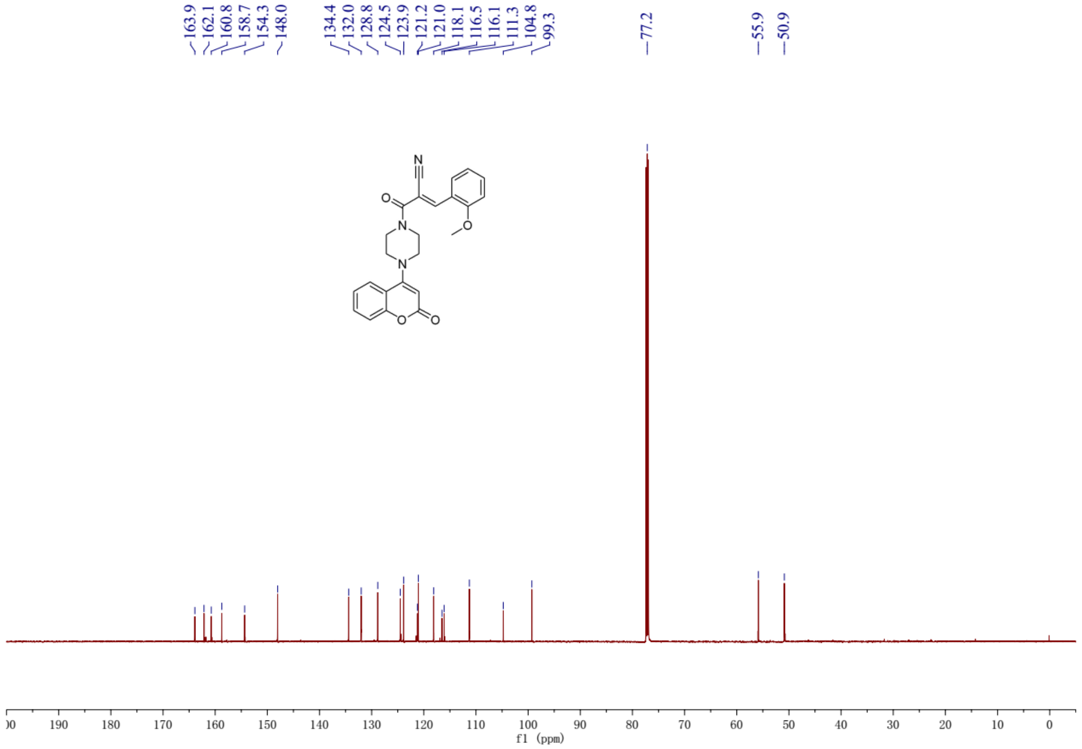

HR-MS (ESI) spectrum of **5f**

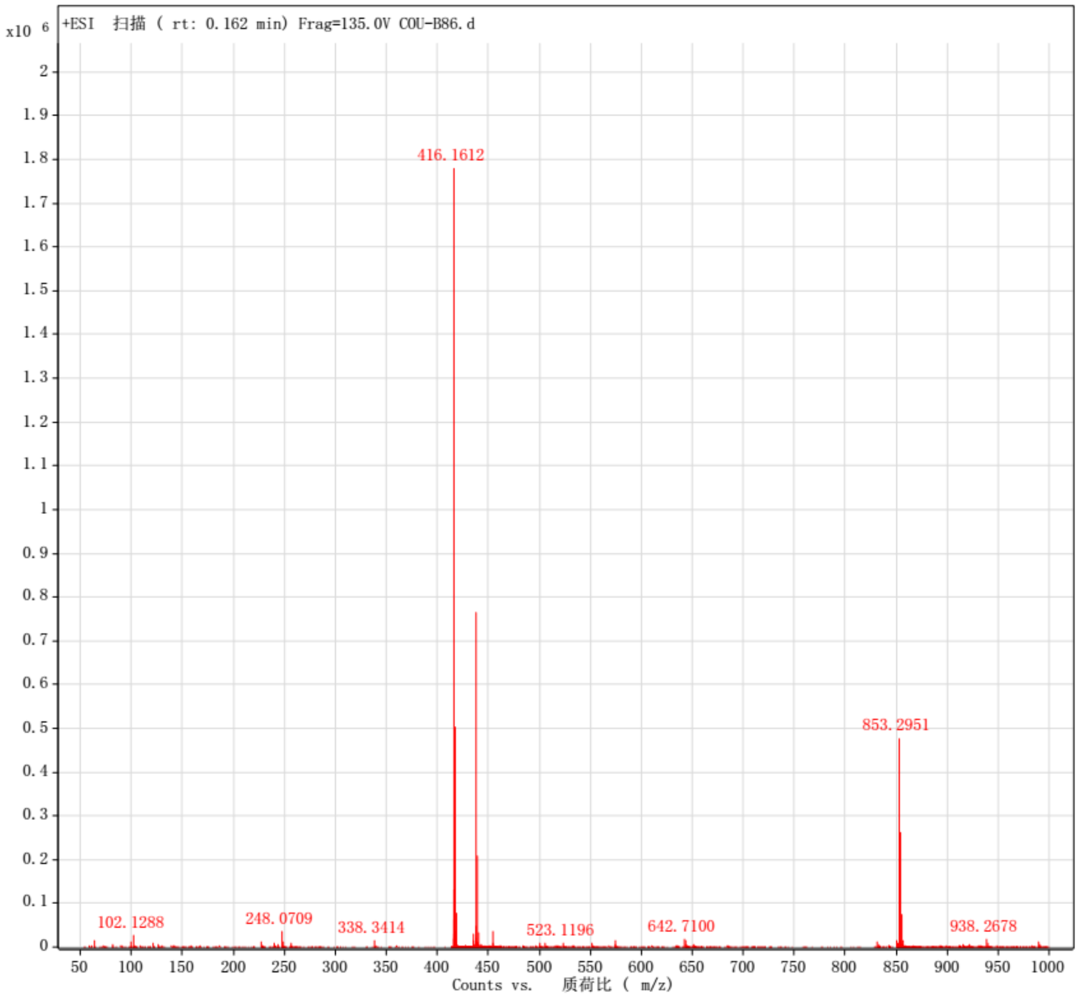

$^{1}$H NMR spectrum of **5g**

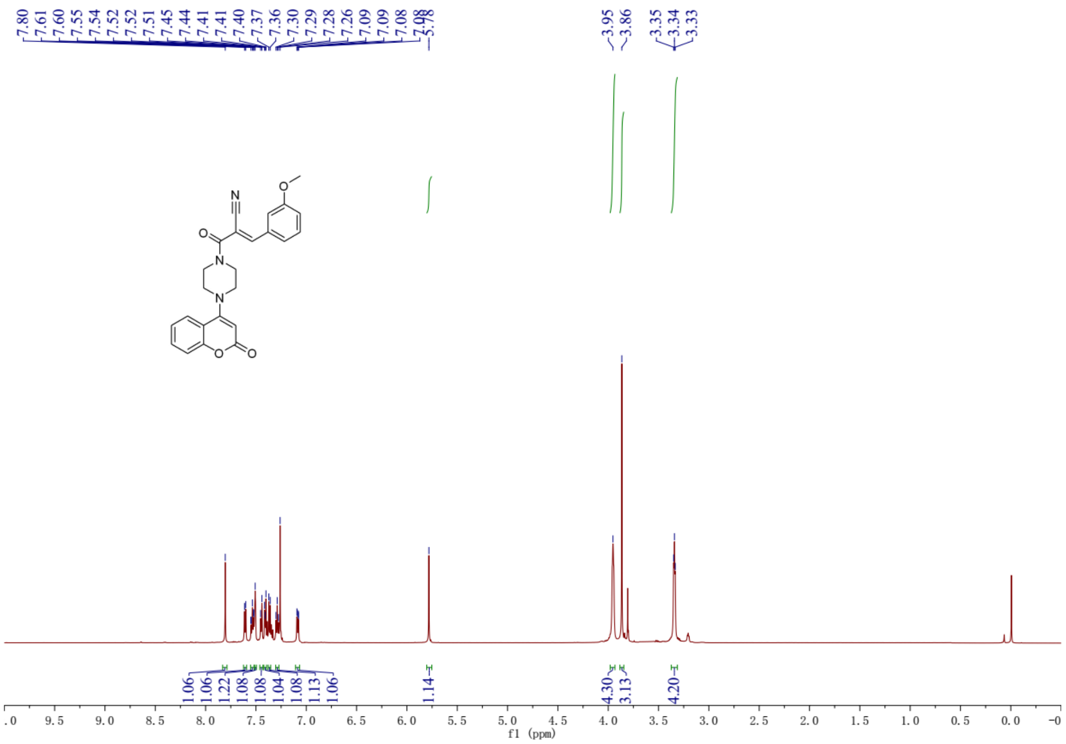

$^{13}$C NMR spectrum of **5g**

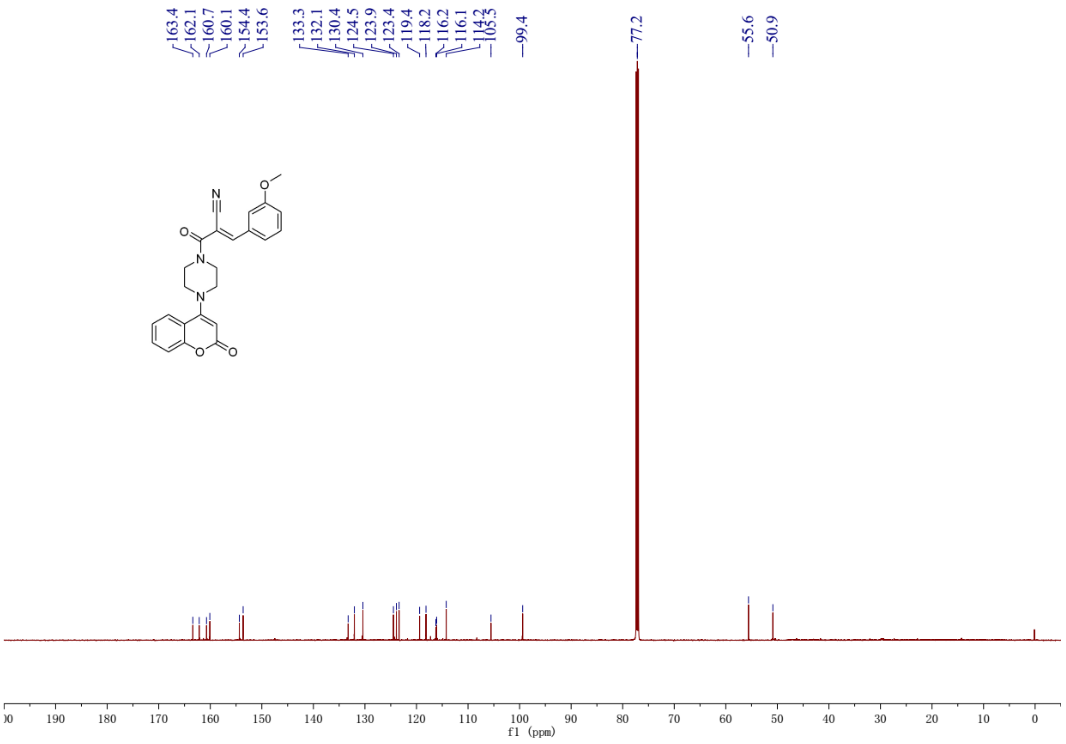

HR-MS (ESI) spectrum of **5g**

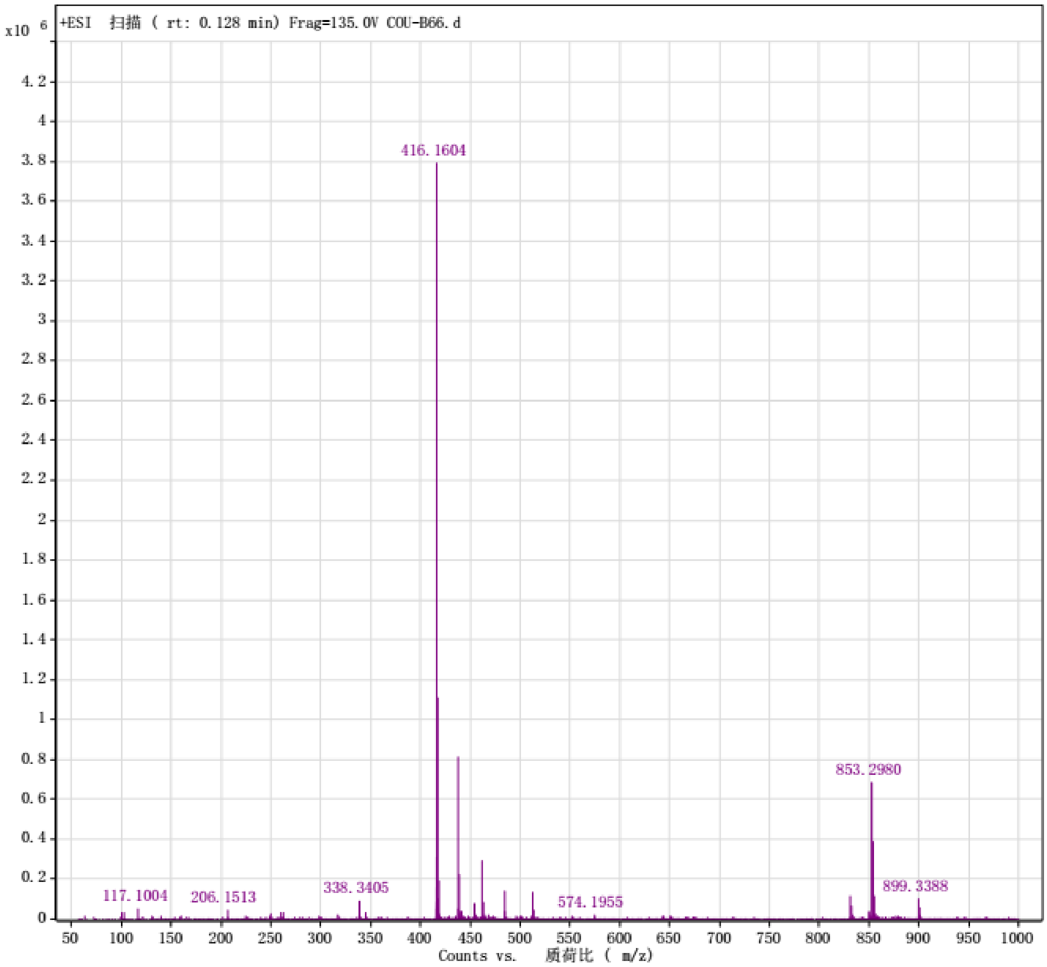

$^{1}$H NMR spectrum of **5h**

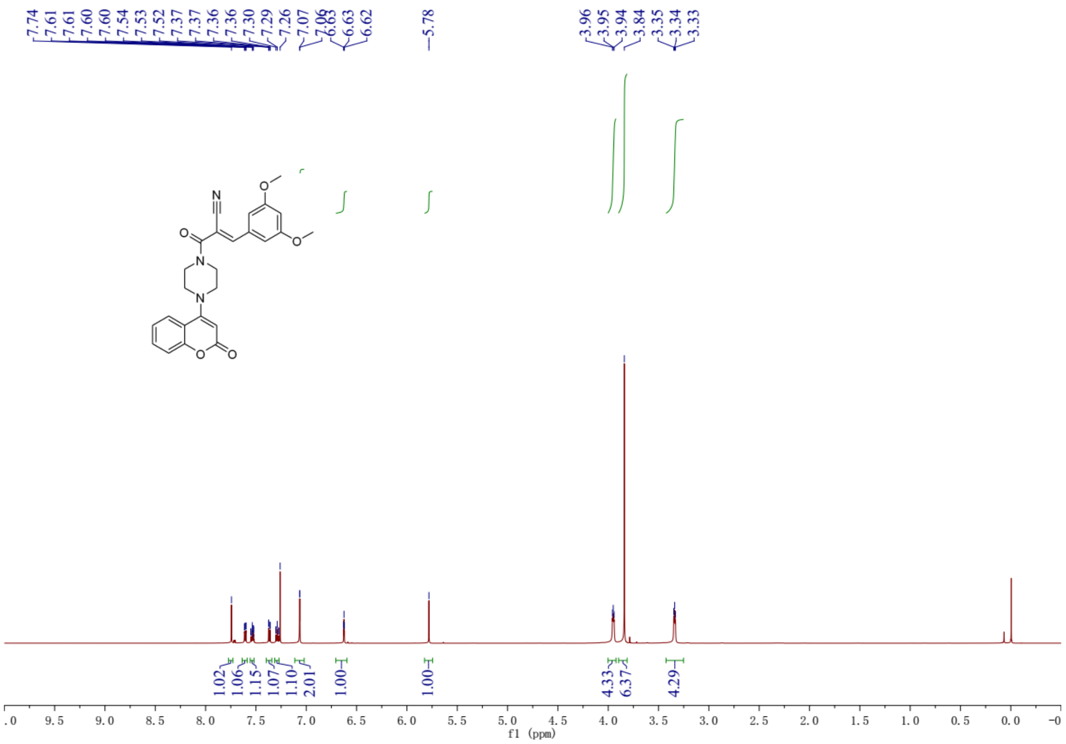

$^{13}$C NMR spectrum of **5h**

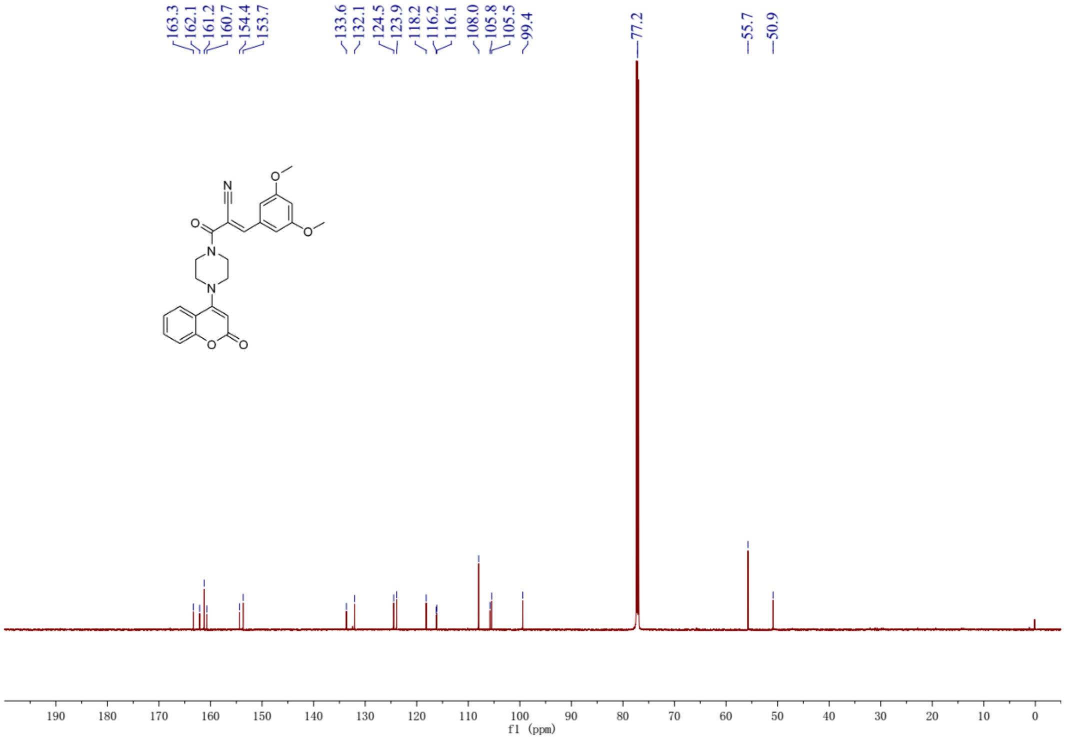

HR-MS (ESI) spectrum of **5h**

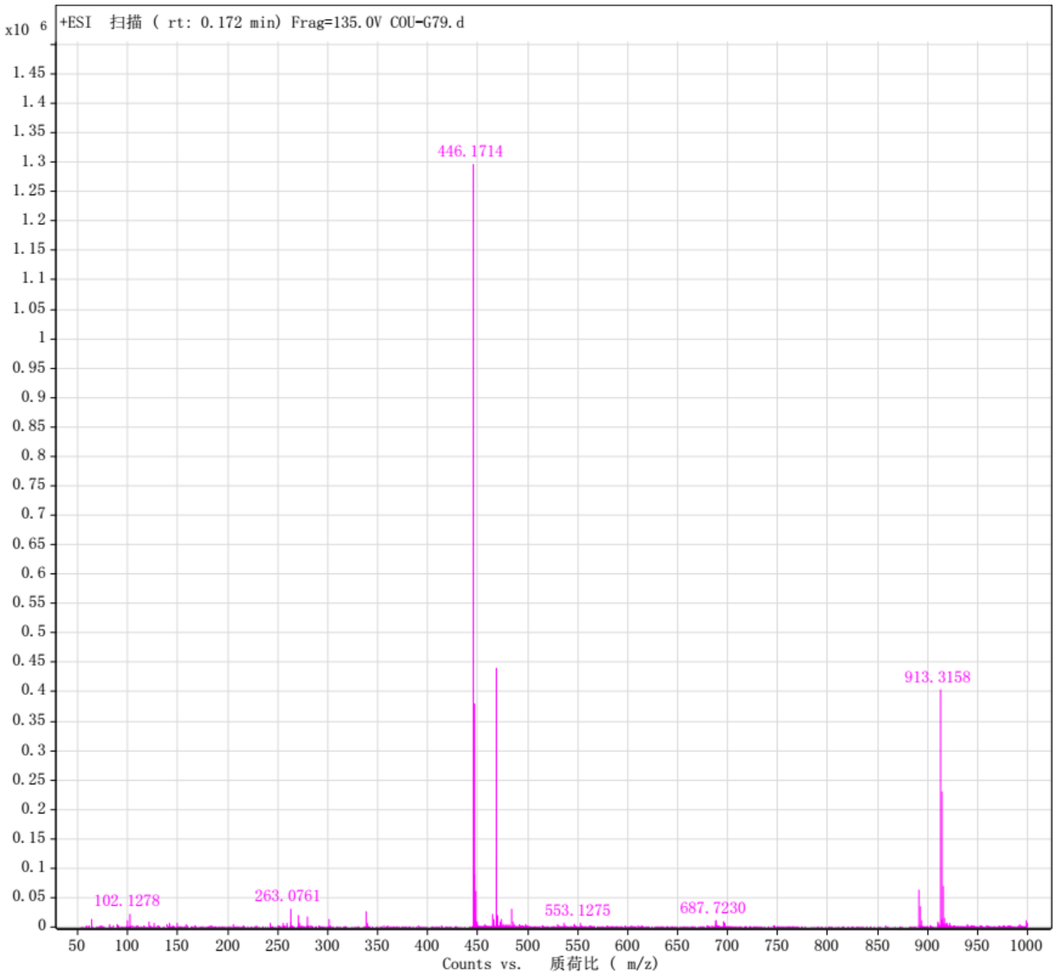

<sup></sup>

$^{1}$H NMR spectrum of **5i**

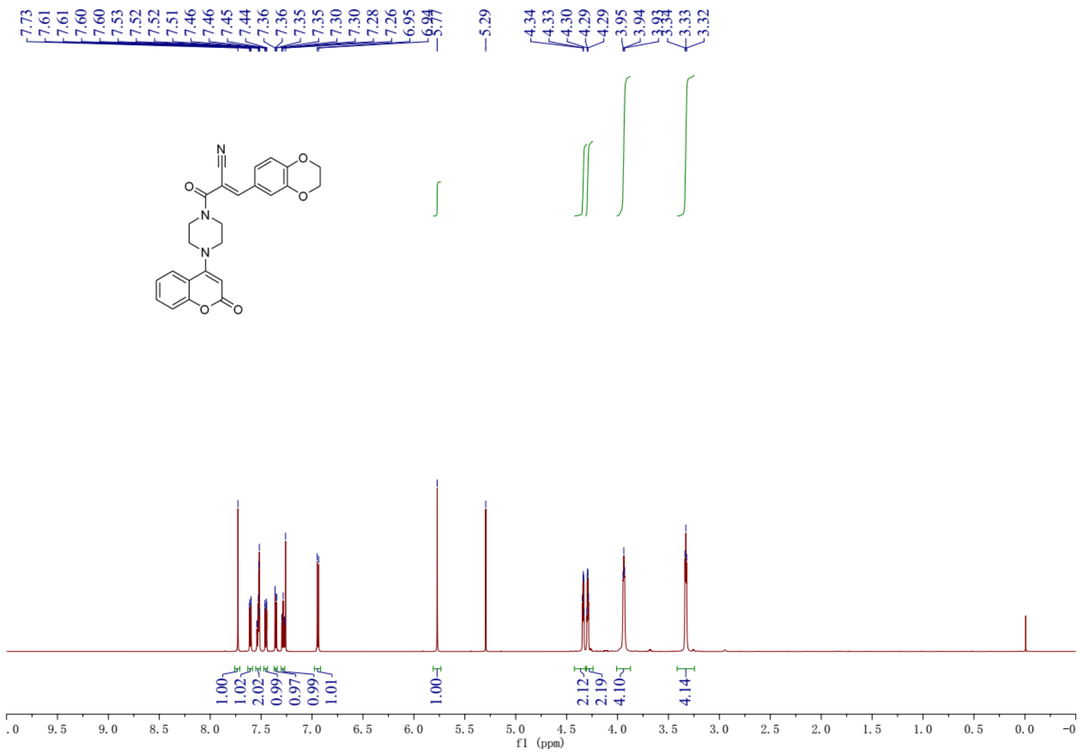

$^{13}$C NMR spectrum of **5i**

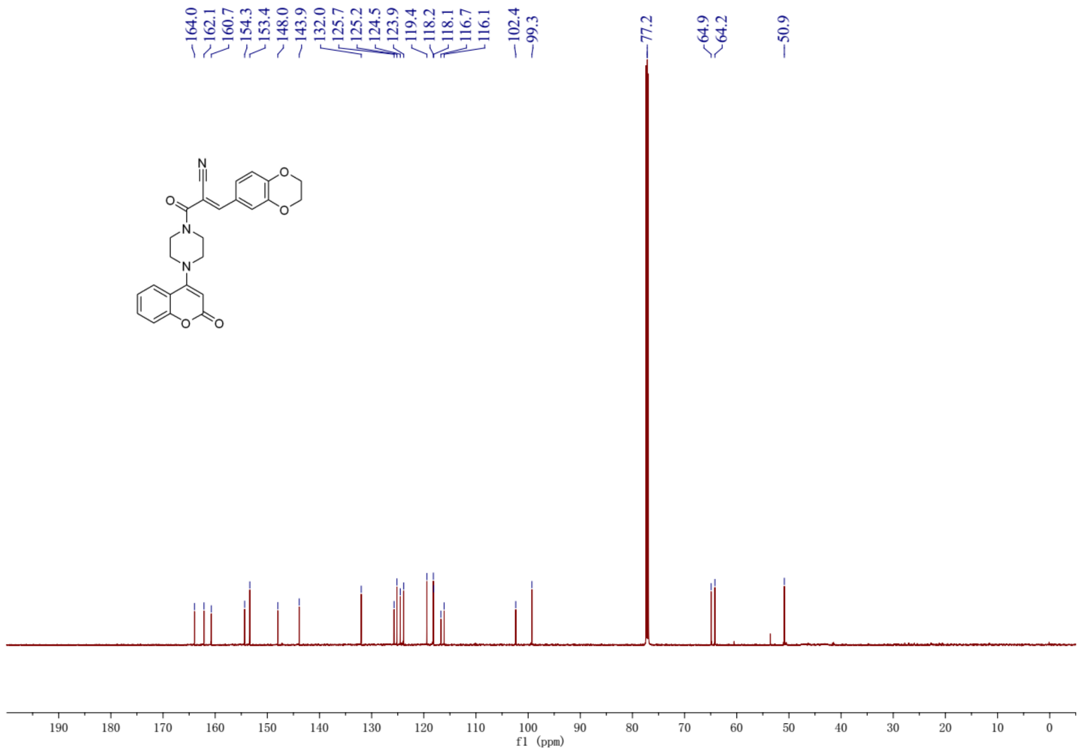

HR-MS (ESI) spectrum of **5i**

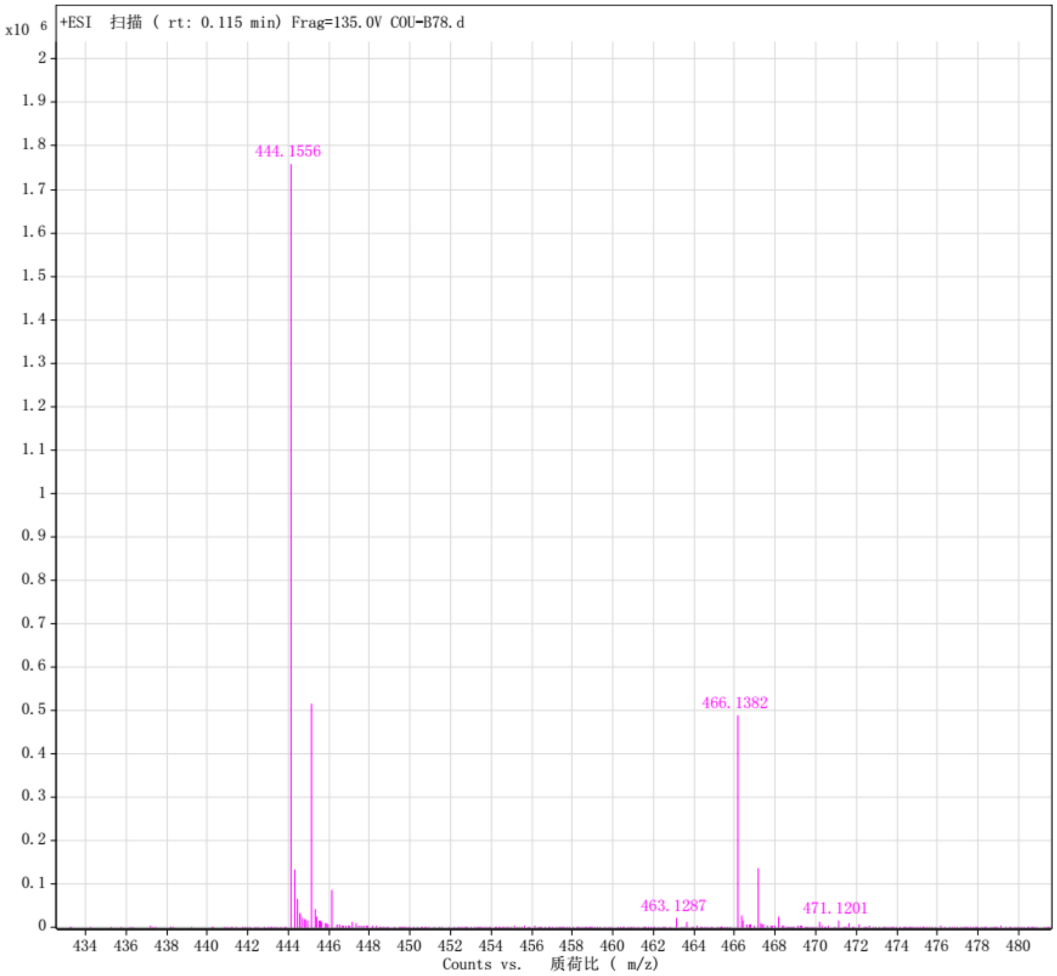

$^{1}$H NMR spectrum of **5j**

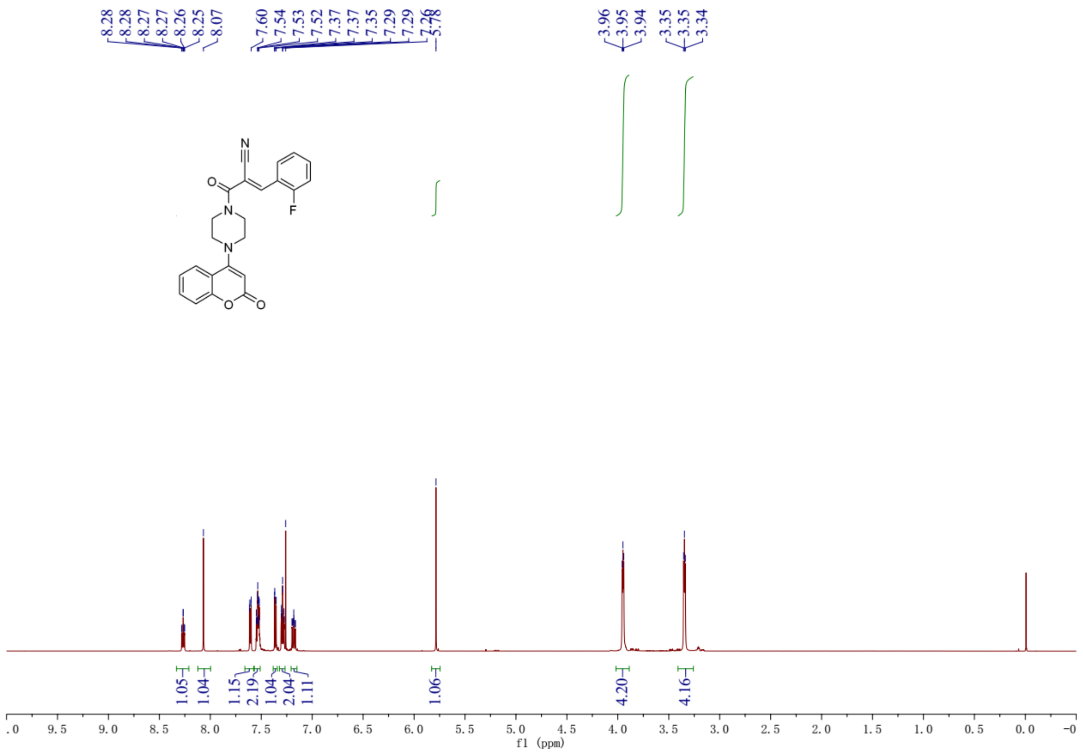


$^{13}$C NMR spectrum of **5j**

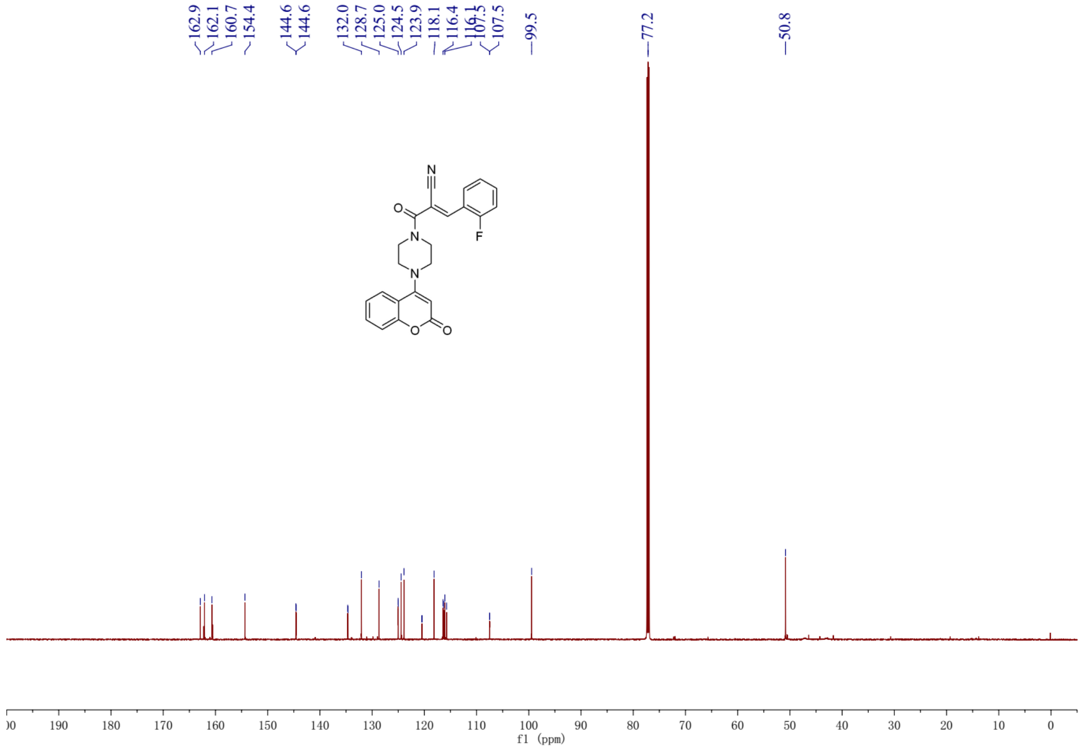

HR-MS (ESI) spectrum of **5j**

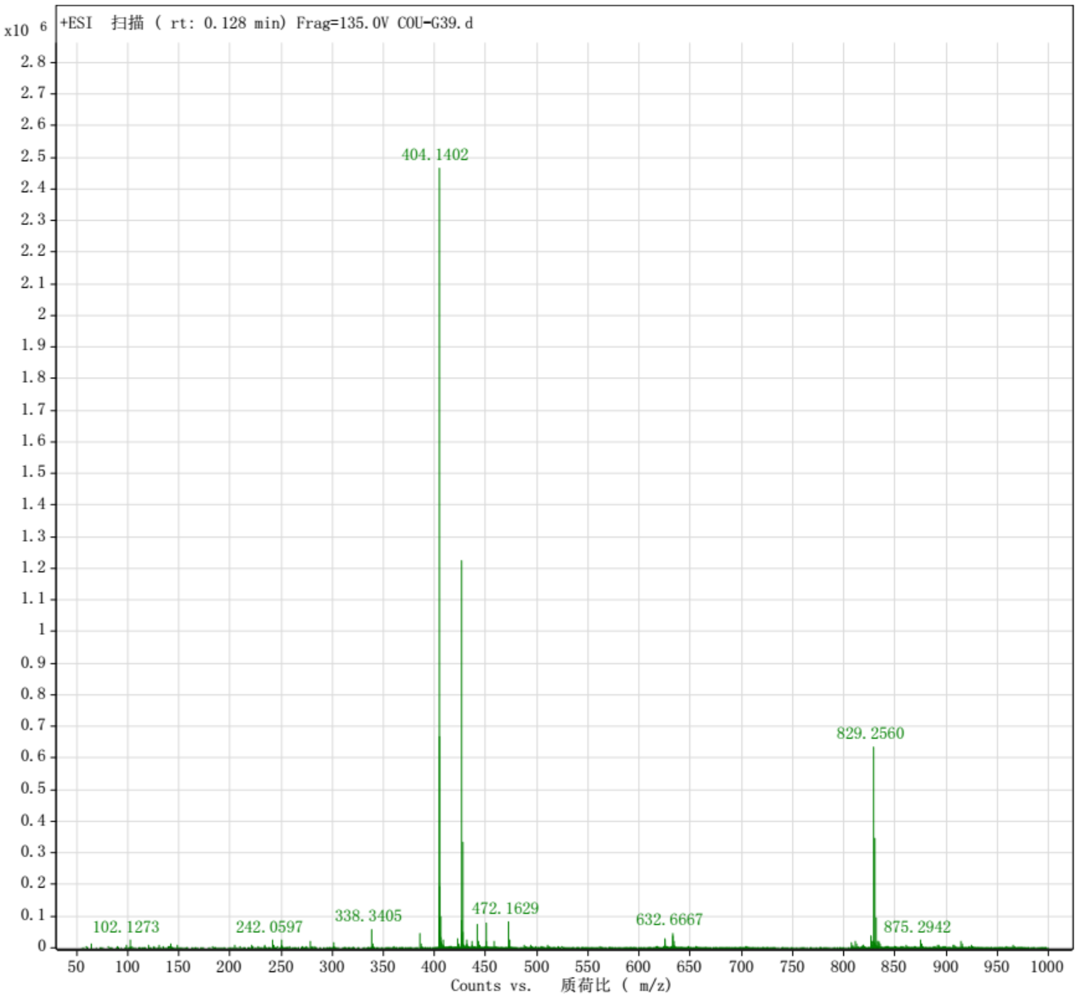

$^{1}$H NMR spectrum of **5k**

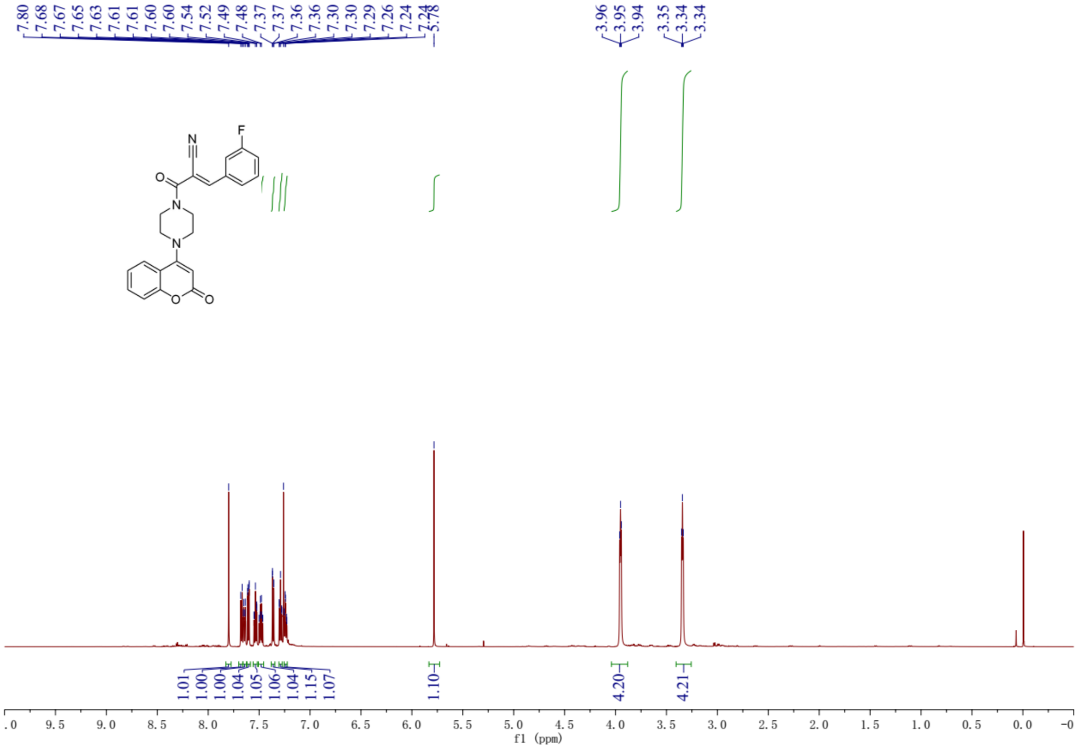


$^{13}$C NMR spectrum of **5k**

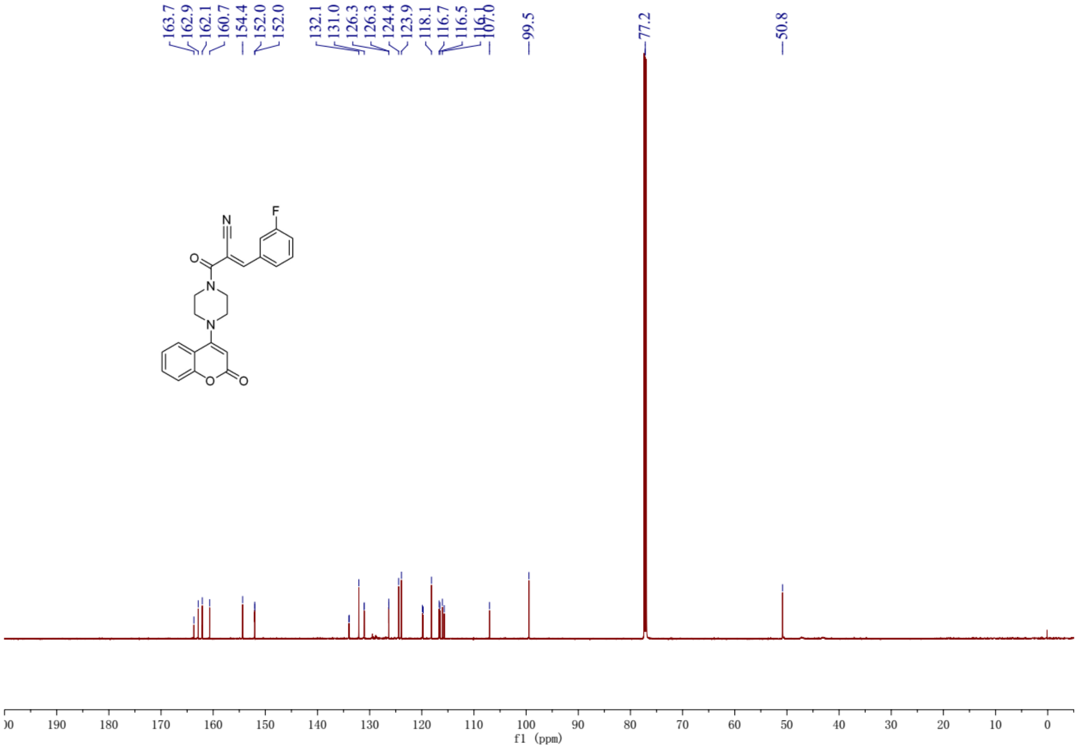

HR-MS (ESI) spectrum of **5k**

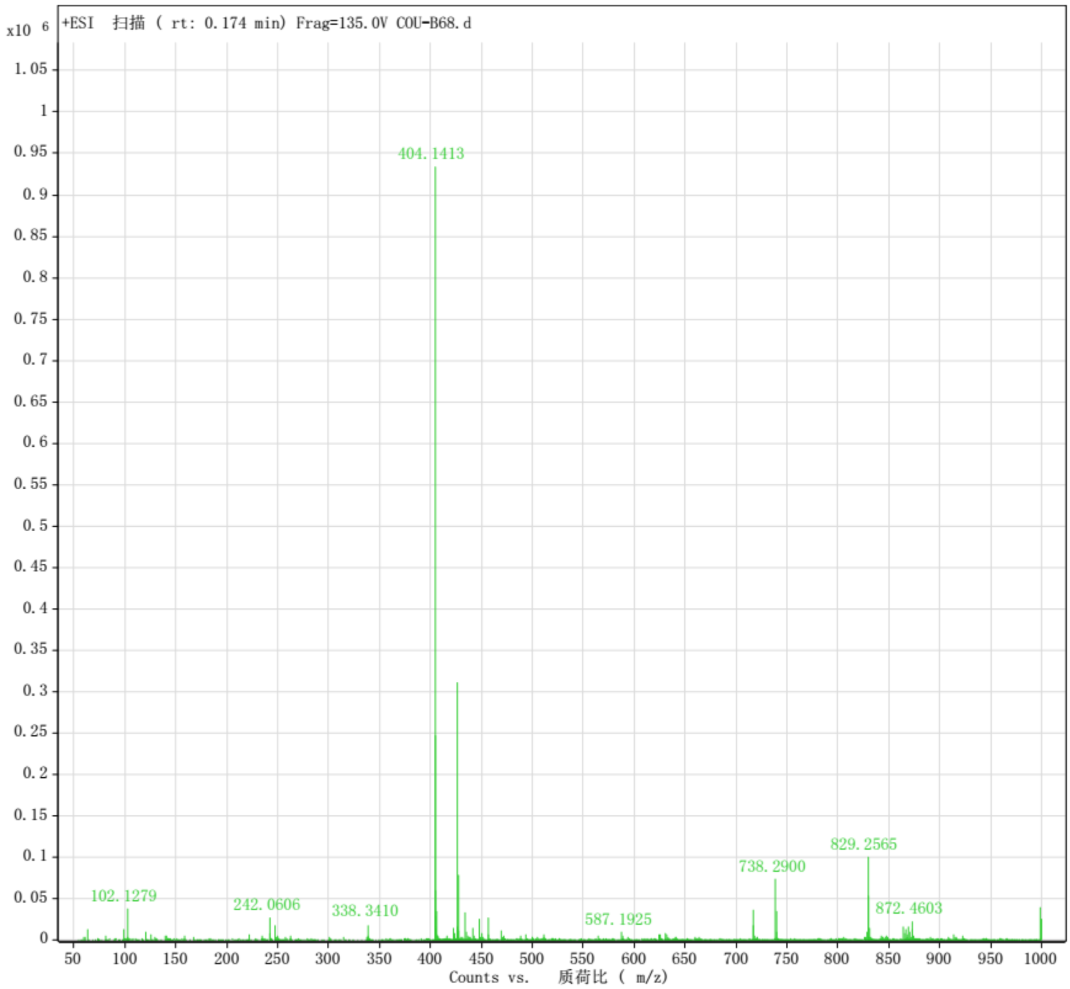

$^{1}$H NMR spectrum of **5l**

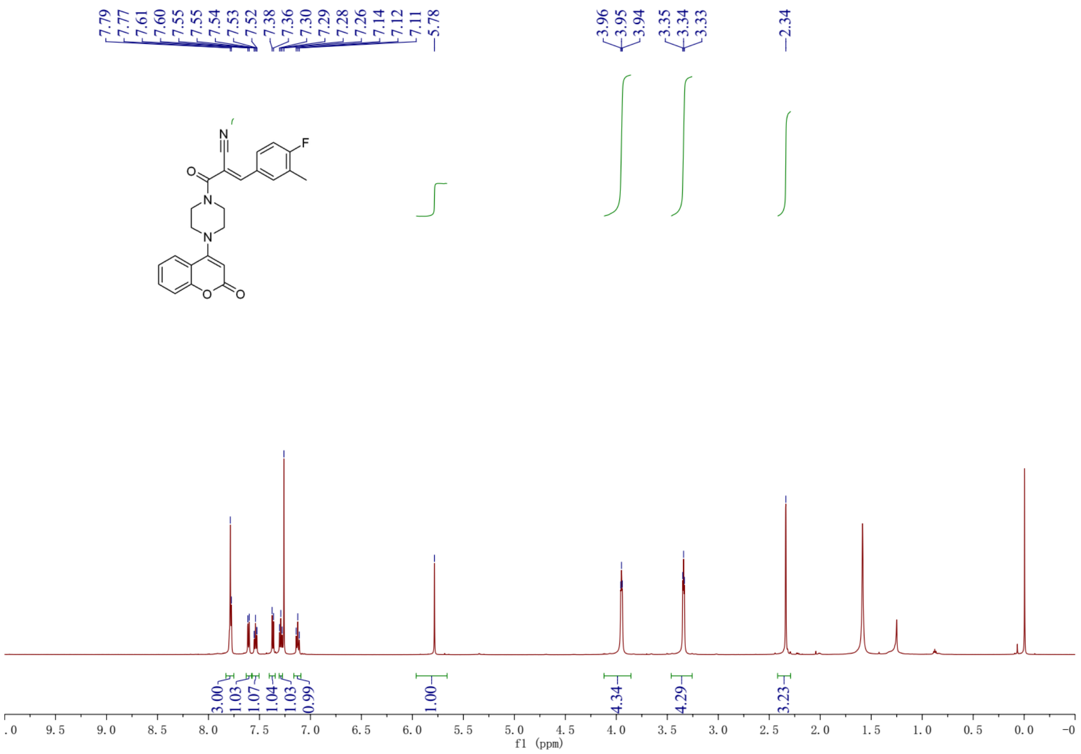

$^{13}$C NMR spectrum of **5l**

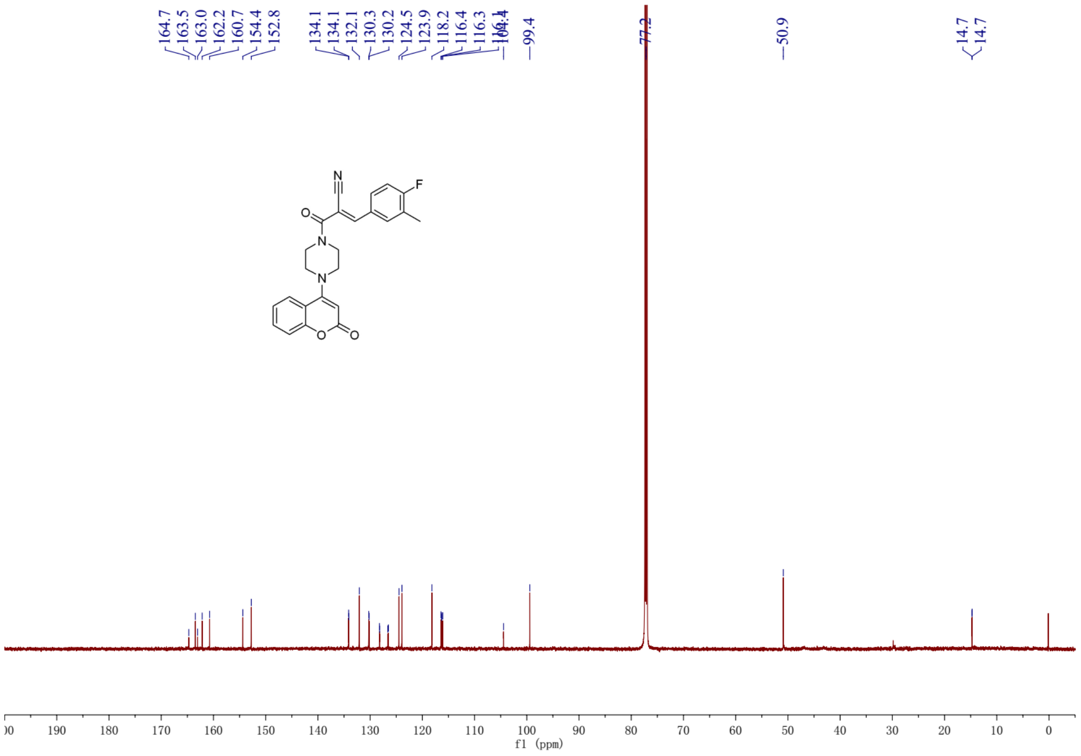

HR-MS (ESI) spectrum of **5l**

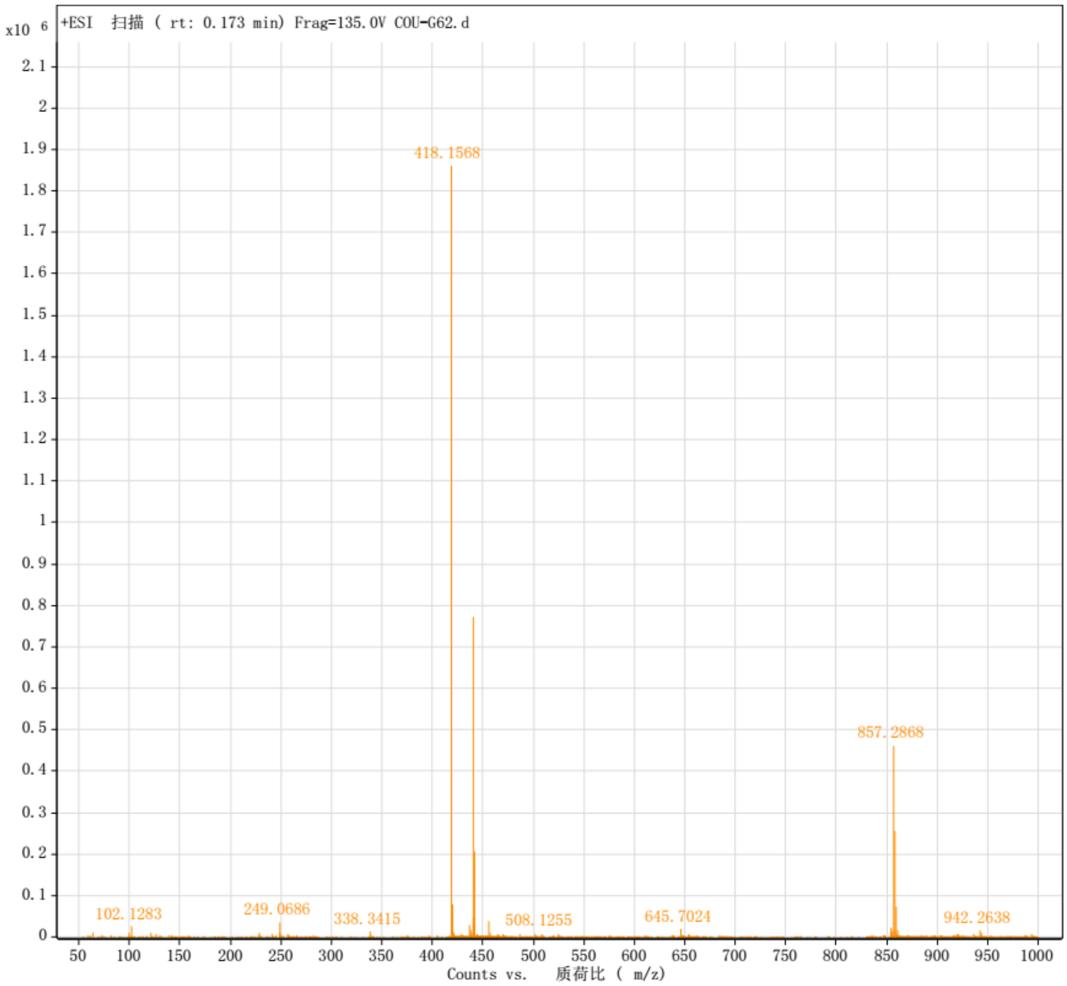

$^1$H NMR spectrum of **5m**

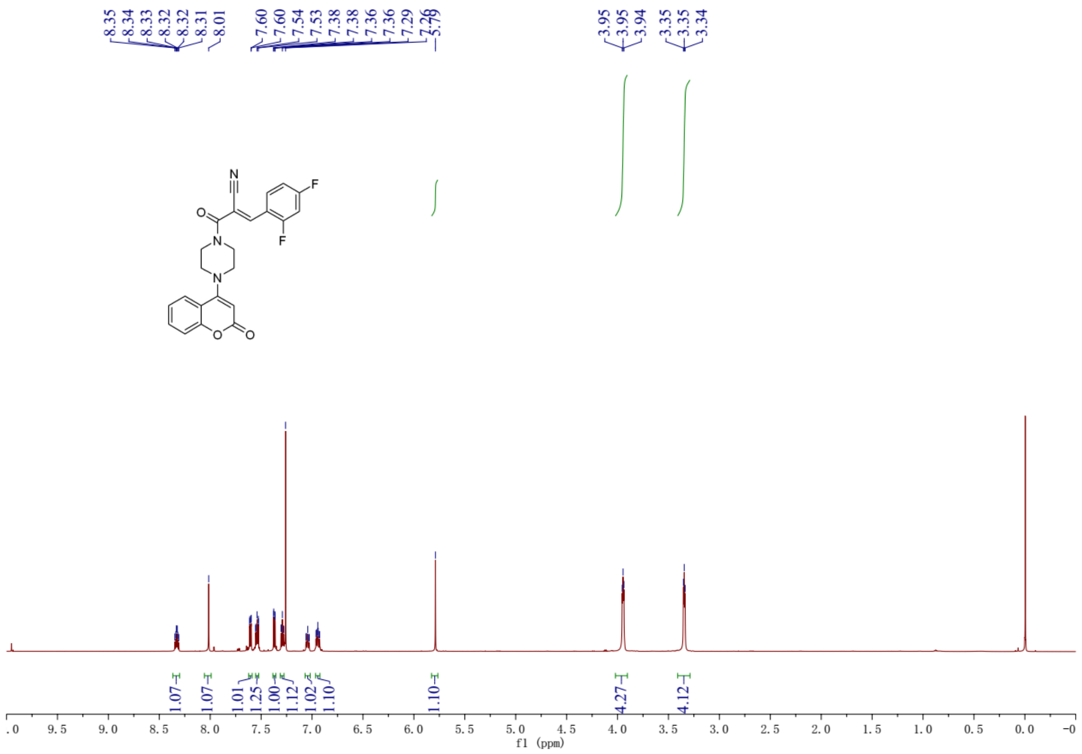

$^{13}$C NMR spectrum of **5m**

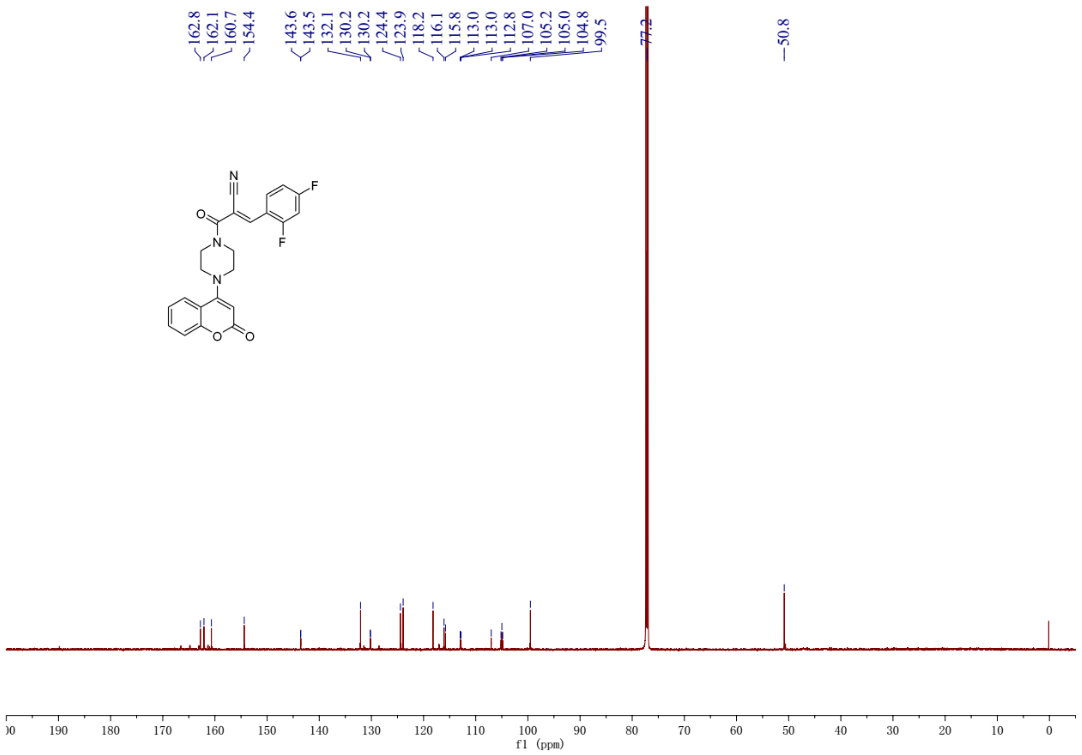

HR-MS (ESI) spectrum of **5m**

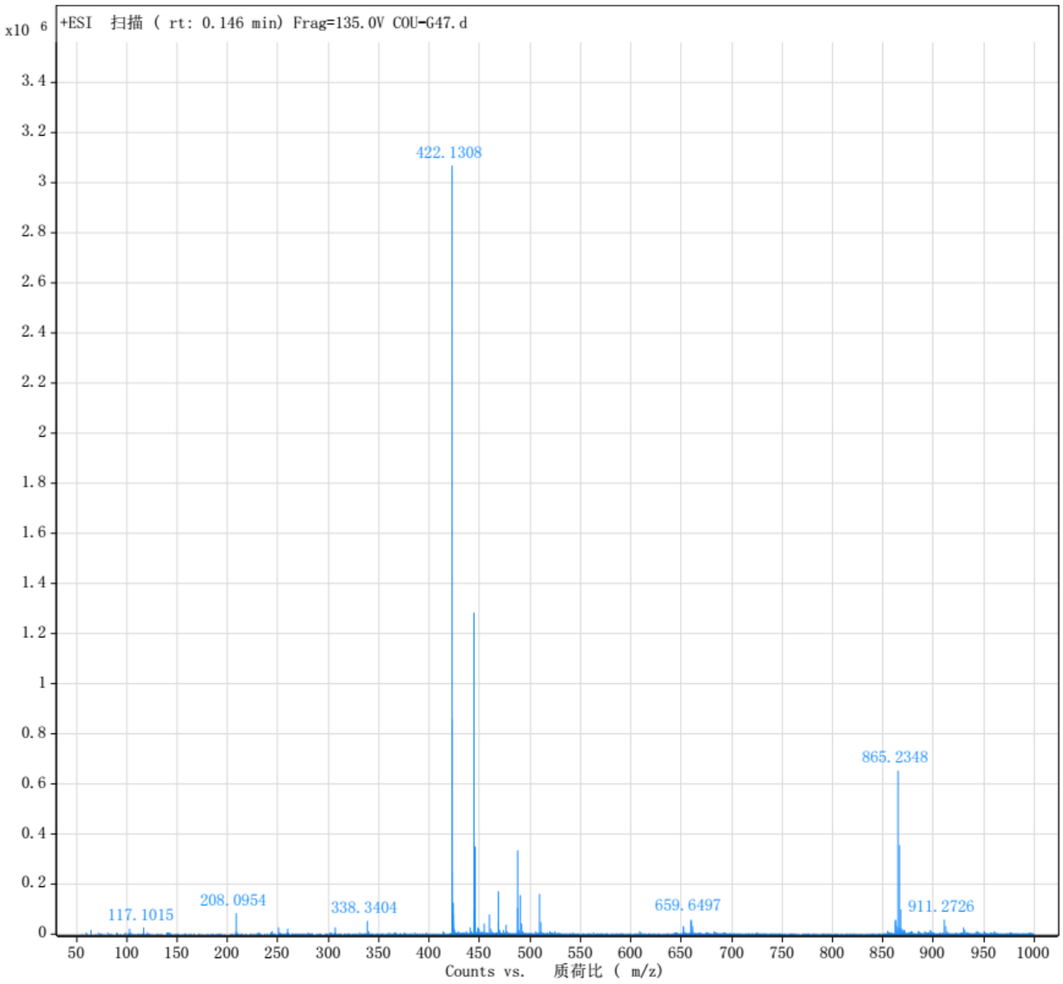

$^{1}H$ NMR spectrum of **5n**

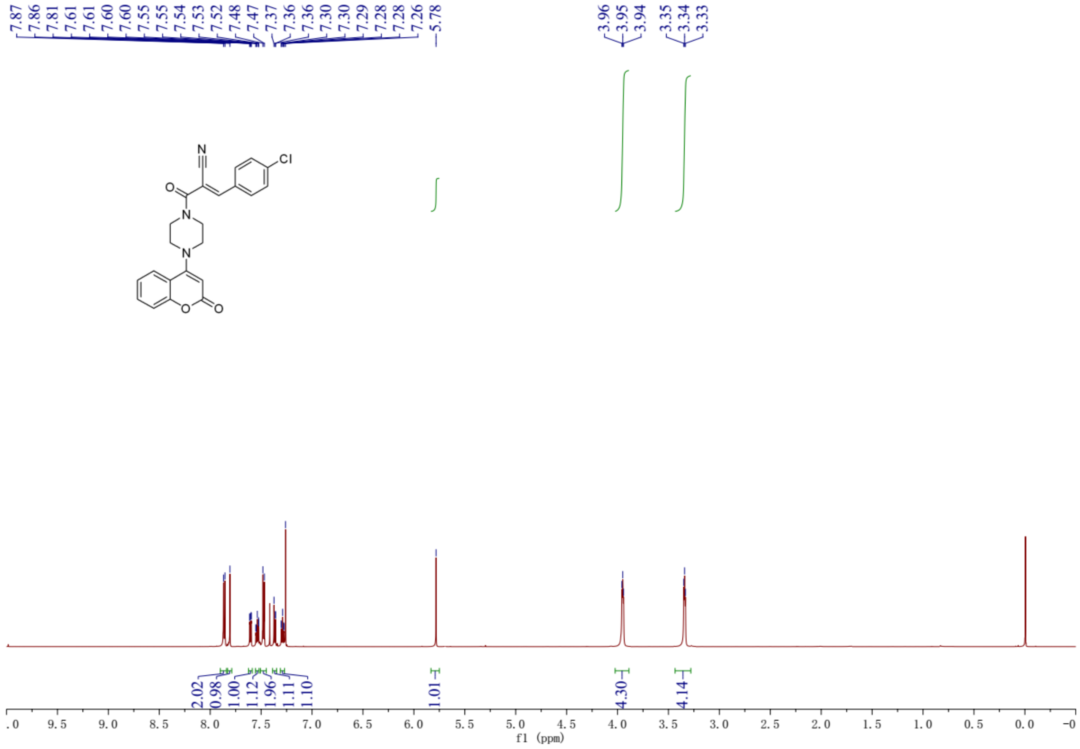


$^{13}C$ NMR spectrum of **5n**

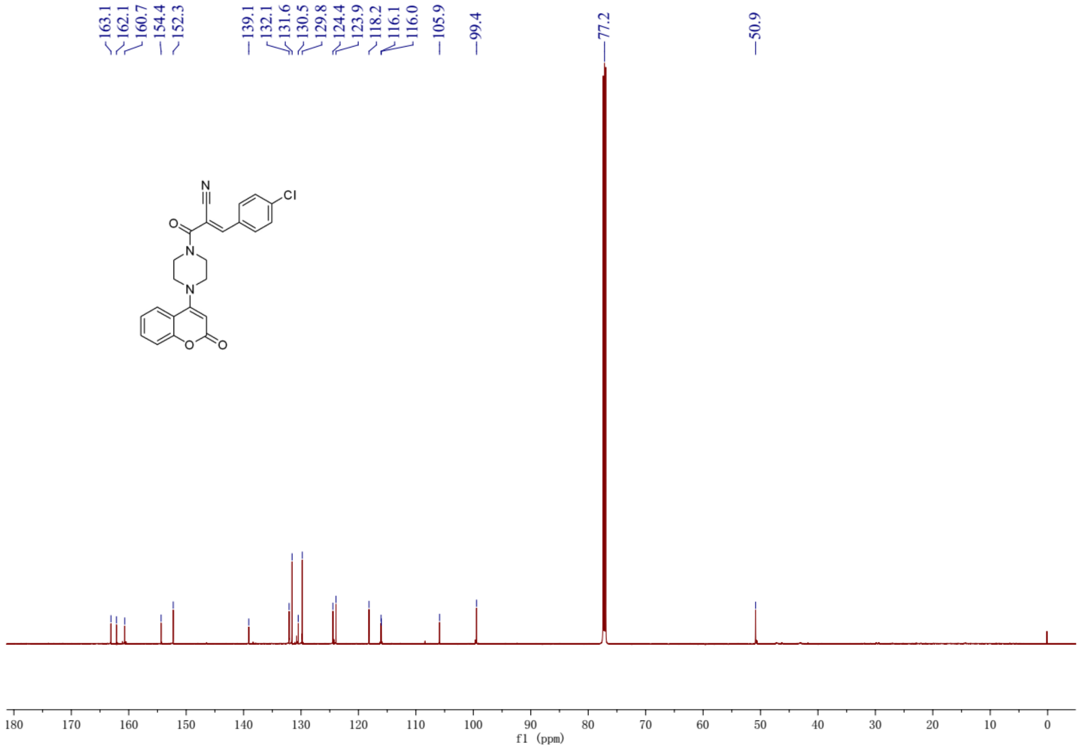

HR-MS (ESI) spectrum of **5n**

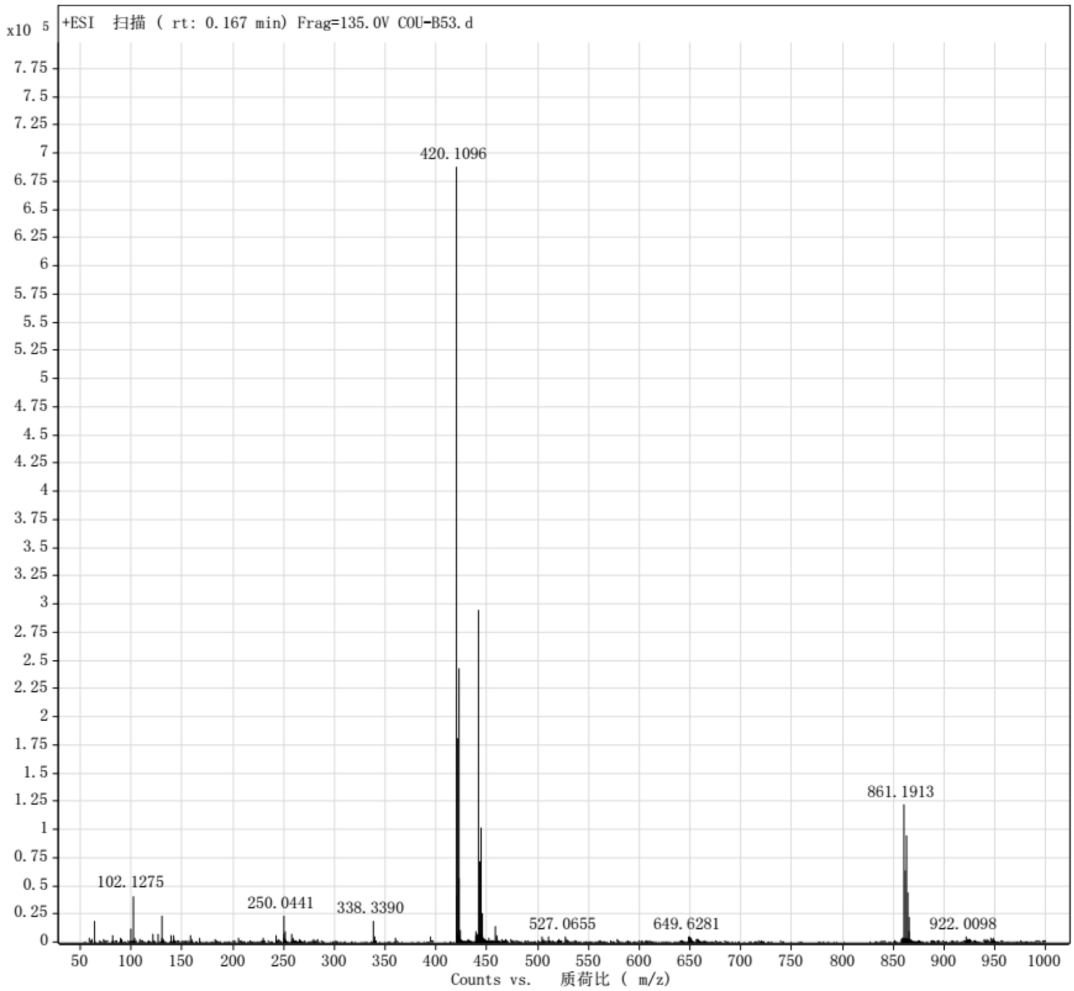

$^{1}$H NMR spectrum of **5o**

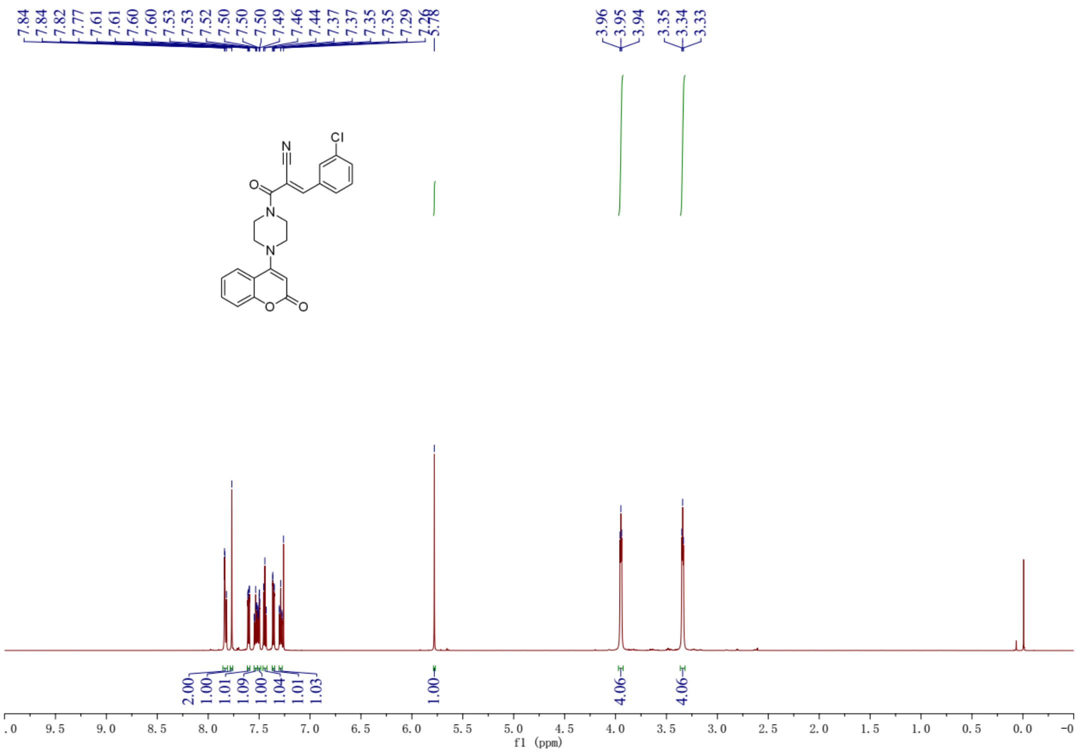

$^{13}$C NMR spectrum of **5o**

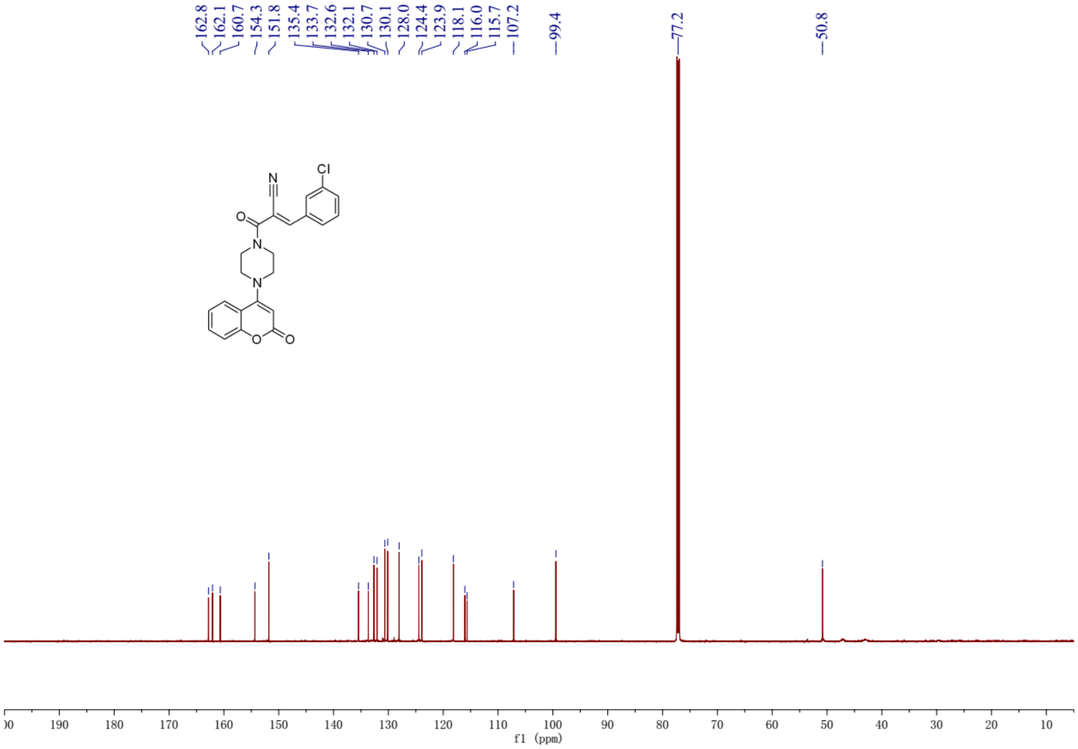

HR-MS (ESI) spectrum of **5o**

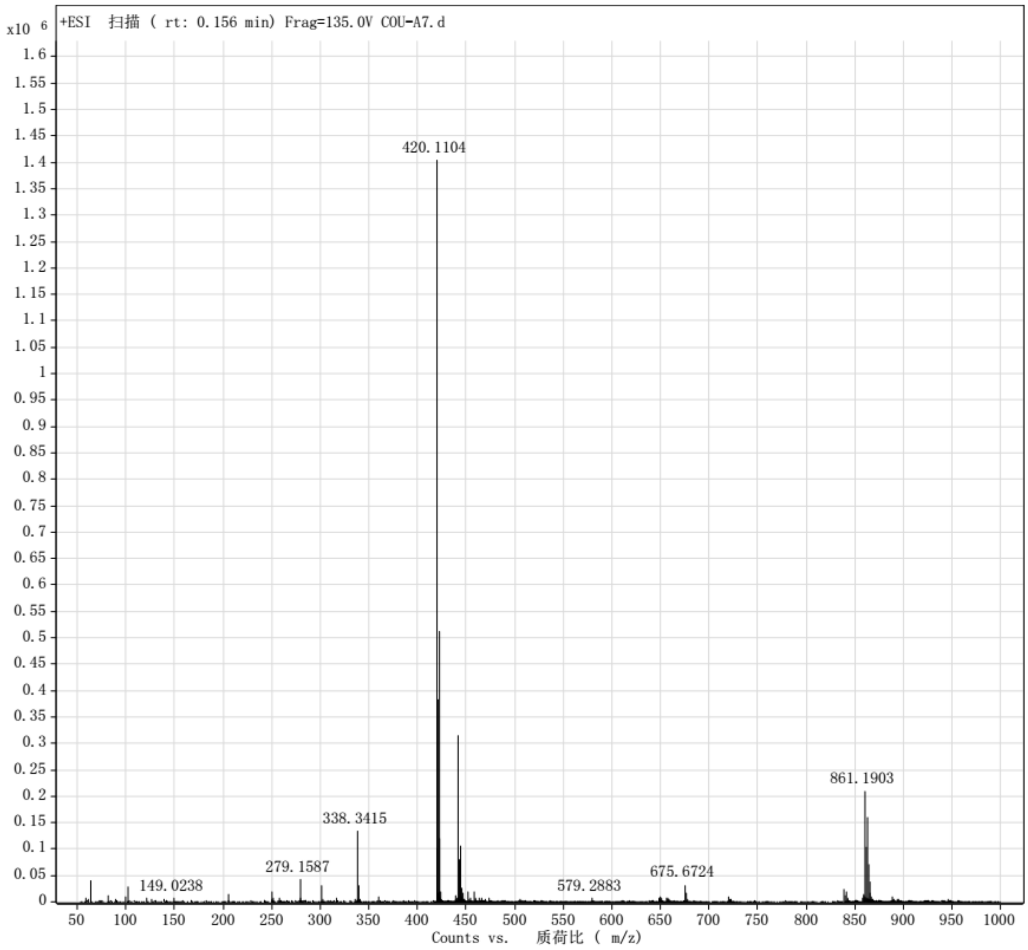

$^{1}$H NMR spectrum of **5p**

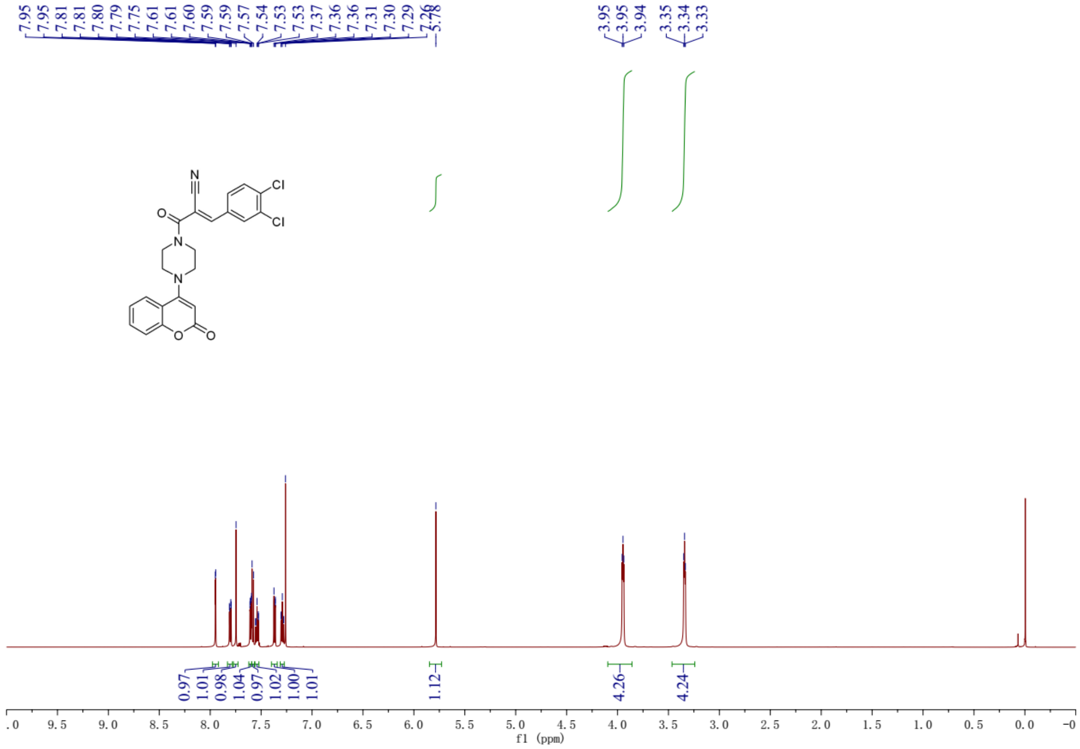


$^{13}$C NMR spectrum of **5p**

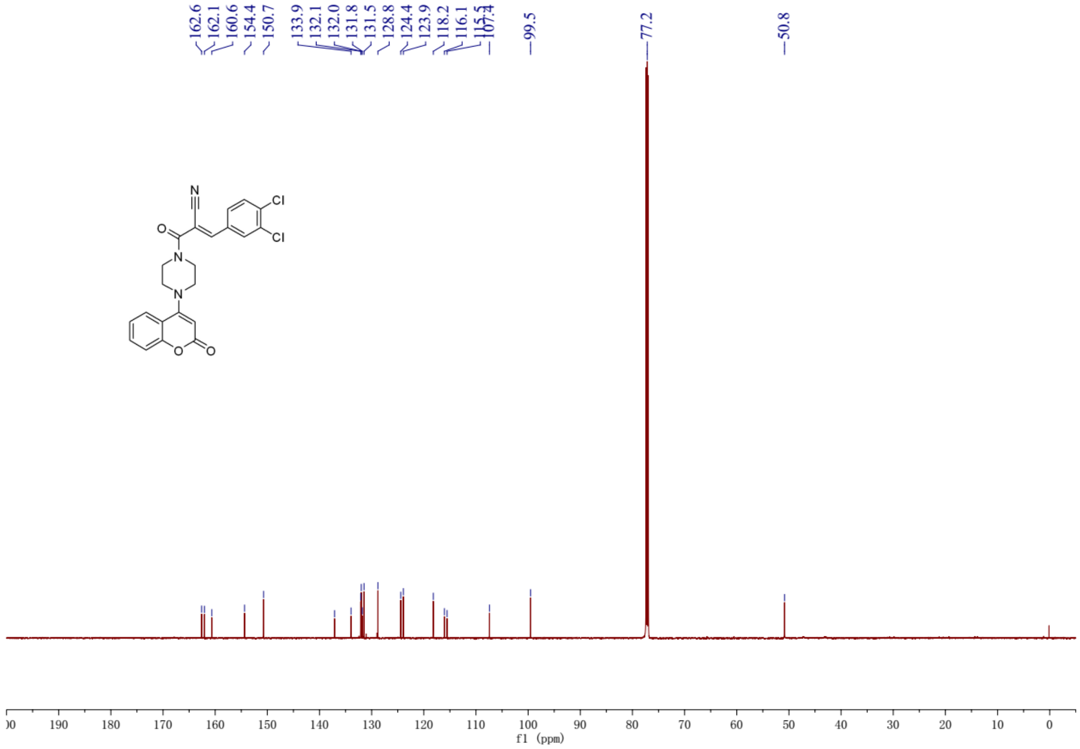

HR-MS (ESI) spectrum of **5p**

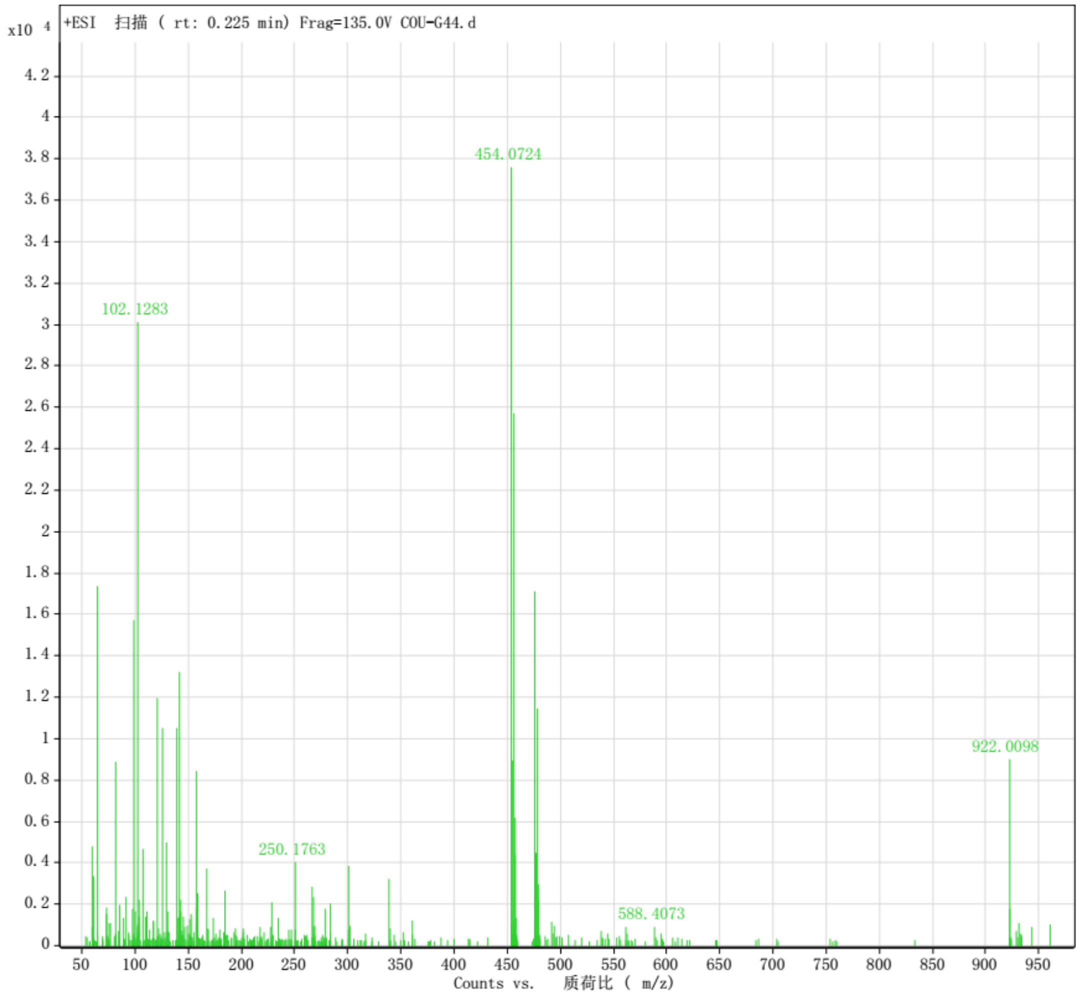

$^{1}$H NMR spectrum of **5q**

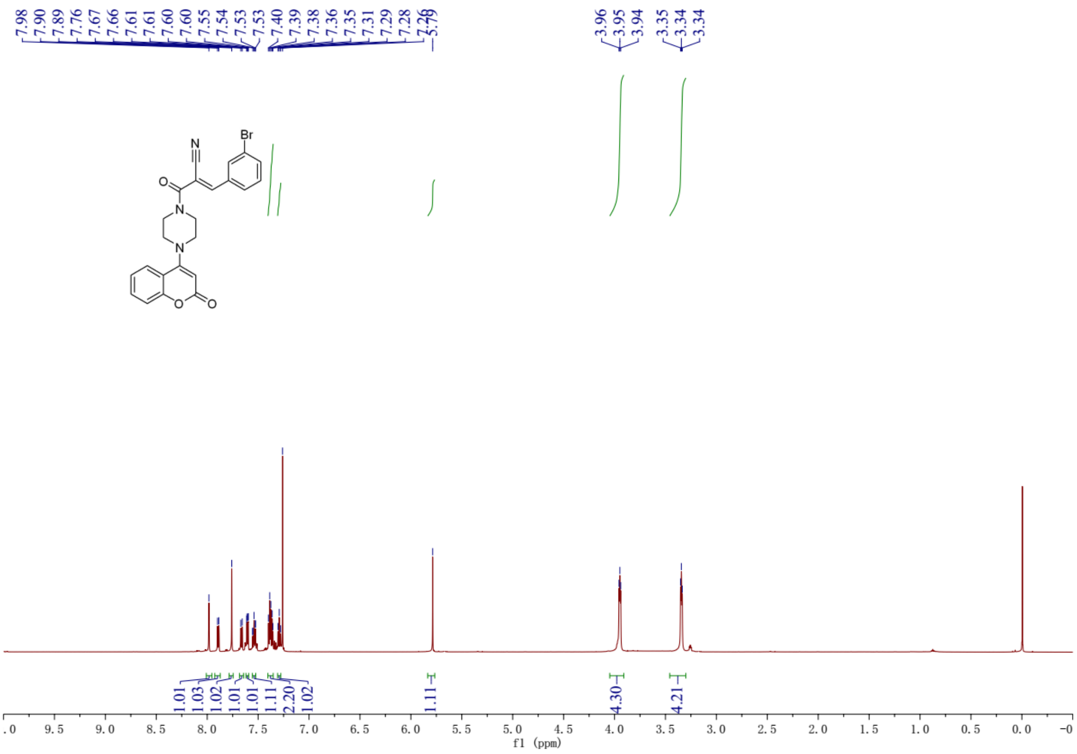

$^{13}$C NMR spectrum of **5q**

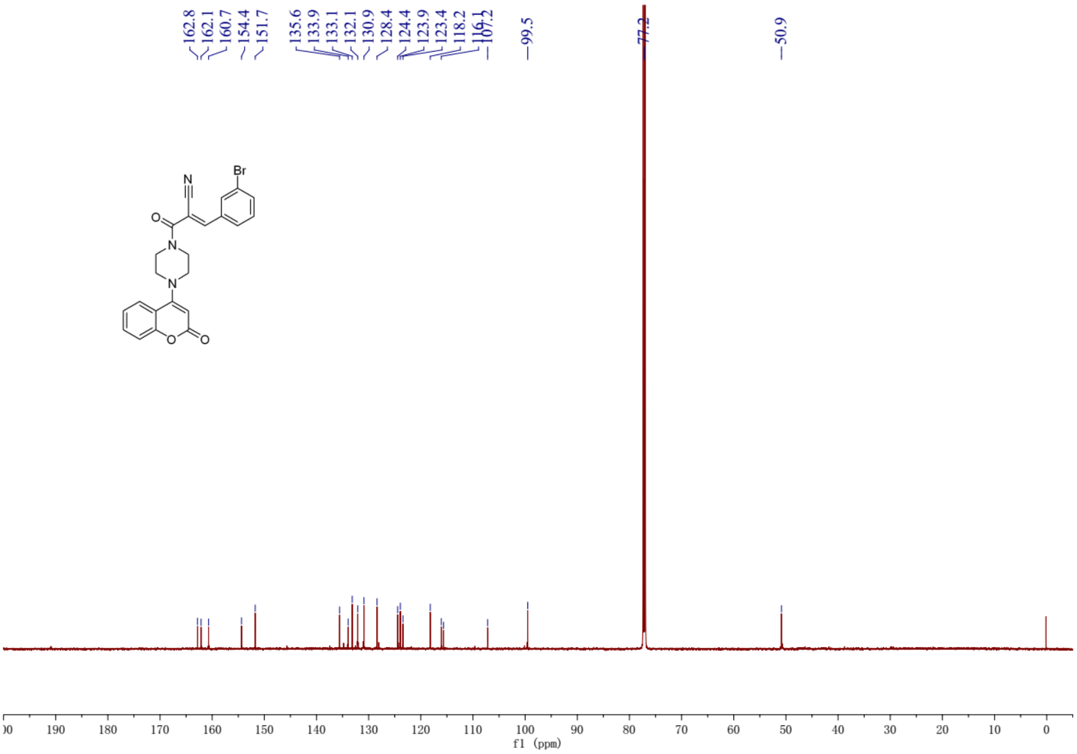

HR-MS (ESI) spectrum of **5q**

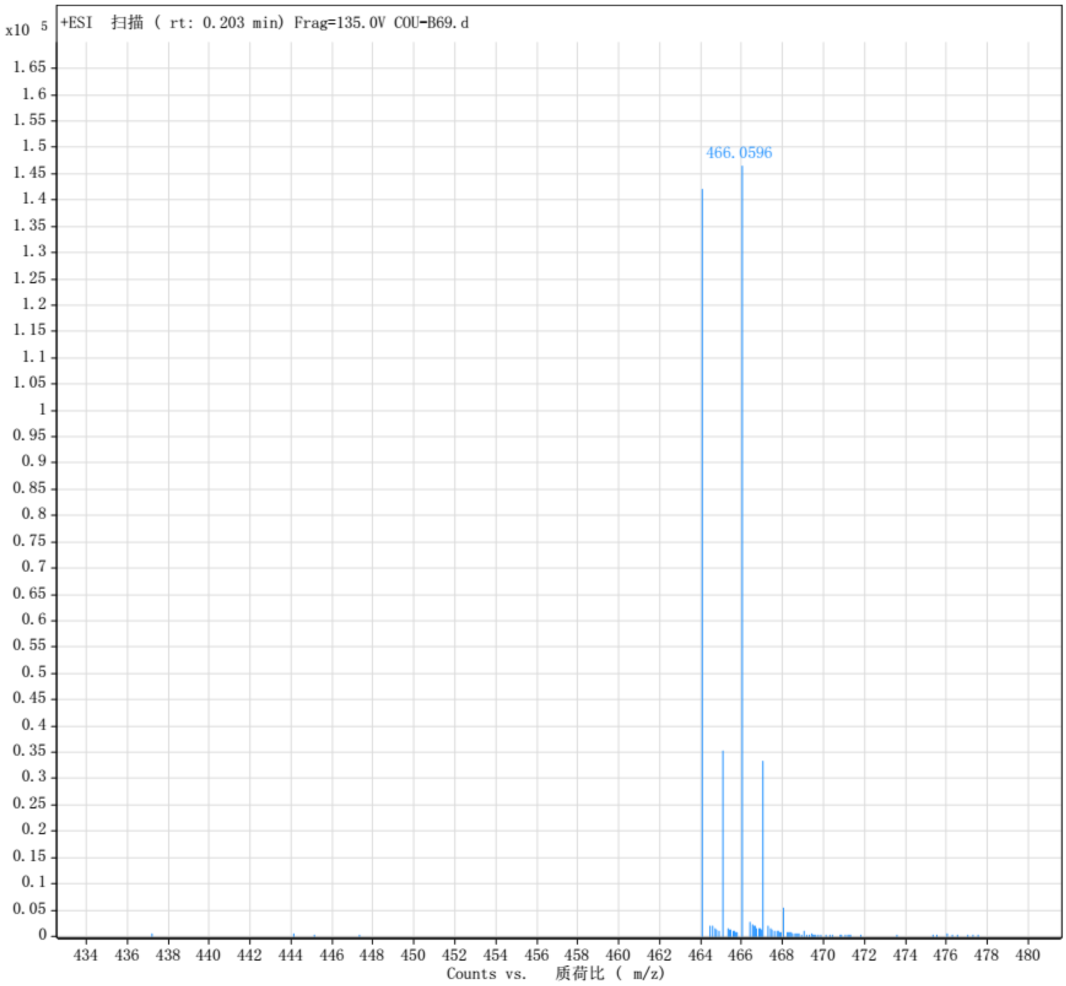

$^{1}$H NMR spectrum of **5r**

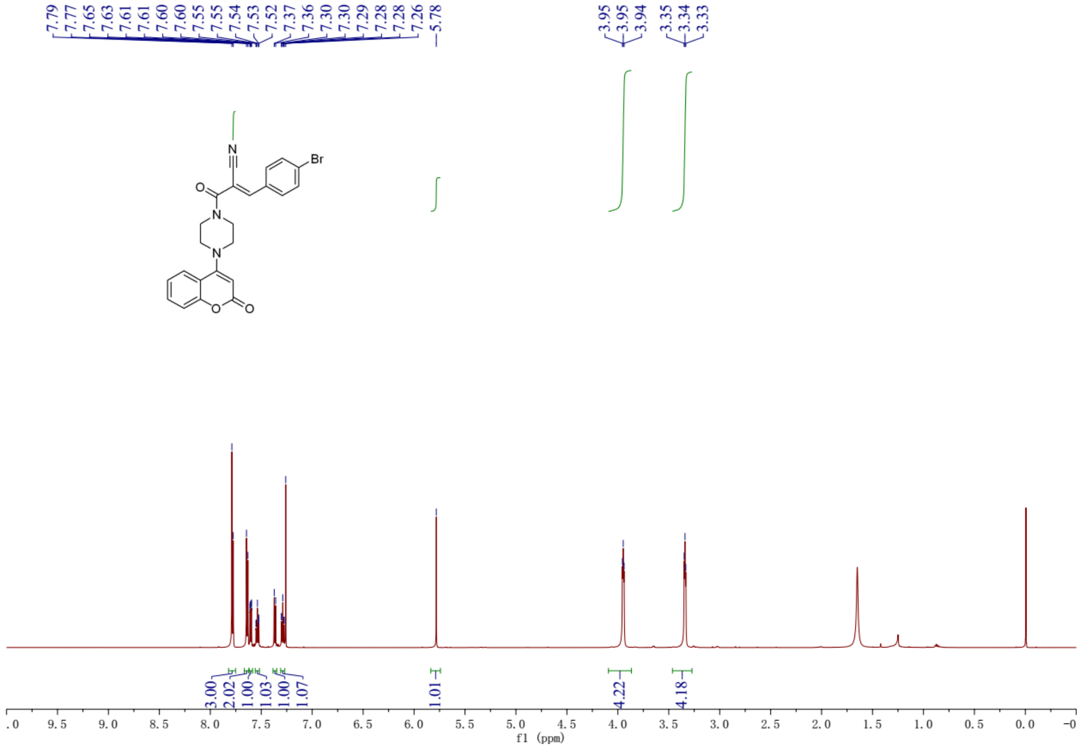


$^{13}$C NMR spectrum of **5r**

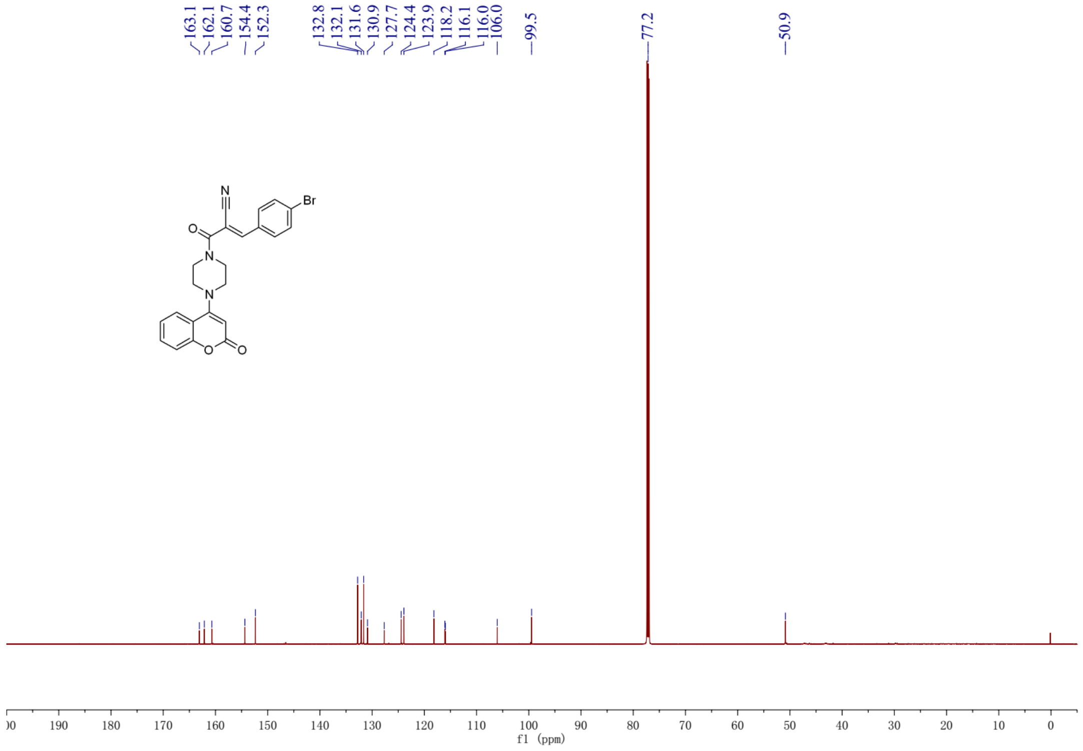

HR-MS (ESI) spectrum of **5r**

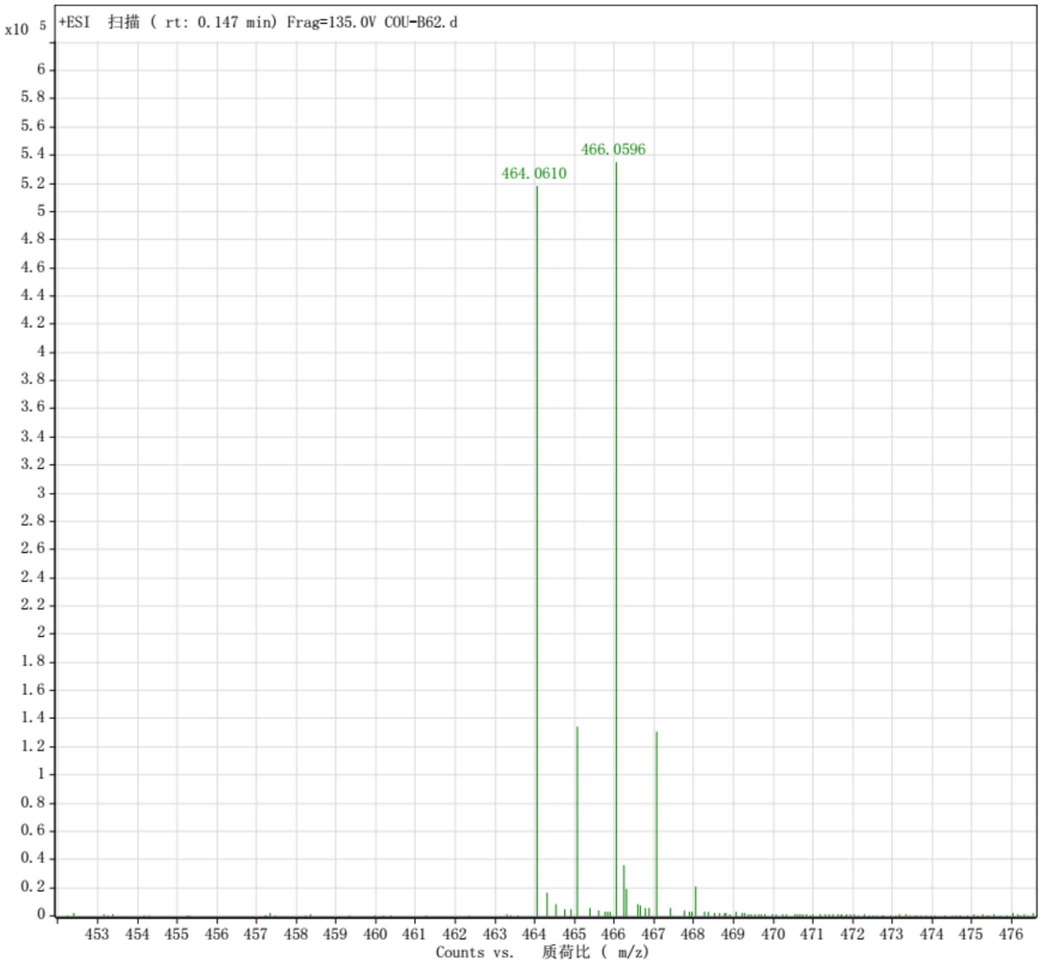

$^{1}$H NMR spectrum of **5s**

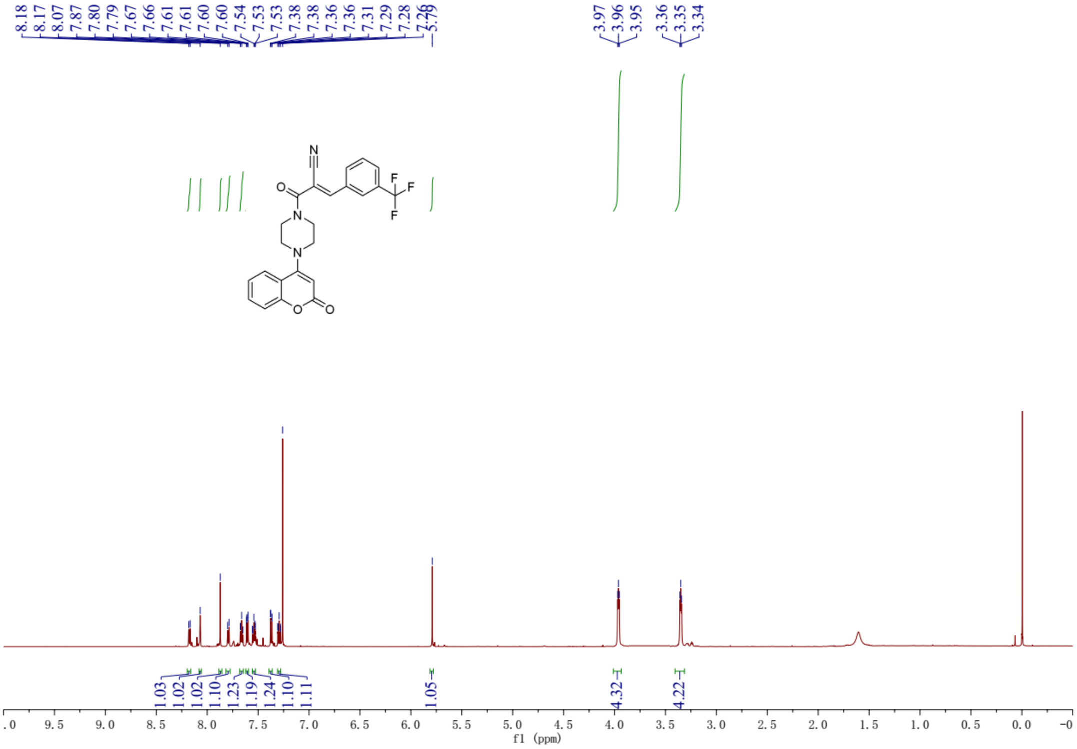

$^{13}$C NMR spectrum of **5s**

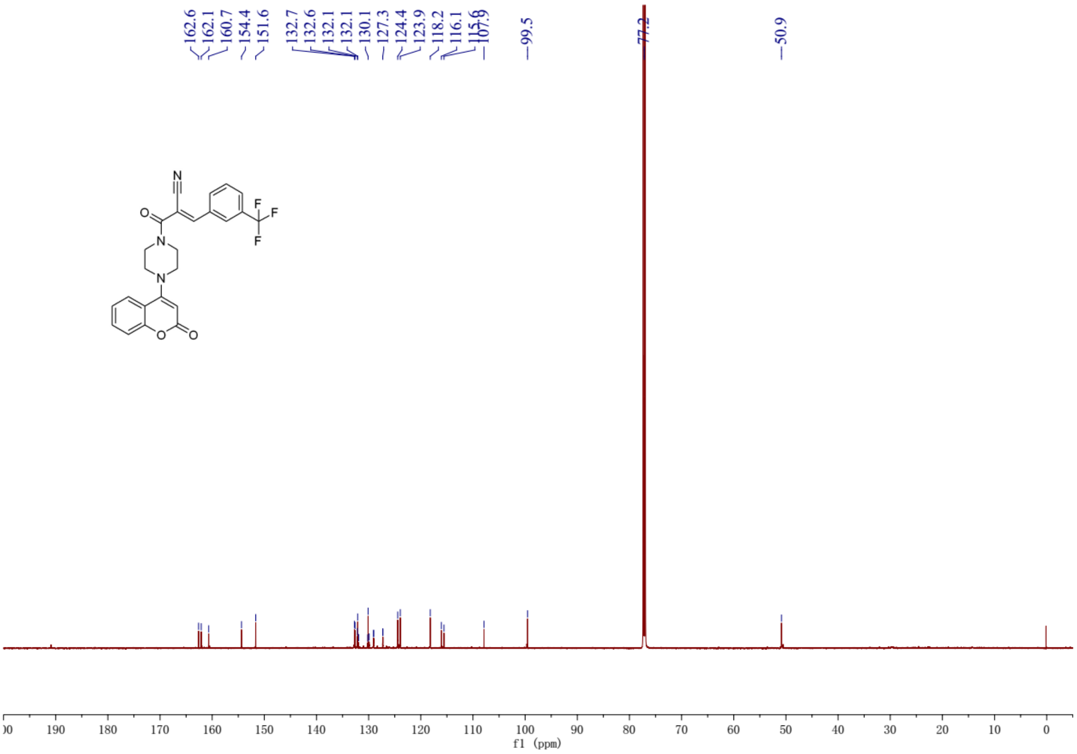

HR-MS (ESI) spectrum of **5s**

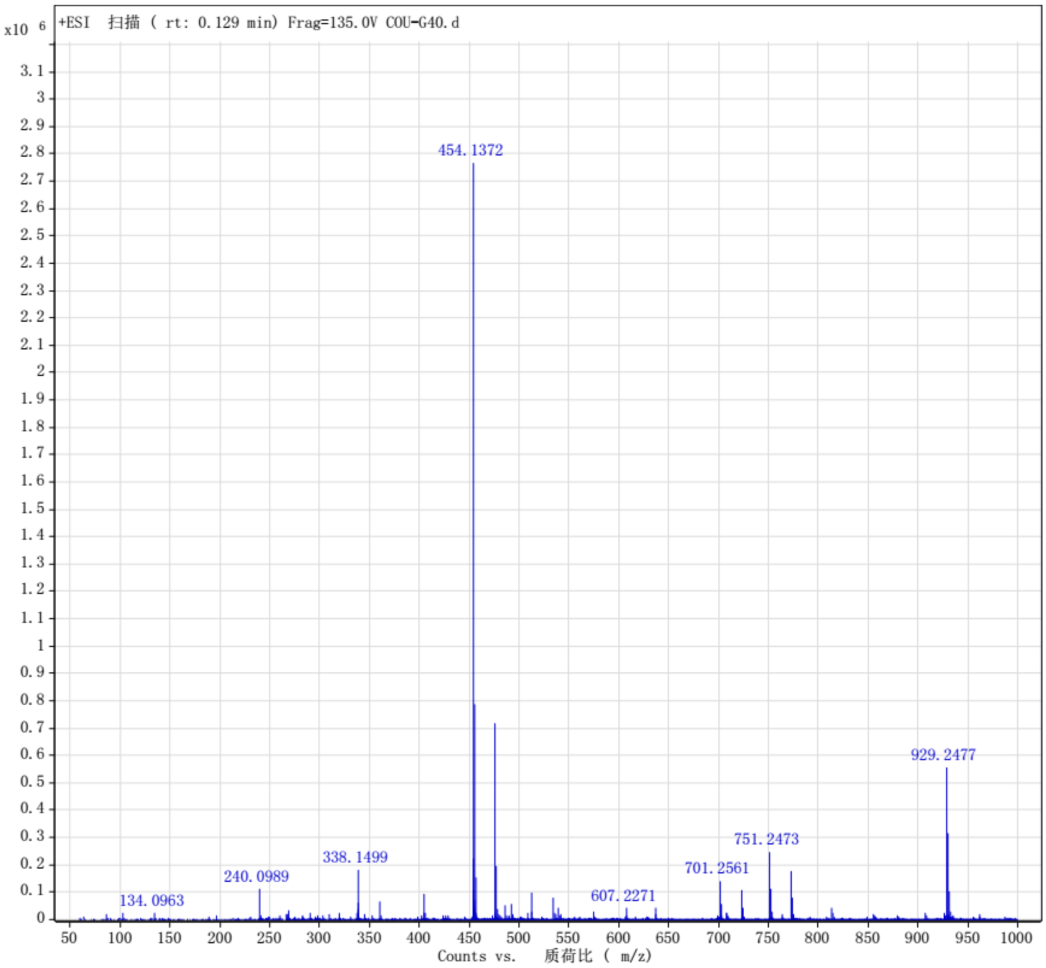

$^{1}$H NMR spectrum of **5t**

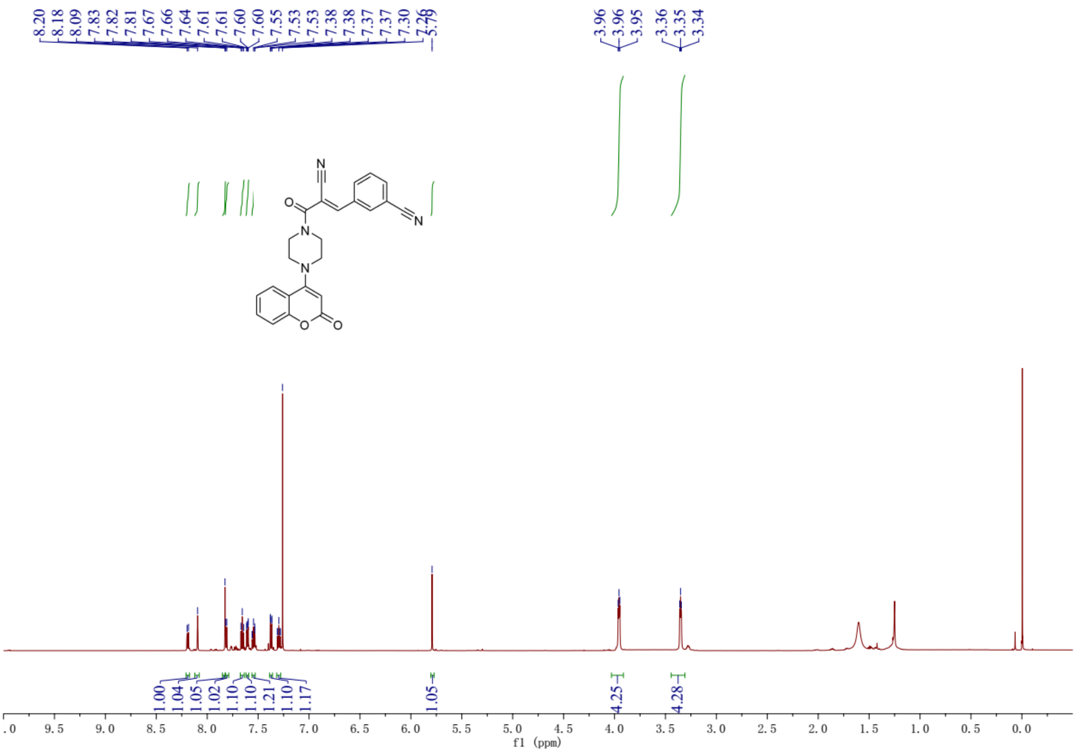

$^{13}$C NMR spectrum of **5t**

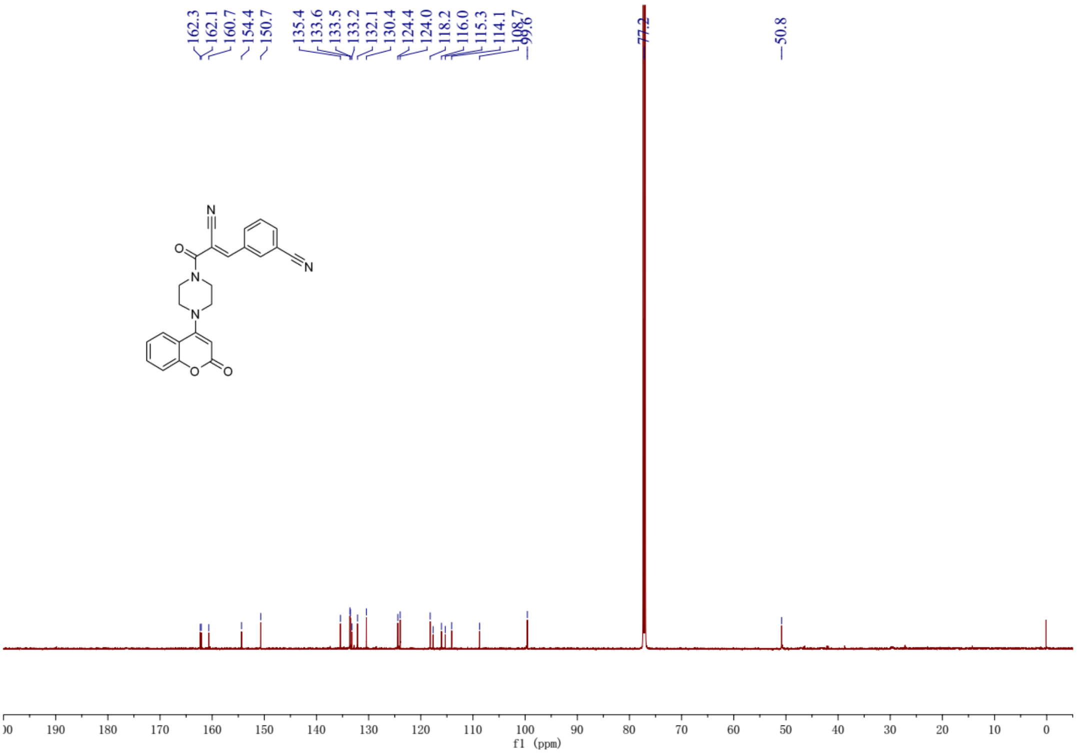

HR-MS (ESI) spectrum of **5t**

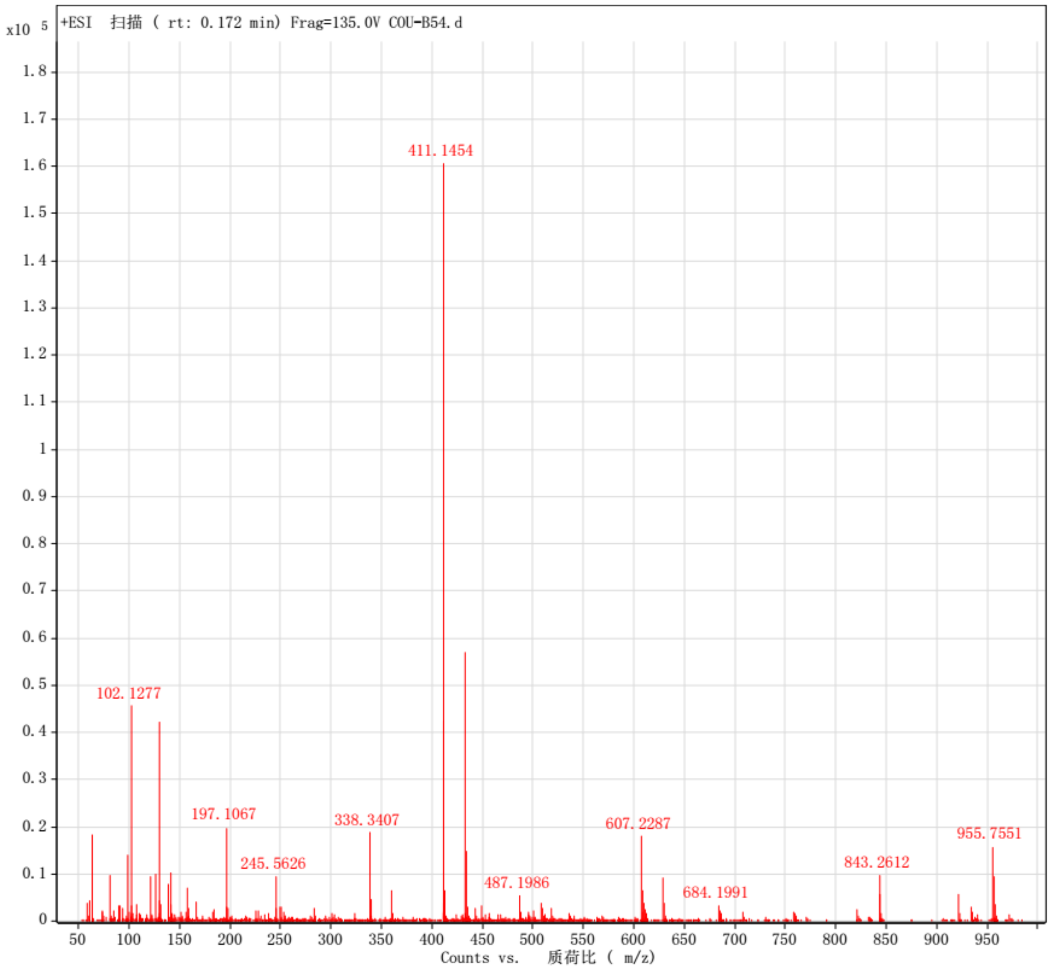

<sup></sup>

$^{1}$H NMR spectrum of **5u**

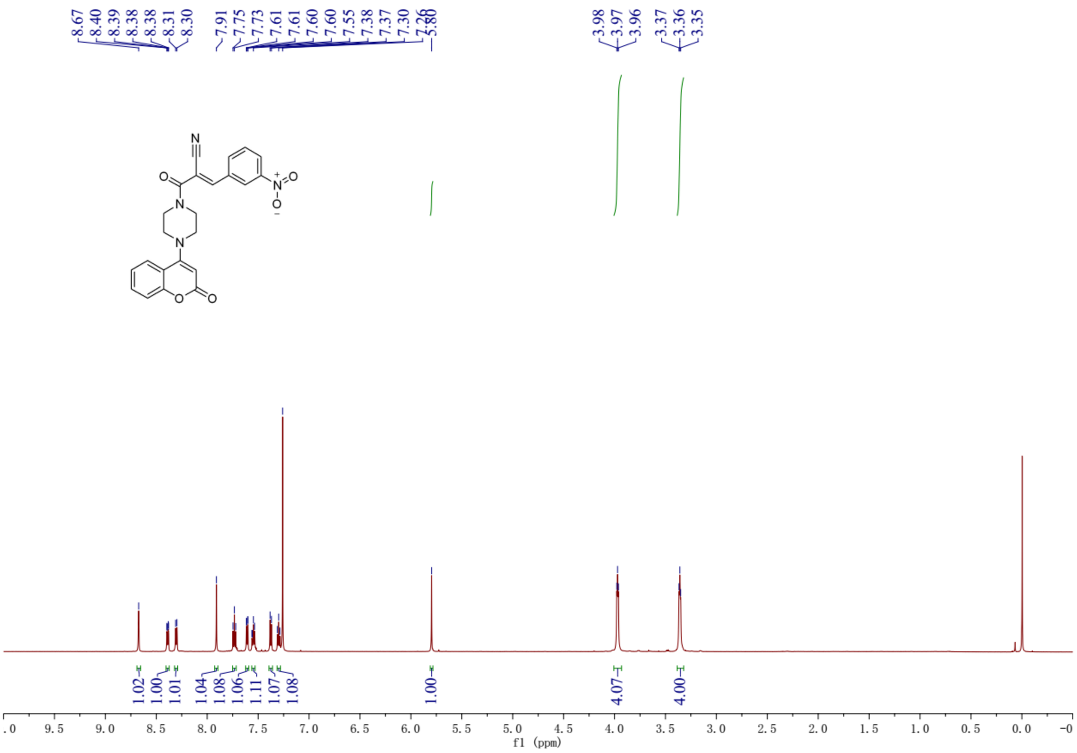

$^{13}$C NMR spectrum of **5u**

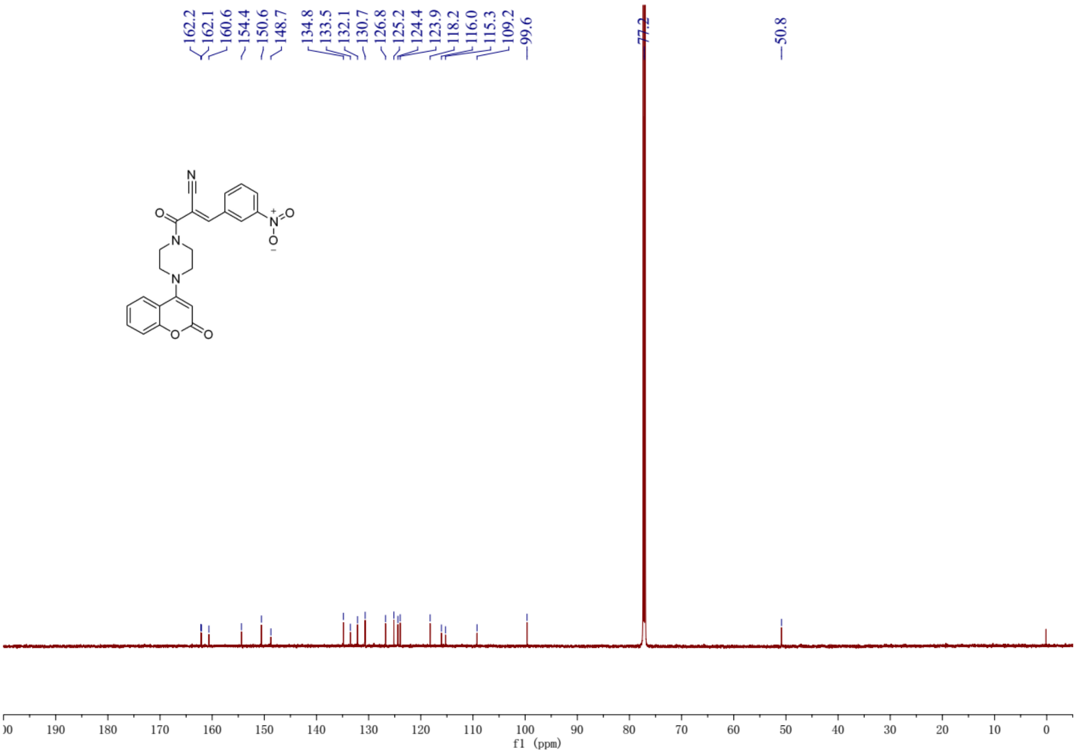

HR-MS (ESI) spectrum of **5u**

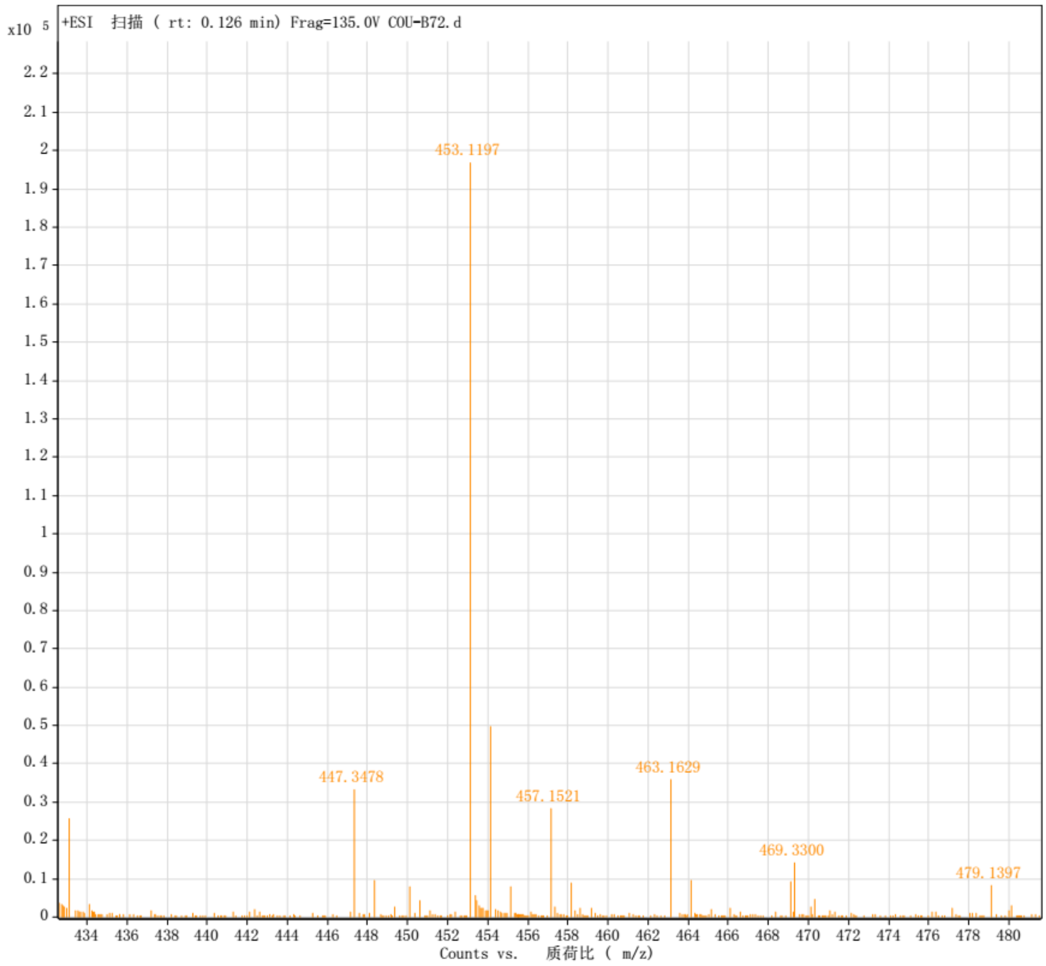

$^{1}$H NMR spectrum of **6a**

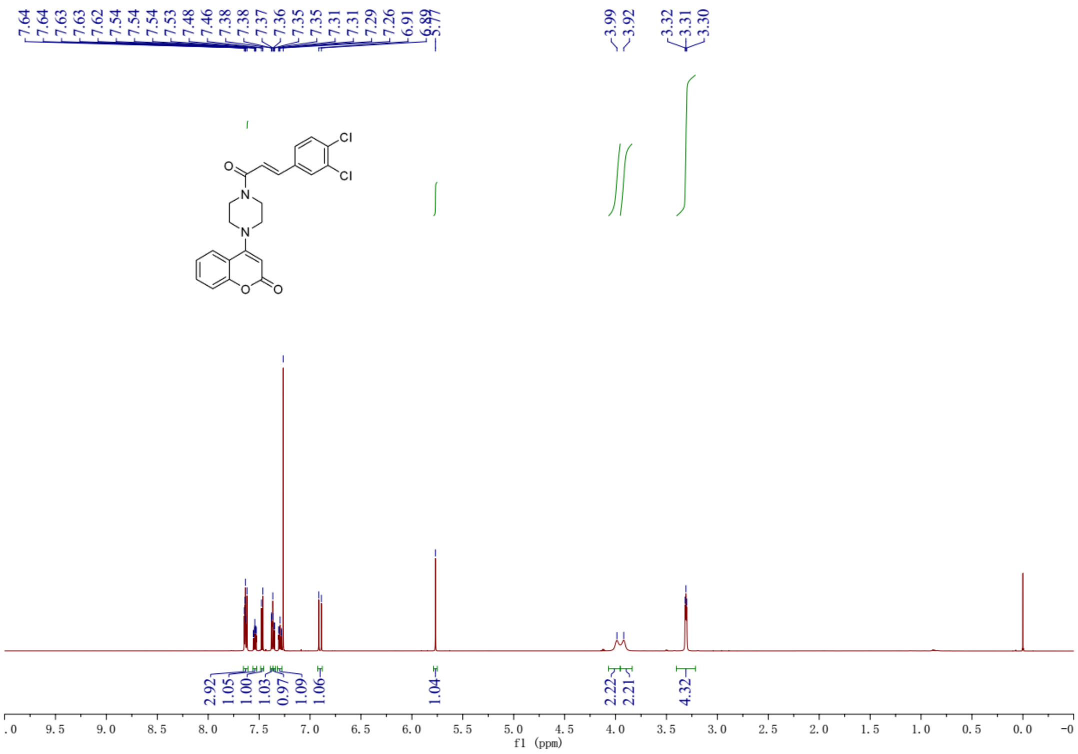

$^{13}$C NMR spectrum of **6a**

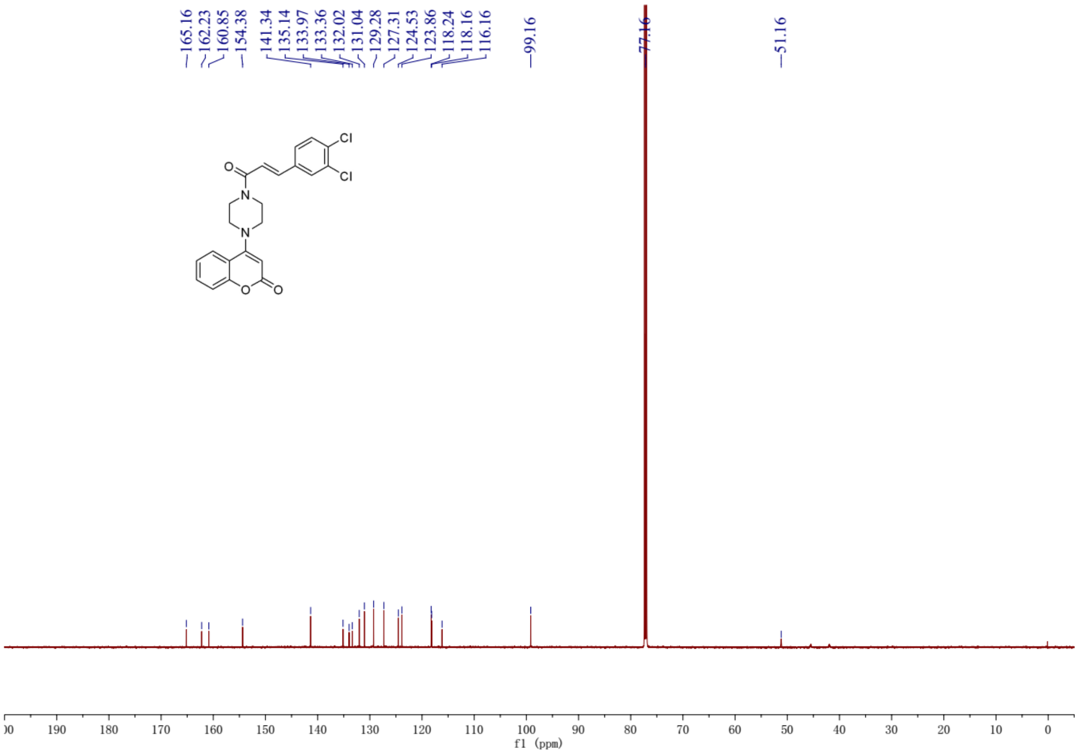

HR-MS (ESI) spectrum of **6a**

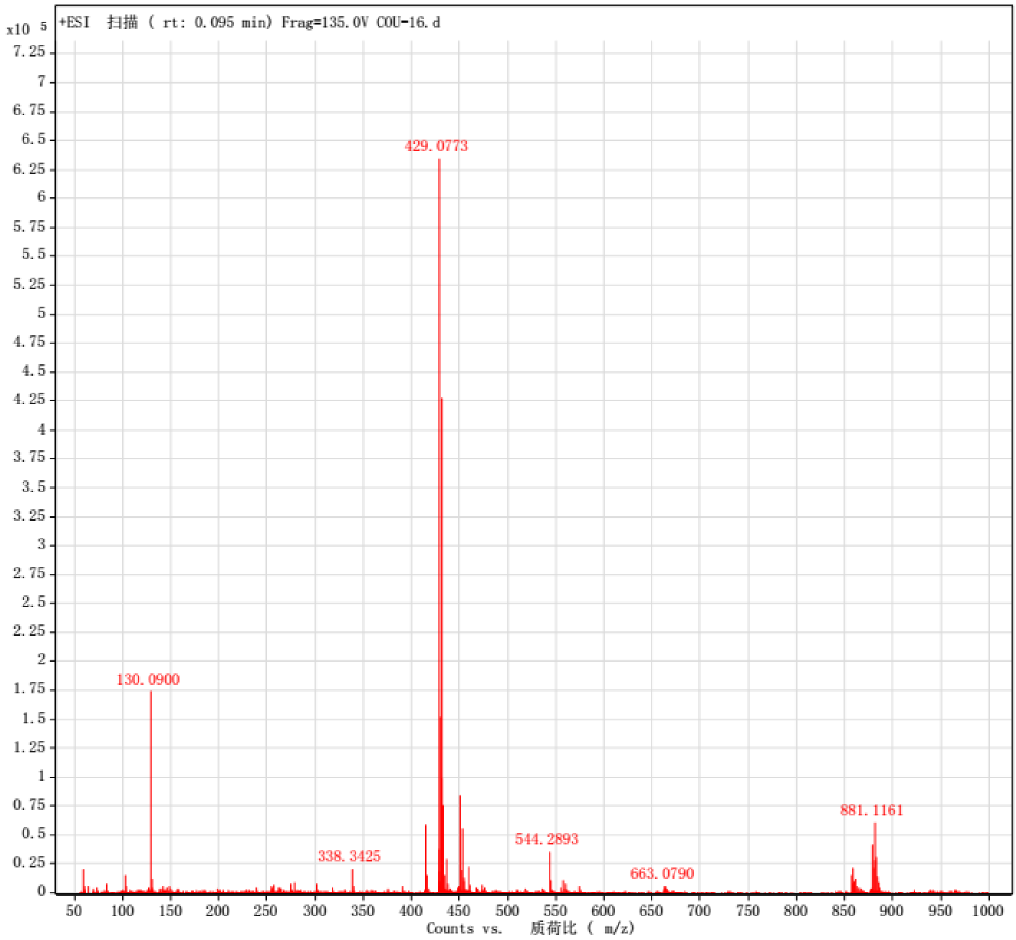

$^{1}$H NMR spectrum of **6b**

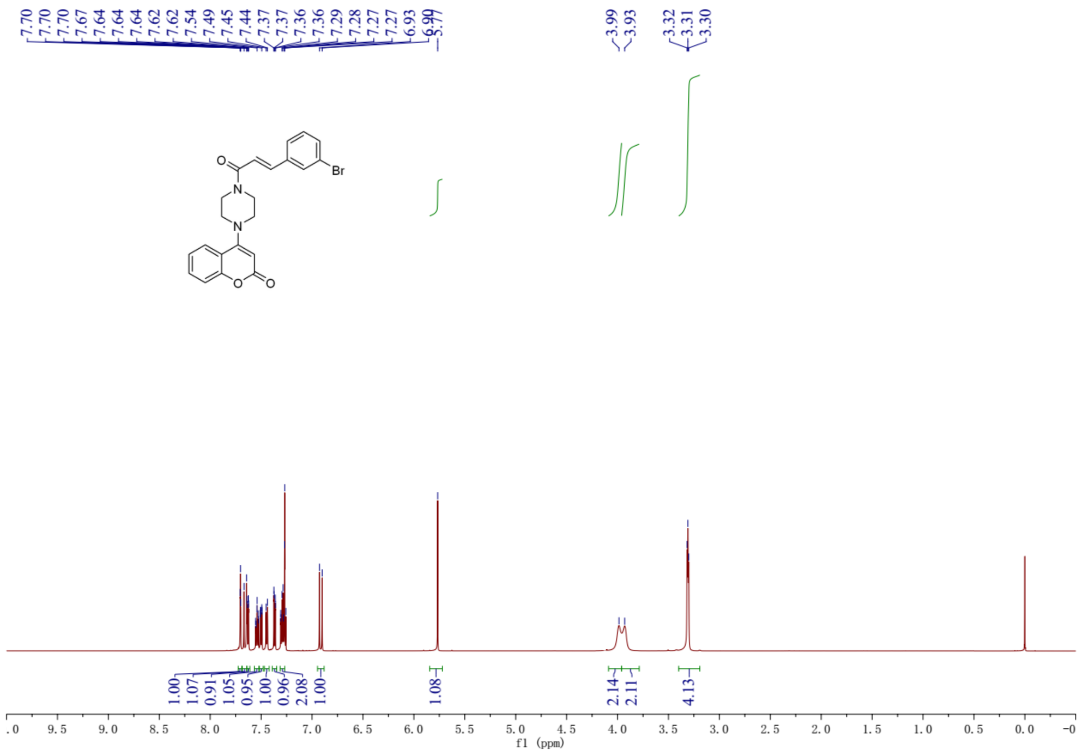

$^{13}$C NMR spectrum of **6b**

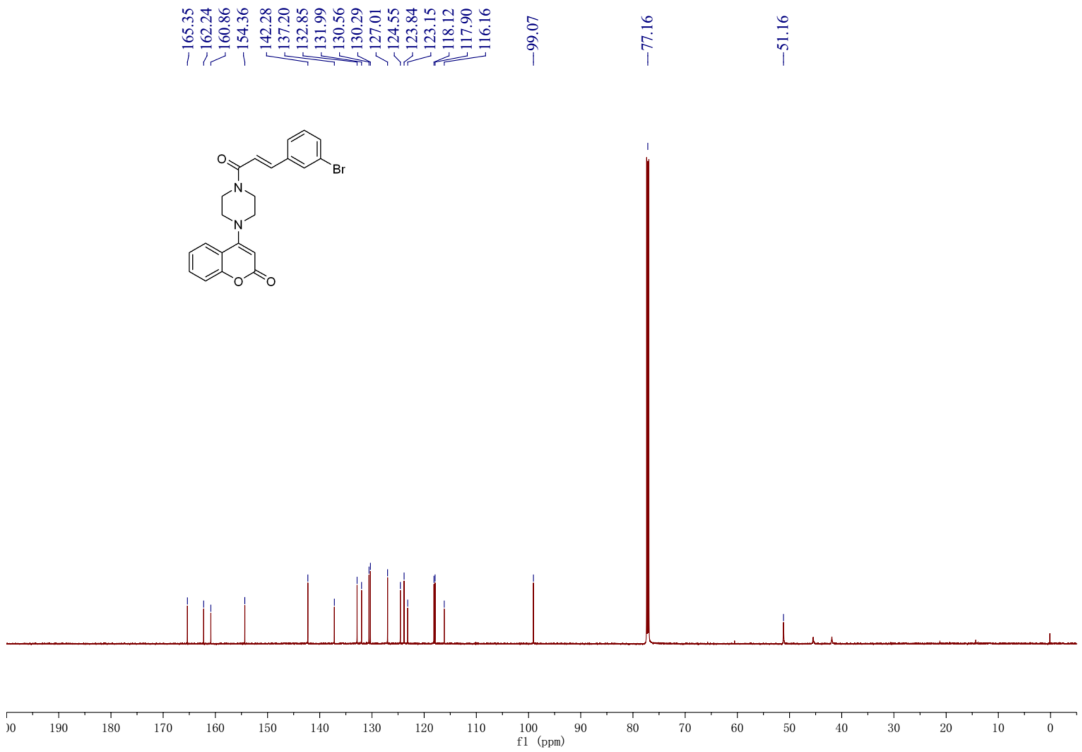

HR-MS (ESI) spectrum of **6b**

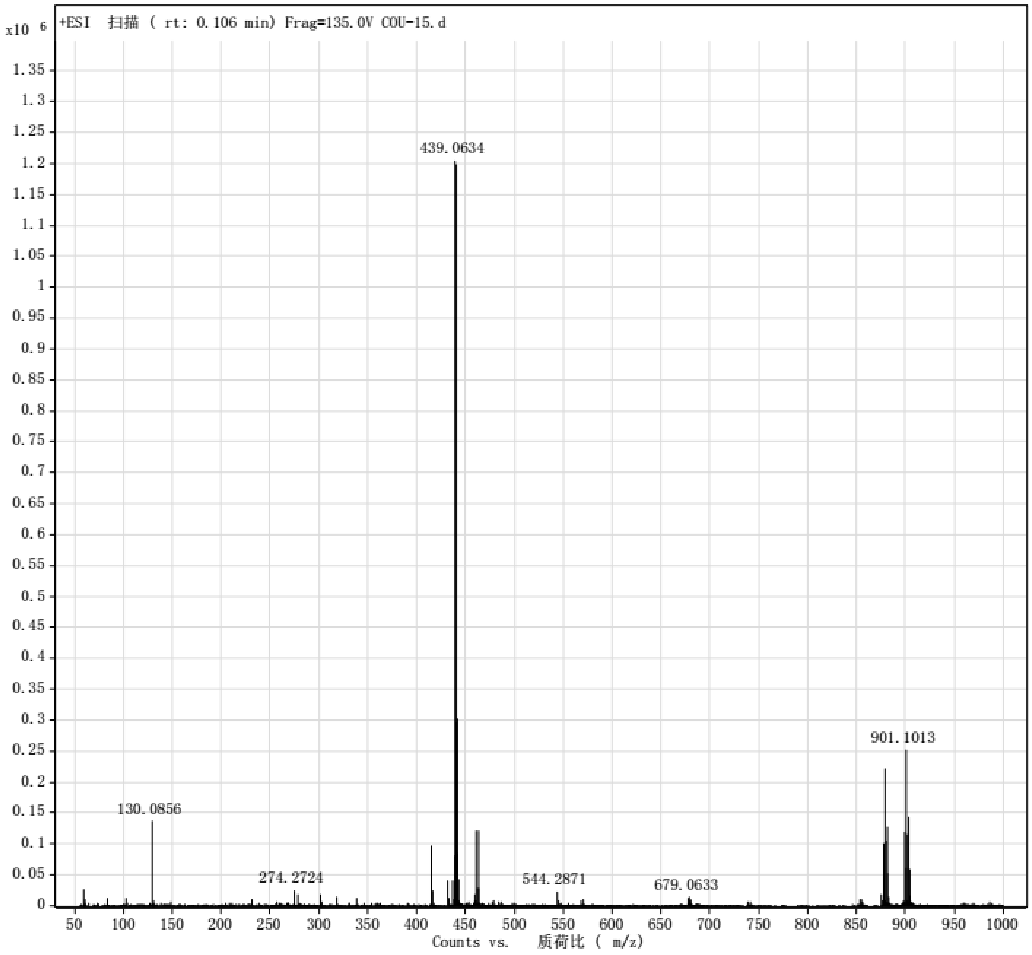

Supplement: Supplementary file 1 [file molecules-23-01972-s001.pdf]
